# Plant growth-promoting bacteria from Uzungöl forest stimulate rice growth via seed biopriming and root inoculation: isolation and functional characterization of potent PGPR strains from rhizosphere soils of different trees

**DOI:** 10.3389/fpls.2025.1622951

**Published:** 2025-07-24

**Authors:** Saad Ishaq, Ali Osman Belduz, Esma Ceylan, Aleyna Nalcaoglu Senocak, Wajeeha Munawar, Amir Towfiq Hasan Alkowlani, Rabiye Terzi, Kadriye Inan Bektas, Sabriye Canakci

**Affiliations:** ^1^ Department of Biology, Faculty of Sciences, Karadeniz Technical University, Trabzon, Türkiye; ^2^ Department of Molecular Biology and Genetics, Faculty of Sciences, Karadeniz Technical University, Trabzon, Türkiye

**Keywords:** biofertilizer, bioinoculant, biopriming, hydroponics, PGPR, rhizosphere, rice, Uzungöl forest

## Abstract

**Introduction:**

Rhizobacteria naturally promote plant growth and offer a sustainable alternative to agrochemicals. In contrast to agroecosystems, forests host a diverse community of beneficial rhizobacteria that remains uncharacterized. Moreover, despite extensive research on rhizobacteria associated with cereal crops, such as rice, their efficacy in hydroponic rice cultivation still needs to be established.

**Methods:**

This study was aimed to isolate, characterize, and identify the potential plant growth-promoting rhizobacteria (PGPR) of different rhizospheres from Uzungöl forest situated in Trabzon, Turkey, and to evaluate their effects on the growth of rice through two distinct approaches: 1) seed biopriming to assess germination and 2) root inoculation to analyze seedling growth in a hydroponic system.

**Results:**

In total, 129 bacteria were isolated from eight different rhizospheres, and 109 exhibited indole-acetic acid (IAA) production. A strain of *Bacillus altitudinis* from the *Acer pseudoplatanus* rhizosphere produced the highest (739.9 ± 251.5 µg/mL) IAA. Siderophore formation was exhibited by 16 isolates including the strains of *Lysinibacillus fusiformis*, *Microbacterium phyllosphaerae*, and *Lelliottia* sp. Phosphate solubilization was observed only in nine isolates including the strains of *Acinetobacter calcoaceticus* and *Lelliottia* sp. Furthermore, 65 isolates including the strains of *Herbaspirillum huttiense*, *Lelliottia amnigena*, *Bacillus altitudinis*, and *A. calcoaceticus* were identified as potential endogenous nitrogen-fixing diazotrophs for rice. Various isolates exhibited salt tolerance, HCN, ammonia, and hydrolytic enzyme production. Several of these PGPR strains as well as the strains of *Viridibacillus arenosi*, *Psychrobacillus faecigallinarum*, *Bacillus siamensis*, *Micrococcus luteus*, and *Staphylococcus succinus* demonstrated positive effects on rice germination or seedling growth. *Herbaspirillum huttiense* strain S1(E) from *Abies nordmanniana* rhizosphere and *Pseudomonas mohnii* strain SS7(5) from *Malus domestica* rhizosphere exhibited outstanding response as seed biopriming agents and root inoculants for rice.

**Discussion:**

These findings concluded that inoculation with forest-derived rhizobacteria is an effective strategy to enhance early growth of rice in soilless systems. Understanding the genetic basis of their growth promotion, coupled with large-scale field validation, could advance low-cost, sustainable rice cultivation with minimal reliance on agrochemicals.

## Highlights

- Rhizobacteria from Uzungöl forest exhibit multiple PGP traits of potential biofertilizers and growth stimulators, offering eco-friendly substitutes to synthetic inputs in rice cultivation.
- *Bacillus altitudinis* strain R4(1)2 from the *Acer pseudoplatanus* rhizosphere demonstrated the highest IAA production (739.9 ± 251.5 µg/mL), along with other beneficial traits.
- Both seed biopriming and root inoculation with *Herbaspirillum huttiense* strain S1(E) and *Pseudomonas mohnii* strain SS7(5) significantly improved rice growth.
- Several isolates exhibited nitrogen fixation, phosphate solubilization, siderophore production, and hydrolytic enzyme activity.
- The study demonstrated an effective way to isolate PGPR strains from forest ecosystems, having potential applications in sustainable agriculture.

## Introduction

Microbial fertility of soils is considered to be important, as up to 90% soil processes are mediated by soil microbes (Philippot et al., 2024). Forest soils inhabit diverse microbial communities varying across different rhizospheres, performing multiple functions which are vital for the sustainability of ecosystems (Lladó et al., 2017). Microorganisms such as bacteria play an important role in organic matter decomposition, nutrient cycling, and bioremediation, which promote plant growth (Yadav et al., 2021). In addition, plants exhibit a diverse array of interactions with these microbes in the rhizosphere, phyllosphere, and endosphere compartments (Hassani et al., 2018). Variations in the composition of root exudates across different rhizospheres influence microbial membership and their respective functions (Seitz et al., 2022). Therefore, it is important to estimate up to what extent a particular microbial profile of a specific rhizosphere holds beneficial traits for plant growth promotion.

A diverse group of bacteria known as plant growth-promoting rhizobacteria (PGPR) inhabits the rhizosphere of plants possessing multiple beneficial functions of biofertilizers, phytostimulators, bioremediators, and biopesticides (Saeed et al., 2021; Gafur et al., 2024). Rhizobacteria can stimulate plant growth by producing plant hormones such as auxins (Lata et al., 2024), gibberellins, and cytokinins. These microorganisms help improve the availability of nutrients (N, P, Fe), particularly through nitrogen fixation, ammonia production, calcium phosphate solubilization, and siderophore production (Kang et al., 2009; Rana et al., 2023; Quadriya et al., 2024). Rhizobacteria suppress plant pathogens through the production of antimicrobial compounds such as hydrocyanic acid (HCN) and siderophore (Chen et al., 2023; Mir et al., 2022). They elevate soil health by improving soil structure and microbial diversity (Saeed et al., 2021).

PGPR can also modulate various physiological and biochemical processes in plants (Sheteiwy et al., 2023). They enhance photosynthetic efficiency, increase leaf area, and stimulate chlorophyll pigment synthesis. PGPR also activate antioxidant defense mechanisms by upregulating enzymes, thereby mitigating oxidative damage caused by reactive oxygen species (Batool et al., 2020). Inoculation with PGPR enhances plant stress resilience by promoting the accumulation of osmoprotectant compounds such as soluble sugars, amino acids, flavonoids, proline, and carotenoids (Nader et al., 2024; Gowtham et al., 2022). Biofortification with the application of PGPR elevates primary and secondary metabolites, which helps increase the nutritional and biological value of different crops (Mahmoud et al., 2024, 2025).

The application of these PGPR for the management of forest and agriculture ecosystems has gained prominent attention (Saha et al., 2023). A substantial number of diverse PGPR from the fertile rhizospheres of various crops across multiple agroecosystems have been isolated. These PGPR have demonstrated efficacy as biofertilizers, phytostimulators, bioremediators, and biopesticides in different experiments (Pappalettere et al., 2024; Liang et al., 2024; Vitorino et al., 2024; Mir et al., 2022; Habibi et al., 2014). However, a significant gap exists in isolating, screening, and characterizing these microorganisms from forest ecosystems and evaluating their effectiveness in promoting the growth of crop plants.

The use of such bacteria is eco-friendly for nutrient and disease management in major crops like rice, providing an advantage over the use of chemical fertilizers, pesticides, and antistress products, which have high cost and environmental concerns (Habibi et al., 2014; Mir et al., 2022; Harahap et al., 2023; Barros-Rodríguez et al., 2021). On the other hand, to meet the global demand for rice, there is a need to increase its productivity, which is impossible without using synthetic fertilizers and pesticides (Bin Rahman and Zhang, 2023). This situation highlights the urgency of adopting innovative, eco-friendly crop management strategies that endorse long-term sustainability without compromising yield.

Hydroponic systems offer high resource use efficiency by promoting optimal growth conditions with minimum water and fertilizer use. Still, optimizing plant growth in these systems remains a challenge. Inoculating plants with PGPR in these systems provides a promising strategy. However, many soil-derived microbes cannot make a successful transition to hydroponic environments (Stegelmeier et al., 2022; Lee and Lee, 2015).

Screening the diverse microbial communities of forest soils for their beneficial functions is crucial to develop environment-friendly agro-products (Hernández-León et al., 2022). Uzungöl forest, located in Trabzon, Turkey, is a stable, biodiversity-rich ecosystem comprised of densely populated coniferous trees and fertile soil, providing a rich resource bank for beneficial microbes (Terzioğlu et al., 2015). Isolation and functional characterization of these microorganisms from different rhizospheric soils will help to understand their role in stabilizing the forest vegetation. It will also contribute to screen out the microorganisms with multiple plant growth-promoting traits.

The aims of the current study include the isolation, characterization, and identification of PGPR present in rhizosphere soils of different trees from the Uzungöl forest. These microorganisms will also be tested for their effectiveness in promoting the germination and growth of rice.

This study will not only expand our ecological understanding of forest-derived PGPR through their functional characterization but also open new avenues for their application in sustainable, low-input rice cultivation by validating their use in seed biopriming and root inoculation. It will explore the forest microbial diversity, rarely studied for agricultural use, and increase our knowledge on the efficacy, compatibility, and functionality of PGPR in soilless media, particularly hydroponics. We hypothesize that forest-derived PGPR possess the characteristics of growth-stimulating bioinoculants for rice, due to their multiple plant-beneficial traits that remain effective beyond their native habitat and their robust ability to adapt to a hydroponic environment.

## Methodology

### Soil sampling

**Figure 1:**
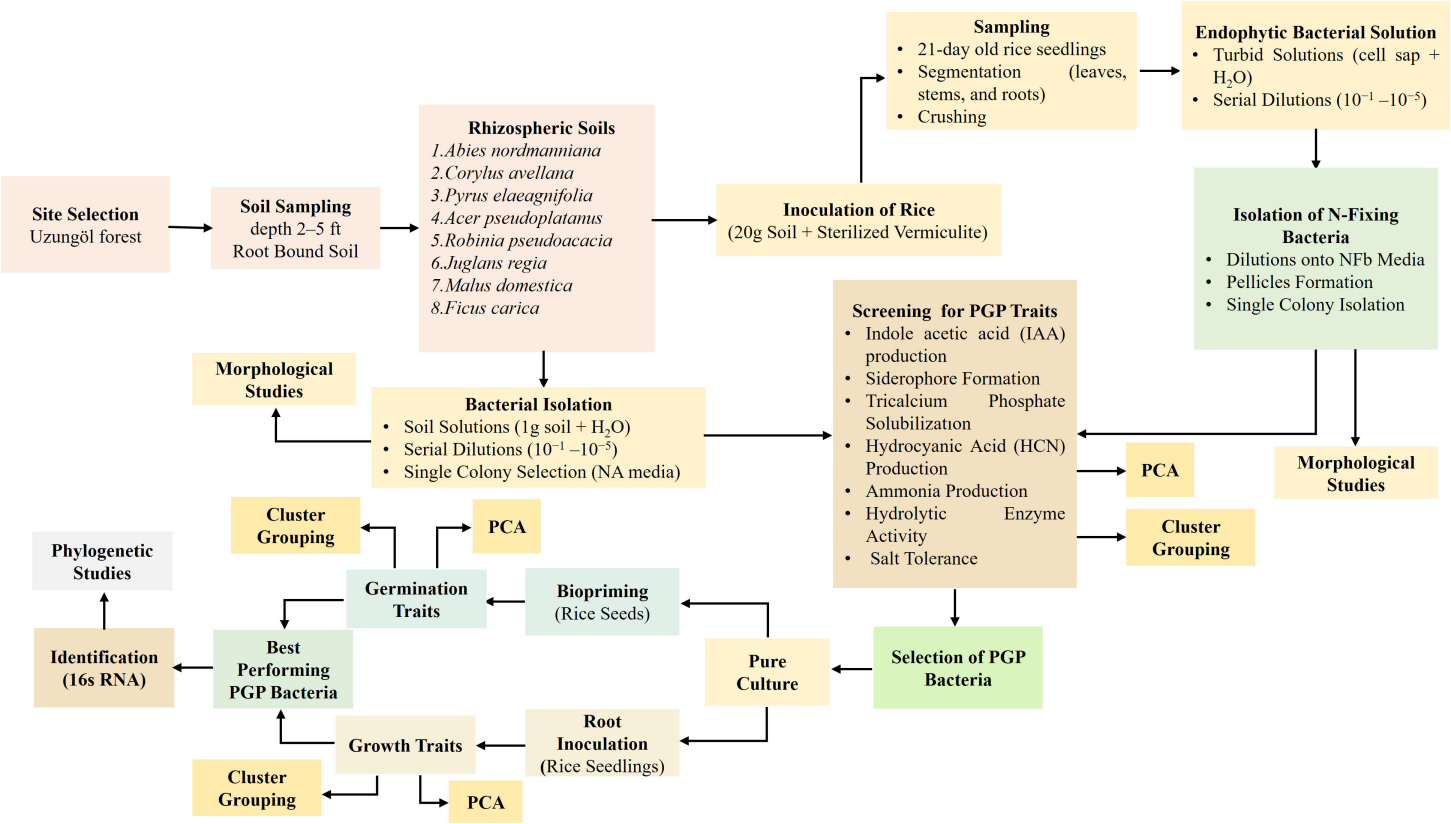
Flow diagram illustrating the procedure to isolate and characterize the PGPR from different rhizosphere soils and their application to screen out the best strains for rice seed biopriming and root inoculation.

**Figure 2:**
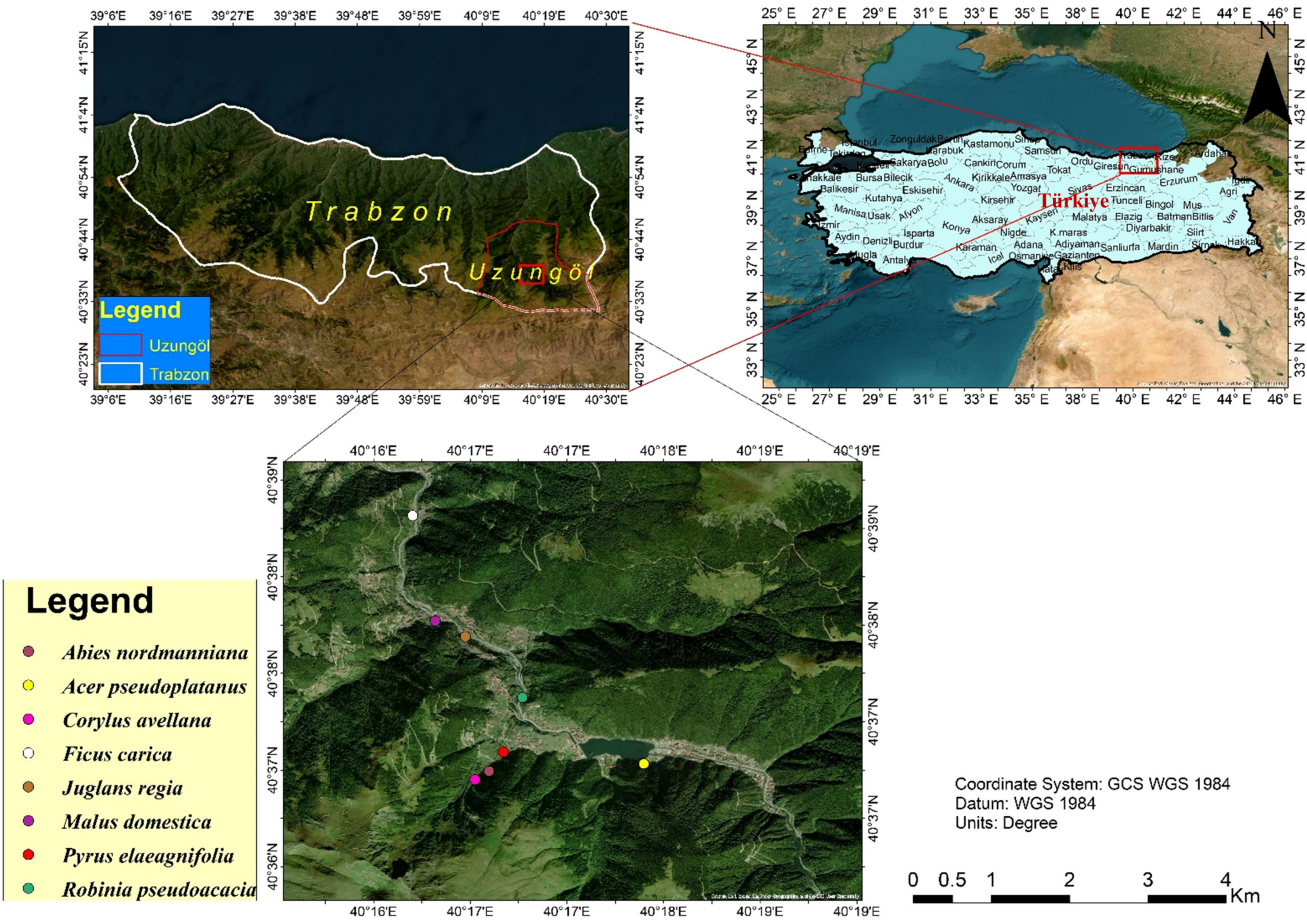
Map indicating the soil sample collection sites within Uzungöl forest, Trabzon, located in the Black Sea region of Turkey.

Soil samples were gathered from rhizospheres of eight different trees (*Abies nordmanniana*, *Corylus avellana*, *Pyrus elaeagnifolia*, *Acer pseudoplatanus*, *Robinia pseudoacacia*, *Juglans regia*, *Malus domestica*, *Ficus carica*) at a soil depth of 2–5 ft within different locations (**Table 1**, **Figures 1**, **2**) of Uzungöl forest, Trabzon, Turkey. The soil bound to the root surface was extracted and identified as a potential habitat of PGPR bacteria. The soil samples were kept in polyethylene bags and stored at −20°C for later analysis.

**Table 1:** Description of soil sample collection and the respective number of isolates obtained from each sample.

Rhizosphere soil sample	Coordinates	Total number of isolates
*Abies nordmanniana*	40°36′59.6″N, 40°16′47.7″E	19
*Corylus avellane*	40°36′56.0″N, 40°16′41.9″E	12
*Pyrus elaeagnifolia*	40°37′07.4″N, 40°16′53.7″E	29
*Acer pseudoplatanus*	40°37′02.6″N, 40°17′51.8″E	20
*Robinia pseudoacacia*	40°37′30.0″N, 40°17′01.6″E	14
*Juglans regia*	40°37′55.2″N, 40°16′37.9″E	14
*Malus domestica*	40°38′01.7″N, 40°16′25.6″E	14
*Ficus carica*	40°38′45.3″N, 40°16′16.2″E	7

### Isolation of bacteria from soil samples

To isolate PGP bacteria, 1 g of the soil sample was dissolved in sterilized distilled water, followed by creating serial dilutions (10^−1^–10^–5^) of the soil solutions. From each dilution, 30 μL was spread onto nutrient agar (NA) medium plates, which were then incubated at 30°C for 2 days. After incubation, diverse bacterial colonies differing in shape, color, and size appeared on NA plates. Single bacterial colonies with distinct morphological characteristics were then selected on the basis of colony form, elevation, margin, and color and were transferred to fresh NA plates through restreaking. Individual colonies were then preserved on NA slants at 4°C and stored in 30% glycerol stocks at −80°C for later studies (**Figure 1**).

### Isolation of potential endophytic nitrogen-fixing bacteria of rice

To isolate endophytic bacteria, 20 g of rhizosphere soil from each sample was taken in separate pots and mixed with sterilized vermiculite (**Figure 1**). Seeds of the Kocamaninci rice variety were sterilized by washing with 70% ethanol for 30 s, followed by 3 min of dipping in 3% sodium hypochlorite solution, and then washed five times with sterilized distilled water. Sterilized rice seeds were sown in trapezoidal pots (6.5 cm top width × 4.5 cm bottom width × 6.5 cm height) containing soil inoculum from a specific rhizosphere. These pots were placed in a growth chamber under controlled conditions (16-h light/8-h dark cycle and day/night temperatures of 25°C/18°C). After 21 days, plants were harvested, rinsed in tap water to eliminate vermiculite, and then washed five times with sterile distilled water within a laminar flow cabinet. Leaves, stems, and roots were segmented into 1–2 cm pieces and crushed in a sterile mortar and pestle. The resulting turbid solution was serially diluted (10^−1^–10^−5^) using sterile distilled water. A loop from each diluted sample was then transferred into a screw-capped tube containing 4 mL of nitrogen-free bromothymol blue (NFB) semisolid media (Habibi et al., 2014; Baldani et al., 2014). Tubes were incubated at 30°C for 1 week, during which bacterial growth appeared as veil-like pellicles. Subsequently, 100 μL from each pellicle layer was further diluted (10^−1^–10^−5^), and 30 μL from each dilution was spread onto NFB agar plates. These plates were then incubated at 30°C for 2 days in an anaerobic jar, after which distinct colonies were formed. Single colonies were selected and transferred to fresh NFB agar plates by streaking. The purified cultures were preserved in nutrient agar slants at 4°C and stored in 30% glycerol stocks at −80°C for later studies.

### Morphological studies of bacterial isolates

To observe the morphological features of isolates, bacterial colonies were cultured on NA agar plates. A loop from each colony was diluted with 1 mL of sterilized distilled water in a microtube and mixed well. Subsequently, 5 μL of this mixture was spread onto an NA agar plate and incubated at 30°C for 3 days. The morphological characteristics of bacteria, such as colony shape, elevation, edge, and color, were observed under a stereo microscope (**Figure 1**).

### Analysis of indole-3-acetic acid production

**Figure 3:**
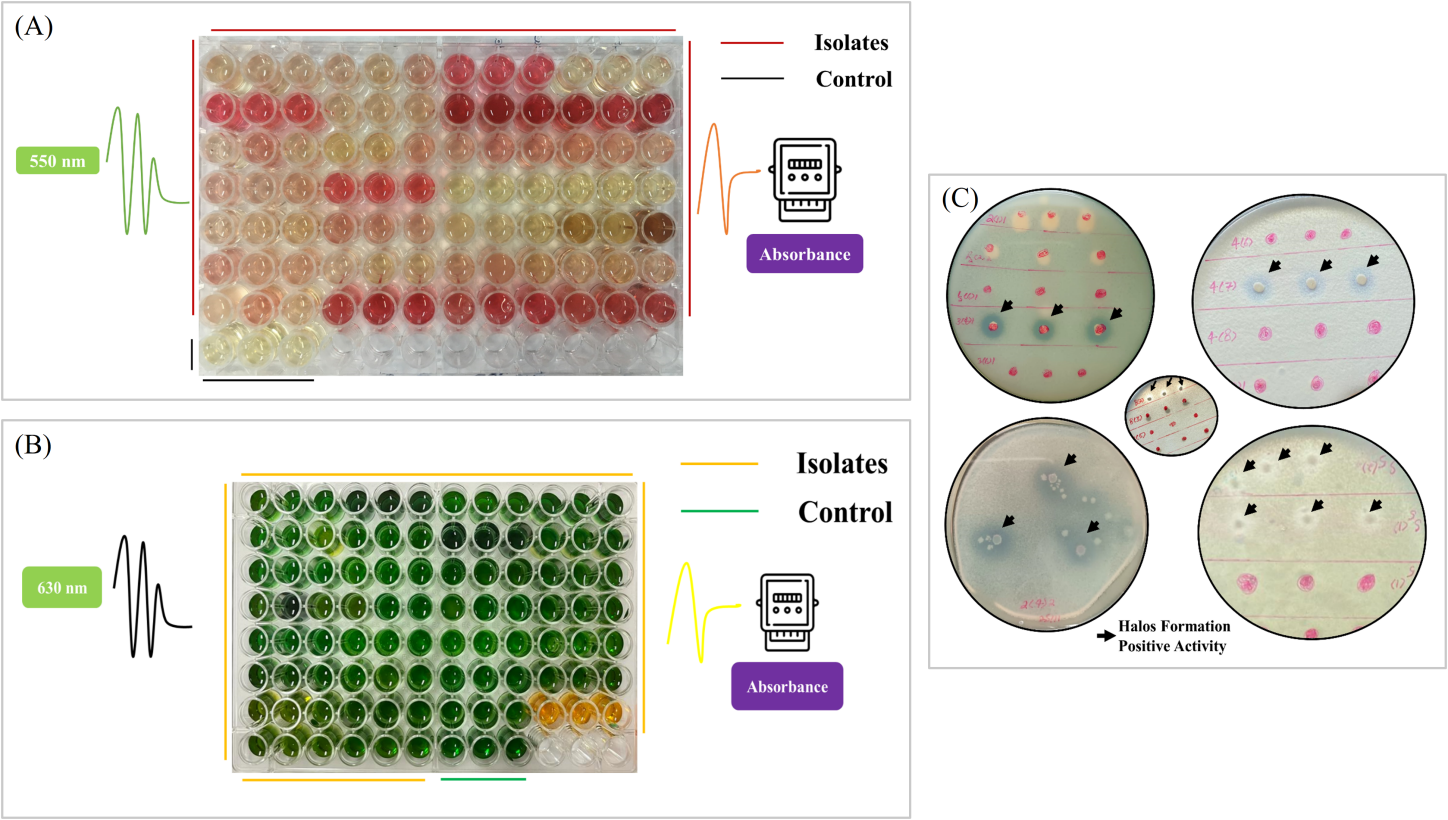
Screening of isolates for plant growth-promoting traits. **(A)** Spectrophotometric detection of indolic compound using a 96-well plate assay. The degree of pink color correlates with the level of indolic compound production: control in the last row indicates no indolic compound, while increased pink intensity among the isolate samples indicates higher production levels. **(B)** Spectrophotometric detection of siderophore production using a 96-well plate assay. A color change from greenish-blue to orange indicates siderophore production. **(C)** Assessment of tricalcium phosphate solubilization using Pikovskaya’s agar supplemented with tricalcium phosphate. Clear zones indicated by arrows around bacterial growth represent the phosphate solubilization ability of the isolate.

For the detection and quantification of indole-3-acetic acid (IAA), each bacterial strain was cultured in a test tube containing 3 mL of King’s B medium (comprising 17.2 mL of 87% glycerol, 1.15 g of K_2_HPO_4_, 20 g of peptone, 0.5 g of L-tryptophan, and 1.5 g of MgSO_4_.7H_2_O per liter). The cultures were incubated for 3 days at 30°C, and then 1 mL from each culture was transferred into a 1.5-mL microcentrifuge tube. The samples were then centrifuged at 5,400×*g* for 10 min, and 500 μL of the supernatant was carefully pipetted into a new microcentrifuge tube. Subsequently, 500 μL of Salkowski reagent was added to each tube and incubated in the dark for 30 min. Then, 300 μL from each tube was transferred to a 96-well plate, and absorbance was measured at 550 nm using a microplate reader (**Figure 3A**). The IAA concentration in the supernatant was determined using an equation developed from a standard curve calibrated against known concentrations of IAA (Abdelwahed et al., 2023).

### Estimation of siderophore formation via modified universal CAS assay

The ability of bacterial strains to produce siderophores was estimated using the universal CAS assay with slight modifications (Arora and Verma, 2017). At first, to eliminate iron contamination, all glassware was rinsed with 3 mol/L of hydrochloric acid (HCl) and then washed with deionized water. The CAS reagent was prepared as follows: 121 mg of CAS was dissolved in 100 mL of distilled water parallel to this, and a 20-mL solution of 1 mM ferric chloride (FeCl_3_.6H_2_O) was prepared in 10 mM of HCl. These two solutions were mixed, and the mixture was then added to 20 mL of a hexadecyl trimethyl ammonium bromide (HDTMA) solution under continuous stirring. The HDTMA solution was prepared by dissolving 729 mg of HDTMA in 400 mL of distilled water. The resulting CAS-HDTMA solution was sterilized prior to use.

For estimating siderophore production, the procedure was carried out using a microtiter plate. Each bacterial isolate was cultured in a test tube containing 3 mL of King’s B medium for 3 days at 30°C. Later, 1 mL from each tube was transferred to a 1.5-mL microcentrifuge tube and centrifuged at 5,400×*g* for 10 min. Then, 100 μL of the supernatant from each bacterial culture was pipetted into separate wells of the microtiter plate, followed by the addition of 100 μL of CAS reagent to each well. The plates were incubated in the dark for 20 min. The optical density of each sample was measured at 630 nm using a microplate reader (**Figure 3B**). Siderophore production was quantified in percent siderophore units (psu), calculated according to the formula (Payne, 1993).


Siderophore production (psu)=(Ar−As×100)/Ar

where Ar is the absorbance of the reference (CAS solution and uninoculated media), and As is the absorbance of the sample (CAS solution and cell-free supernatant of the sample).

### Tricalcium phosphate solubilization activity

Bacterial isolates were cultured in the nutrient broth medium for 48 h at 28°C. Subsequently, 5 μL from each culture was dropped into Pikovskaya’s agar medium supplemented with tricalcium phosphate (Pikovskaya, 1948). These plates were then incubated for 7 days at 28°C. The formation of a clear zone around each bacterial colony indicated the ability of the isolate to solubilize phosphate (**Figure 3C**). In order to estimate the phosphate-solubilizing activity, the size of the clear zone, or halozone, was measured using the formula:


Size of halozone=(Diameter of halozone+Diameter of bacterial growth)/Diameter of bacterial growth

### Assessment of hydrocyanic acid production

The HCN production of bacteria was evaluated using the Bakker and Schippers method. Each isolate was streaked on a nutrient agar plate supplemented with 4.4% glycine. A Whatman filter paper No. 1 wetted with 2% sodium carbonate in 0.5% picric acid solution was attached to the underside of the plate lids. The plates were then sealed with parafilm and incubated for 5 days at 28°C ± 2°C. A color change in the filter paper from yellow to slightly brown, medium brown (++), or strong brown (+++) served as an indicator of hydrocyanic acid production (Bakker and Schippers, 1987).

### Detection of ammonia production

The ability of bacterial isolates to produce ammonia was evaluated using the method described by Di-Benedetto. Briefly, bacterial cultures were inoculated into test tubes filled with 10 mL of peptone water broth and incubated at 30°C for 3 days. Following the incubation, 0.5 mL of Nessler’s reagent was added to each tube. The emergence of a brown color from yellow indicated the production of ammonia (Mir et al., 2022).

### Evaluation for hydrolytic enzyme activity (protease, amylase, cellulase)

**Figure 4:**
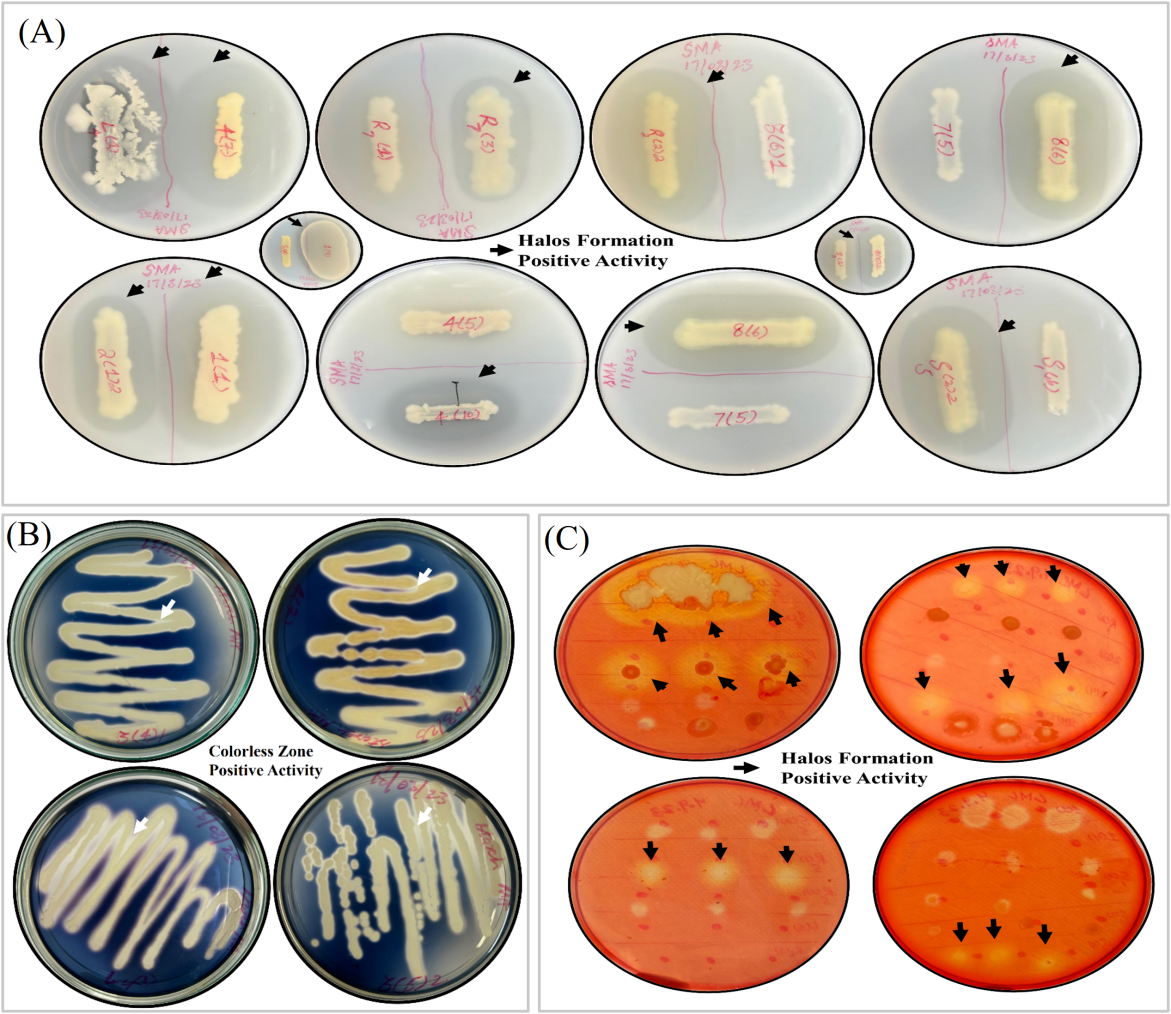
Detection for hydrolytic enzyme activity. **(A)** Estimation of protease activity among bacterial isolates using skim milk agar plates. Halo formation pointed with an arrow around bacterial growth signifies positive protease activity. **(B)** Estimation of amylase activity among bacterial isolates using starch agar plates. The formation of colorless zones pointed by arrows along the bacterial growth represents positive amylase activity. **(C)** Assessment of cellulase activity using the Congo red method. The formation of halos marked by arrows indicates cellulose degradation by bacteria.

Protease activity was assessed by streaking bacterial cultures onto sterile skim milk agar plates. These plates were then incubated for 48–72 h at 30°C. The presence of a halo zone around the bacterial colonies indicated protease production (**Figure 4A**). Protease activity was marked by the formation of a halo zone around bacterial growth. The size of the halo zone indicated the extent of activity (Vermelho et al., 1996).

For the estimation of amylase production, bacterial cultures were streaked onto starch agar medium and incubated at 28°C ± 2°C for 48–72 h. After incubation, the plates were exposed to iodine solution for a minute. The formation of colorless zones around the colonies (**Figure 4B**) indicated amylase activity (Gupta et al., 2003).

Cellulase production was determined using the method of Hendricks. Briefly, 5 μL of actively grown bacterial cultures were spotted onto carboxymethylcellulose Congo red media, containing 0.5 g of carboxymethylcellulose, 0.099 g of K_2_HPO_4_, 0.049 g of MgSO_4_.7H_2_O, 0.05 g of yeast extract, 0.05 g of Congo red, and 20 g of agar per liter and adjusted to pH 7.2. The plates were then incubated for 72 h at 28°C ± 2°C, the plates were then washed with saline water to highlight the clear zones. The formation of a halo zone around bacterial growth (**Figure 4C**) indicated positive cellulase activity (Hendricks et al., 1995).

### Salt tolerance assay

Bacterial isolates were screened for their salt tolerance using nutrient broth (NB) media supplemented with different NaCl (w/v) concentrations, such as 3% (0.513 M), 5% (0.855 M), and 7% (1.196 M). The tubes containing media were inoculated with a loop full of fresh bacterial culture and incubated at 30°C on a shaker (120 rpm). The controls were maintained at 0% (0 M) NaCl (w/v) in NB medium, with and without bacterial inoculation. Bacterial growth in NaCl-supplemented broths was observed and compared with the controls after time intervals of 24 h, 48 h, and 72 h.

### Germination trial and seed biopriming with bacterial isolates

Out of 129 isolates, 41 with significant plant growth-promoting traits were tested in a germination trial to evaluate their effectiveness as seed biopriming agents. Briefly, rice seeds (*Oryza sativa* L.) were surface-sterilized as previously described and then immersed for 30 min in a 2-day-old active bacterial culture having a cell density of 10^9^ colony-forming units/mL (CFU/mL), grown at 28°C in King’s B broth medium. For the control, seeds were soaked in non-inoculated media. From each treatment, seeds were then transferred onto 1% agar plate, and three replications of each treatment were maintained. The plates were initially kept in the dark for 7 days, followed by 3 days under controlled conditions (16-h light/8-h dark cycle at 25°C/18°C day/night temperatures) in a growth chamber. Germination data were recorded at 24-h intervals. At the end of the experiment, seedlings were removed carefully, and measurements were taken for emerging root length (ERL), emerging shoot length (ESL), emerging root mass (ERM), and emerging shoot mass (ESM). Additional parameters like germination energy (GE), seedling vigor index (SVI) 1, and seedling vigor index (SVI) 2 were calculated with the help of data obtained from the germination test using the following equations:


Germination energy (GE)=(No. of seeds germinated after 2 days/total no. of seeds) × 100

Seedling vigor index (SVI)1=(emerging shoot length+emerging root length)×GP

Seedling vigor index (SVI)2=(emerging shoot mass+emerging root mass)×GP

### Plant growth test in a hydroponic culture

Similar to the germination trial, 41 bacterial isolates were also evaluated for their effectiveness as root inoculants to promote the growth of rice seedlings in hydroponic conditions. Briefly, sterilized rice seeds were placed in petri dishes containing wetted filter papers and allowed to germinate. After 7 days, young seedlings with uniform growth were taken and their roots were inoculated for 1 h in a 2-day-old active bacterial culture having a cell density of 10^9^ CFU/mL, grown at 28°C in King’s B broth medium. Seedlings treated with non-inoculated media were used as the baseline control. The seedlings were then transferred onto sterilized plastic boxes (dimensions 17 cm × 9 cm × 4.5 cm) designed for hydroponic culture, and three replications were maintained for each treatment in a completely randomized design (CRD) and were kept in a growth chamber controlled at a 16-h light/8-h dark cycle, 25°C/18°C day/night temperatures. Sterilized Hoagland solution was provided as a nutrient supplement. Plants were harvested after 3 weeks, and the roots were washed thoroughly in tap water. The shoot, root length, and fresh weight of the plants were recorded. Plant samples were dried at 60°C in an oven, and then the dry root and shoot masses were recorded.

### Microscopic examination of the rhizoplane of rice seedlings with SEM

The roots of 1-week-old seedlings were rinsed with distilled water for 30 s in a laminar flow cabinet. Specimens were prepared for the examination under a scanning electron microscope (SEM) as follows. Immediately after rinsing, seminal roots were cut into several segments of 3–5 mm lengths and were then fixed with 2.5% glutaraldehyde dissolved in 0.2 M of sodium phosphate buffer (pH 7.2) for 12 h at 4°C. The segments were subsequently dehydrated using a series of ethanol (30%, 50%, 75%, and 96%) solutions, each for 15 min. Following dehydration, the samples were dried using a Hitachi Critical Point Dryer HCP-I with liquid carbon dioxide and were then coated with gold using a Giko IB-3 Ion Coater. The prepared samples were examined under a Hitachi SEM (SSM-2A) at an accelerating voltage of 10 kV (Asanuma et al., 1979).

### Scoring of parameters

In order to evaluate the effectiveness of bacterial treatments in comparison with the control, parameter scoring was performed. The corresponding equations were used to calculate the actual scores of individual parameters for each treatment (**Table 2**). Scores for every single trait were considered to be zero in the control, and maximum scores were given to the highest value within each trait.

**Table 2:** Scoring system and corresponding equations employed to evaluate different aspects of germination and growth.

Growth traits (21-day-old seedlings)	Maximum scores allocated (MSA)	Germination traits	Maximum scores allocated (MSA)	Score Calculations
PFW (mg)	15	Average no. of roots	10	Trait factor (TF) = MSA/(Maximum value − Control value) Obtained scores = (treatment − control) × TF Total obtained scores = Sum of obtained scores from all traits
SH (cm)	15	ESH (cm)	10	
RL (cm)	15	ERL (cm)	10	
DSM (mg)	20	ESM (mg)	10	
DRM (mg)	20	ERM (mg)	10	
RS ratio	15	SVI1	10	
		SVI2	10	
		GE	20	
		ERS ratio	10	
Total scores	100	Total scores	100	

Avg. no. of roots, average number of roots; ESH, emerging shoot height; ERL, emerging root length; ESM, emerging shoot mass, Emerging root mass (ERM); SVI1, seedling vigor index 1; SVI2, seedling vigor index 2; GE, germination energy on the second day; ERS ratio, emerging root shoot ratio; PFW, plant fresh weight; SH, shoot height; RL, root length; DSM, dry shoot mass; DRM, dry root mass; RS, root shoot ratio.

### PCR amplification, molecular identification, and phylogenetic analysis

Of the 41 selected isolates, 14 bacterial isolates were found to be effective for both biopriming and root inoculation of rice along with their diverse PGP traits. These isolates were identified through 16S rDNA sequencing. Briefly, universal bacterial primers were used in colony PCR to amplify the 16S rDNA sequence. The forward primer F (5′-AGAGTTTGATCMTGGCTCAG-3′) and the reverse primer R (5′-TACGGYTACCTTGTTACGACTT-3′) positioned at 8–27 and 1492–1507, respectively, on the 16S rDNA sequence of *Escherichia coli* were designed. The reaction mixture for PCR had a total volume of 50 μL, including 1 μL of DNA template, 5 μL of 10× reaction buffer, 2.5 μL each of 10 μM F and R primers, 0.5 μL of Dream Taq DNA Polymerase (Thermo Fisher Scientific, Foster City, California, US), and 4 μL of dNTP mixture. The PCR program was as follows: 94°C initial denaturation for 2 min, 94°C denaturation for 30 s, 55°C annealing for 1 min 15 s, 72°C extension for 1 min 30 s, 34 cycles, and 72°C final extension for 5 min. The PCR fragments were analyzed using gel electrophoresis with 1% (w/v) agarose gel. Bands corresponding to the 16S rRNA gene were confirmed, and the PCR fragments were sent to Macrogen Europe for sequencing. The 16S rDNA sequences were obtained and submitted to the GenBank database using the BLAST software to get accession numbers. Sequences homologous to bacterial isolates were compared in the EzBioCloud database, and alignments were performed using the Clustal-W program. The neighbor-joining method was used to construct phylogenetic trees, and evolutionary analysis was performed using the MEGA X software (**Supplementary Figures 1S–4S**).

### Statistical analysis

All the experiments were repeated in triplicate, and data were analyzed using GraphPad Prism 8, IBM SPSS Statistics 27, and Origin 2024. The comparison between treatment means and control was made using Dunnett’s test at *P<*0.05. Principal component analysis (PCA) and hierarchical cluster analysis were performed using the Origin 2024 software.

## Results

### Diversity and characteristics of bacterial colony morphology

We examined bacterial isolates obtained from the rhizosphere soils of eight different trees to assess their colony morphology (**Table 3**). The analysis of the colony form revealed that isolates from *A. nordmanniana* exhibited circular (84.2%) and filamentous (15.8%) colonies. All of the isolates from the rhizospheres of *C. avellana* and *F. carica* were circular in form. Regarding *P. elaeagnifolia*, isolates formed circular (79.3%) and irregular (20.7%) colonies. For *R. pseudoacacia*, isolates showed an almost similar trend with circular (78.6%) and irregular (21.4%) colony forms. Isolates from *J. regia* exhibited circular (50%) and irregular (50%) colonies. For *A. pseudoplatanus* sp., isolates were circular (70%) and irregular (30%) in form. Parallel to this, isolates from *M. domestica* formed circular (64.3%) and irregular (35.7%) colonies.

**Table 3:** Summary of morphological features of bacterial isolates obtained from eight different rhizospheres.

Colony morphology		Rhizospheric isolates							
		*Abies nordmanniana*	*Corylus avellana*	*Pyrus elaeagnifolia*	*Acer pseudoplatanus*	*Robinia pseudoacacia*	*Juglans regia*	*Malus domestica*	*Ficus carica*
Form	Circular	84.2%	100%	79.3%	70%	78.6%	50%	64.3%	100%
	Filamentous	15.8%	0%	0%	0%	0%	0%	0%	0%
	Irregular	0%	0%	20.7%	30%	21.4%	50%	35.7%	0%
Color	Whitish	31.6%	58.3%	58.6%	70%	57.1%	0%	64.3%	28.6%
	Yellowish	52.6%	0%	0%	0%	0%	50%	0%	28.6%
	Creamy	15.8%	41.7%	41.4%	15%	42.9%	50%	35.7%	28.6%
	Transparent	0%	0%	0%	15%	0%	0%	0%	0%
Elevation	Convex	68.4%	0%	0%	0%	0%	0%	0%	0%
	Flat	15.8%	0%	10.3%	15%	0%	0%	0%	0%
	Raised	15.8%	58.3%	69%	45%	21.4%	100%	64.3%	100%
	Umbonate	0%	16.7%	20.7%	45%	78.6%	0%	35.7%	0%
	Crateriform	0%	16.7%	0%	0%	0%	0%	0%	0%
Margin	Entire	68.4%	83.3%	69%	55%	78.6%	100%	64.3%	100%
	Filiform	15.8%	0%	0%	0%	0%	0%	0%	0%
	Undulate	0%	16.7%	10.3%	15%	21.4%	0%	0%	0%
	Curled	15.8%	0%	20.7%	30%	0%	0%	35.7%	0%

In terms of colony color, *A. nordmanniana* isolates displayed 31.6% whitish, 52.6% yellowish, and 15.8% creamy colonies. The isolates from *C. avellana*, *P. elaeagnifolia*, and *R. pseudoacacia* predominantly formed whitish (58.3%) and creamy (41.7%) colonies. *Acer pseudoplatanus* rhizospheric isolates produced whitish (70%), creamy (15%), and transparent (15%) colonies. *Malus domestica* isolates were 64.3% whitish and 35.7% creamy, whereas *F. carica* isolates had a more diverse color distribution, showing 28.6% each of whitish, yellow, and creamy colonies.

In terms of colony elevation, isolates from *A. nordmanniana* exhibited convex (68.4%), flat (15.8%), or raised (15.8%) elevations. All isolates from *J. regia* exhibited raised elevations, and 45% of *A. pseudoplatanus* isolates displayed raised or umbonate elevations, while 15% showed flat elevations. In contrast, 78.6% of *R. pseudoacacia* isolates exhibited umbonate elevations, with the remaining (21.4%) showing raised elevations. *Corylus avellana* isolates displayed 16.4% umbonate or crateriform, while the rest (58.3%) exhibited raised elevations.

Regarding colony margins, 68.4% of isolates from *A. nordmanniana* exhibited entire margins, while the remaining isolates had either filiform (15.8%) or curled (15.8%) margins. The majority of isolates from *C. avellana* (83.3%) and *M. domestica* (64.3%) also exhibited entire margins. *Acer pseudoplatanus* isolates showed entire (55%), undulate (15%), and curled margins (30%), while isolates from *M. domestica* had entire (78.6%) and curled (21.4%) margins. All the isolates from *F. carica* exhibited entire margins.

Overall, the variations in colony morphology such as form, color, elevation, and margins came from differences in genetic makeup, environmental adaptability, and interactions with the host plant.

### Indole-3-acetic acid production profiles of rhizospheric bacterial isolates

We examined a total of 129 bacteria isolated from different rhizospheres for their IAA production. The findings are detailed in **Tables 4**, **5**. The results indicated that a significant majority, 109 isolates, demonstrated the ability to synthesize IAA, highlighting the widespread potential of these bacteria to influence plant growth through hormonal interactions.

From the *A. nordmanniana* rhizosphere, isolate R1(B) stood out with the highest IAA production at 353.5 ± 5.7 µg/mL followed by S1(E) at 180 ± 14.5 µg/mL, S1(B) at 144.5 ± 6.8 µg/mL, SS1(1) at 136 ± 57.2 µg/mL, SS1(4) at 121.4 ± 12.4 µg/mL, and S1(G) at 107.9 ± 3.1 µg/mL. All 19 bacterial isolates derived from this rhizosphere were able to produce IAA. From the *C. avellana* rhizosphere, all 12 bacterial isolates produced IAA. Particularly, isolate SS2(4) recorded a high IAA production level of 353.5 ± 95 µg/mL followed by SS2(1)2 with 259.3 ± 32 µg/mL, L2(6)2 with 169.6 ± 93.6 µg/mL, R2(2)2 with 135.6 ± 119.6 µg/mL, and SS2(4)2 with 59.4 ± 1.2 µg/mL. For *P. elaeagnifolia*, 90% of the 29 isolates were capable of producing IAA. The highest production of 511.9 ± 133.8 µg/mL was observed in isolate SS3(8) followed by SS3(5)1 with 447 ± 11.7 µg/mL, SS3(6)1 with 403 ± 23 µg/mL, SS3(9) with 256 ± 28.5 µg/mL, S3(1) with 224.5 ± 23.2 µg/mL, S3(3) with 185.6 ± 7.7 µg/mL, SS3(5)2 with 130 ± 26.5 µg/mL, SS3(2)2 with 121.2 ± 50.5 µg/mL, SS3(2)1 with 104.7 ± 44.4 µg/mL, and SS3(4)1 with 84.2 ± 37.6 µg/mL, indicating a varying potential of bacteria to modulate plant growth through hormonal interaction. The *A. pseudoplatanus* rhizosphere showed that 95% of its 20 bacterial isolates were capable of synthesizing IAA. R4(1)2 exhibited the highest IAA production at 739.9 ± 251.5 µg/mL, followed by SS4(10) at 183.5 ± 10.5 µg/mL, SS4(8) at 170.3 ± 89.8 µg/mL, SS4(5) at 146.3 ± 72.1 µg/mL, S4(2) at 116.7 ± 14.2 µg/mL, L4(1) at 102.7 ± 11.1 µg/mL, and SS4(7) at 53 ± 4.4 µg/mL. From *R. pseudoacacia*, 71% of the 14 isolates produced IAA, with R5(1) showing the maximum production of 148.6 ± 12.2 µg/mL followed by S5(1) with 91 ± 64.7 µg/mL, SS5(5) with 73.8 ± 12.1 µg/mL, R5(1)1 with 39.4 ± 0.9 µg/mL, and S5(2)1 with 28.9 ± 2 µg/mL. *Juglans regia* and *M. domestica* both showed that 57% of their 14 isolates produced IAA. Notably, R6(3) from *J. regia* showed a peak production of 125.5 ± 4.2 µg/mL followed by SS6 (5) with 117.5 ± 2.8 µg/mL. Isolate R7(3) from *M. domestica* exhibited the highest production of 122.1 ± 35.2 µg/mL followed by R7(1) with 121.1 ± 3.1 µg/mL and SS7(5) with 85 ± 15 µg/mL. *Ficus carica* had 86% of its 7 isolates producing IAA, with isolate S8 producing the highest at 108.3 ± 8.6 µg/mL followed by SS8(6) with 84.5 ± 12.1 µg/mL and SS8(2) with 32.4 ± 4.6 µg/mL.

These results highlight the significant phytohormonal influence these isolates may have on their host plants. These bacterial isolates are potential growth stimulators to enhance the growth of plant through natural hormonal supplementation. The variability in IAA production of different isolates represented the diversity in the metabolic capabilities of rhizosphere-associated bacteria.

### Assessment of siderophore production

In examining siderophore production among bacterial isolates derived from various tree rhizospheres, only a small fraction of the 129 tested isolates demonstrated this capability, with just 16 isolates showing positive results. The results, which are detailed in **Tables 4**, **5**, highlighted significant variability in siderophore production across different rhizospheres.

Among the isolates from *C. avellana*, 17% were capable of producing siderophores, with isolate L2 (6)2, showing notably high production at 62.2% ± 33.4% followed by R2(2)2 at 14.5% ± 4.3%. In contrast, only 10% of the bacterial isolates from the rhizosphere of *P. elaeagnifolia* were able to produce siderophores, with the peak production recorded at 22.2% ± 5.2% by isolate SS3(2)1 followed by SS3(9) at 12.9% ± 7.5%, and SS3(2)2 at 3.8% ± 1.7%. A slightly higher proportion of *A. pseudoplatanus*-associated isolates, 20%, produced siderophores, with the highest level observed in isolate L4 (1) at 26.5% ± 1.3%, SS4(10) at 15.8% ± 5%, SS4(8) at 9.3% ± 3.9%, and S4(2) at 3.7% ± 1.8%.

Isolates from the rhizosphere of *R. pseudoacacia* showed a relatively better capability, with 28% isolates producing siderophores. Among these isolates, R5(1) recorded the highest production rate at 91.1% ± 0.8% followed by SS5(5) at 38.7% ± 10.1%, S5(1) at 37.4% ± 1%, and S5(2)1 at 2.5% ± 0.7%. Both *M. domestica* and *F. carica* had 14% of their isolates capable of producing siderophores, with the highest outputs being 9.4% ± 1.1% in isolate R7(1) from *M. domestica* followed by R7(3) at 8.9% ± 1.8%. Isolate S8 showed peak production of siderophores at 50.1% ± 2.6% from the *F. carica* rhizosphere.

Interestingly, none of the isolates from *A. nordmanniana* and *J. regia* exhibited siderophore production. This distinct absence highlights the adaptive traits of microbial communities associated with a specific rhizosphere. The ability to produce siderophores helps in iron chelation which enhances plant growth by influencing the dynamics of nutrient uptake within these rhizospheric ecosystems.

### Inorganic phosphate solubilization activity

The inorganic phosphate solubilization activity of 129 bacterial isolates was assessed using a plate assay. A total of nine isolates exhibited clear zones around bacterial growth, indicating phosphate solubilization (**Figure 3C**, **Tables 4**, **5**). Approximately 8.3% of the isolates from the rhizosphere of *C. avellana* showed solubilization activity, with isolate SS2(4)2 forming a clear zone of 2.6 ± 0.3. In the rhizosphere of *P. elaeagnifolia*, 6.9% of the isolates demonstrated phosphate solubilization, with the highest activity recorded in isolate SS3(4)1 at 2.9 ± 0.3 followed by SS3(6)1 at 2.7 ± 0.2.

Among the isolates from the rhizosphere of *A. pseudoplatanus* sp., 5% exhibited phosphate solubilization, with the maximum zone size observed in isolate SS4(7) at 3.1 ± 0.3. From the rhizosphere of *R. pseudoacacia*, 21.4% (highest frequency) of the isolates were positive for phosphate solubilization, with isolate S5(2)1 showing the peak zone size of 3.8 ± 0.5 followed by S5(1) at 3.1 ± 0.5 and R5(1)1 at 2.8 ± 0.3.

From the rhizosphere soil of *M. domestica*, 7.1% of the isolates were capable of forming clear zones, with isolate R7(1) producing a zone of 1.6 ± 0.3. Among the isolates from the rhizosphere of *F. carica*, 14.3% exhibited phosphate solubilization, with isolate SS8(2) forming a zone size of 2.3 ± 0.1.

None of the isolates from the rhizospheres of *A. nordmanniana* and *J. regia* showed phosphate solubilization activity. These results highlight variability in phosphate solubilization among bacteria associated with a particular rhizosphere.

### Identification of potential endogenous nitrogen-fixing diazotrophs of rice among isolated bacteria

Identification of potential endogenous nitrogen-fixing diazotrophs in different parts (leaf, stem, and root) of rice was performed using semisolid NFB media. N-fixing diazotrophic bacteria fix atmospheric nitrogen which was confirmed through a color change in media from green to blue along with clear pellicle formation.

Briefly, of the 129 total isolates derived from the root zones of different trees, 50.4% were able to localize in different organs of rice plant (7.8% in the leaf, 17.1% in the stem, and 25.6% in the root) and were identified as potential endogenous nitrogen-fixing diazotrophs of rice **Tables 4**, **5**). Out of the 19 isolates derived from *A. nordmanniana*, 73.7% showed positive growth on NFB media and were able to reside inside various parts of rice plant (36.8% in the stem including S1(B), S1(E), and S1(G) and 36.8% in the root including R1(B)). Out of the 12 isolates from *C. avellane*, 66.7% showed their potential for nitrogen fixation inside rice plants (16.7% in the leaf including L2(6)2 and 50% in the root including R2(2)2).

Of the 29 isolates from the *P. elaeagnifolia* rhizosphere, 55.2% exhibited their potential for nitrogen fixation inside rice plants (20.7% in the leaf, 10.3% in the stem including S3(1) and S3(3), and 50% in the root). Out of the 29 isolates from the root zone of *A. pseudoplatanus*, 40% revealed their potential for nitrogen fixation inside rice plants (10% in the leaf including L4(1), 15% in the stem including S4 (2), and 15% in the root including R4(1)2). Of the 14 isolates from *R. pseudoacacia*, 50% showed their potential ability for N-fixation inside rice plants (28.6% in the stem including S5(1) and S5(2)1 and 21.4% in the root including R5(1)1).

Out of the 14 isolates from *J. regia*, 35.7% showed their potential for N-fixation inside rice plants [7.1% in the stem and 28.6% in the root including R6(3)]. Of the 14 isolates from *M. domestica*, 42.9% showed their potential for N-fixation inside rice plants [21.4% in the stem and 21.4% in the root including R7(1) and R7(3)]. Out of the 14 isolates from *F. carica*, 14.3% including S8(2) showed their potential ability for N-fixation inside rice plants. This extensive identification and evaluation process underscores the diverse localization potential of forest-isolated diazotrophic microbes inside rice plants.

### Hydrolytic enzymes (protease, cellulase, and amylase) production of rhizosphere-associated bacteria

The ability of bacterial isolates to produce clear zones in skim milk agar plate signified protease activity (**Figure 4A**). The results are detailed in **Tables 4**, **5**. Briefly, 31.6% of the bacteria isolated from the rhizosphere soil of *A. nordmanniana* exhibited protease activity, with the highest activity observed in SS1(1) at 2.9 ± 0.5 followed by SS1(4) at 1.2 ± 0. Similarly, 41.7% of the isolates from *C. avellana* were capable of catalyzing protein, with the highest catalytic activity found in R2(2)2 at 3.5 ± 0.4 followed by SS2(1)2 at 2.3 ± 0.2. Approximately 20.7% of the isolates from *P. elaeagnifolia* showed protease activity, with the highest (3.9 ± 0.4) in SS3(4)1 followed by SS3(5)2 at 3.5 ± 0.4. The most significant incidence (70%) of protease production was noted in isolates from *A. pseudoplatanus*, with the maximum activity measured at 4 ± 0.3 in SS4(7) followed by SS4(10) at 3.8 ± 0.9, R4(1)2 at 1.9 ± 0.3, L4(1) at 1.7 ± 0.2, and SS4(5) at 1.3 ± 0.1. Additionally, 50% of the bacteria derived from the root zone of *J. regia* and 35.7% from *M. domestica* demonstrated protease activity, with a notable activity of 5.5 ± 0.6 in isolate SS6(5) and 1.7 ± 0.3 in isolate R7(3). Almost 71.4% of the isolates from the rhizosphere of *F. carica* were positive for protease production, with the highest activity (2.9 ± 0.1) in isolate SS8(2) followed by SS8(6) at 2.7 ± 0.3. Out of the 41 shortlisted isolates, protease activity was confirmed in 15 bacteria.

Starch digestion was analyzed to assess amylase production (**Tables 4**, **5**, **Figure 4B**). The frequencies of amylase-producing bacteria were noted as 15.8%, 16.7%, 20.7%, and 30% from *A. nordmanniana* (including SS1(1), +), *C. avellana* (including L2(6)2, +), *P. elaeagnifolia* (including SS3(4)1, +; SS3(5), +), and *A. pseudoplatanus* (including SS4(7), +; L4(1), +) as mentioned in **Tables 4**, **5**. Of the 41 selected bacteria, 6 were positive for amylase activity.

Cellulase activity was assessed using a carboxymethylcellulose Congo red media plate (**Tables 4**, **5**, **Figure 4C**). Approximately 52.6% of the isolates from *A. nordmanniana* displayed varying levels of cellulase activity from the highest (+++) in S1(G), moderate (++) in R1(B), to low (+) in SS1(1). Almost 16.7% of the isolates (including SS2(4)2, +++) from *C. avellana* and approximately 10.3% from *P. elaeagnifolia* (including S3(3), +) exhibited cellulase activity. In contrast, 30% of the bacteria (including L4(1), +++; R4(1)2, +++) from *A. pseudoplatanus* and 21.4% from *R. pseudoacacia* (including R5(1), ++) exhibited cellulase activity. Approximately 71.4% of bacteria originating from the rhizosphere soil of *F. carica* (including SS8(6) and S8) showed activity, but in a low range as given in **Table 4**. Of the 41 selected isolates, 10 showed cellulase activity (**Table 5**).

Variations in enzyme production among rhizosphere-associated bacteria highlight their specialized functions to specific rhizospheric environments.

### Hydrogen cyanide production

Hydrogen cyanide (HCN) production was assessed in bacterial isolates, as indicated by the browning of filter paper. Approximately 31.6% of the isolates from the rhizosphere of *A. nordmanniana* were capable of producing HCN, with isolates SS1(4) and S1(G) exhibiting low production levels (+). Approximately 16.7%% of bacteria isolated from the root zone of *C. avellana* produced HCN, with moderate production (++) noted in isolate L2(6)2. For *P. elaeagnifolia*, 31% of the bacterial isolates produced HCN, with isolate SS3(9) showing significant activity (+++), confirmed by a dark brown reaction on the filter paper followed by SS3(2)1 (+) and SS3(2)2 (+), exhibiting low production as given in **Tables 4**, **5**.

**Table 4:** Percentage of bacterial isolates possessing plant growth-promoting (PGP) traits from different rhizospheres.

PGP trait	Rhizospheric isolates							
	*Abies nordmanniana*	*Corylus avellana*	*Pyrus elaeagnifolia*	*Acer pseudoplatanus*	*Robinia pseudoacacia*	*Juglans regia*	*Malus domestica*	*Ficus carica*
Indole acetic acid (IAA) production	100%	100%	89.7%	95%	71.4%	57.1%	57.1%	85.7%
Formation of siderophores	0%	16.7%	10.3%	20%	28.6%	0%	14.3%	14.3%
Phosphate solubilization	0%	8.3%	6.9%	5%	21.4%	0%	7.1%	14.3%
Endophytic N-fixation in rice	73.7%	66.7%	55.2%	40%	50%	35.7%	42.9%	14.3%
Amylase activity	15.8%	16.7%	20.7%	30%	0%	0%	0%	0%
Protease activity	31.6%	41.7%	20.7%	70%	0%	50%	35.7%	71.4%
Cellulase activity	52.6%	16.7%	10.3%	30%	21.4%	0%	0%	71.4%
HCN production	31.6%	16.7%	31%	45%	21.4%	50%	0%	0%
Ammonia production	31.6%	41.7%	58.6%	0%	21.4%	0%	0%	71.4%
3% NaCl tolerance	84.2%	58.3%	100%	100%	100%	50%	100%	100%
5% NaCl tolerance	15.8%	41.7%	69%	70%	100%	50%	64.3%	71.4%
7% NaCl tolerance	15.8%	16.7%	41.4%	70%	100%	50%	64.3%	71.4%

From *A. pseudoplatanus*, 45% of the isolates (including SS4(5), +; L4(1), +; and R4(1)2, +) produced HCN. Among the isolates from *R. pseudoacacia* and *J. regia*, 21.4% and 50%, respectively, showed HCN production (including R5(1), +and R6(3), +). No HCN production was detected in the isolates from *M. domestica* or *F. carica*. Among the 41 selected isolates, 11 were tested positive for HCN (**Table 5**).

These results underscore the potential of these bacteria for biological control applications, given the inhibitory effects of HCN on plant pathogens.

### Ammonia producers across different rhizospheres

Ammonia production was detected through a color change in peptone water from yellow to brown. The frequencies of ammonia producers varied among isolates derived from different rhizosphere soils as detailed in **Tables 4**, **5**. Specifically, 31.6% of the bacterial isolates [including S1(B) and S1(G)] from the rhizosphere of *A. nordmanniana* produced ammonia. Similarly, 41.7% of the isolates [including SS2(1)2 and R2(2)2) from *C. avellana*, 58.6% from *P. elaeagnifolia* (including SS3(4)1, SS3(5)2, SS3(6)1, and SS3(8)], and 71.4% from *F. carica* (including SS8(6) and S8) exhibited ammonia production capabilities. In contrast, only 21.4% of the isolates [including R5(1)] from *R. pseudoacacia* were capable of producing ammonia, while no ammonia-producing bacteria were found among the isolates from *A. pseudoplatanus*, *J. regia*, and *M. domestica.* Ammonia production was confirmed in 13 out of 41 selected bacterial isolates (**Table 5**).

**Table 5:** Plant growth-promoting (PGP) traits of selected bacterial isolates originating from different rhizospheres.

Rhizosphere soil	Isolates	Source of isolation	IAA	SDPs	PS	Endophytic N-fixation in rice	Amylase activity	Protease activity	Cellulase activity	HCN	NH_3_
*Abies nordmanniana*	SS1(1)	Soil	136 ± 57.2	−	−	−	+	2.9 ± 0.5	+	−	−
	SS1(4)		121.4 ± 12.4	−	−	−	−	1.2 ± 0	−	+	−
	S1(B)	Rice stem	144.5 ± 6.8	−	−	+	−	−	−	−	+
	S1(E)		180 ± 14.5	−	−	+	−	−	−	−	−
	S1(G)		107.9 ± 3.1	−	−	+	−	−	+++	+	+
	R1(B)	Rice root	353.5 ± 5.7	−	−	+	−	−	++	−	−
*Corylus avellana*	SS2(1)2	Soil	259.3 ± 32	−	−	−	−	2.3 ± 0.2	−	−	+
	SS2(4)		353.5 ± 95	−	−	−	−	−	−	−	−
	SS2(4)2		59.4 ± 1.2	−	2.6 ± 0.3	−	−	−	+++	−	−
	L2(6)2	Rice leaf	169.6 ± 93.6	62.2 ± 33.4	−	+	+	−	−	++	−
	R2(2)2	Rice root	135.6 ± 119.6	14.5 ± 4.3	−	+	−	3.5 ± 0.4	−	−	+
*Pyrus elaeagnifolia*	SS3(2)1	Soil	104.7 ± 44.4	22.2 ± 5.2	−	−	−	−	−	+	−
	SS3(2)2		121.2 ± 50.5	3.8 ± 1.7	−	−	−	−	−	+	−
	SS3(4)1		84.2 ± 37.6	−	2.9 ± 0.3	−	+	3.9 ± 0.4	−	−	+
	SS3(5)1		447 ± 11.7	−	−	−	−	−	−	−	−
	SS3(5)2		130 ± 26.5	−	−	−	+	3.5 ± 0.4	−	−	+
	SS3(6)1		403 ± 23	−	2.7 ± 0.2	−	−	−	−	−	+
	SS3(8)		511.9 ± 133.8	−	−	−	−	−	−	−	+
	SS3(9)		256 ± 28.5	12.9 ± 7.5	−	−	−	−	−	+++	−
	S3(1)	Rice stem	224.5 ± 23.2	−	−	+	−	−	−	−	+
	S3(3)		185.6 ± 7.7	−	−	+	−	−	+	−	+
*Acer pseudoplatanus*	SS4(5)	Soil	146.3 ± 72.1	−	−	−	−	1.3 ± 0.1	−	+	−
	SS4(7)		53 ± 4.4	−	3.1 ± 0.3	−	+	4 ± 0.3	−	−	−
	SS4(8)		170.3 ± 89.8	9.3 ± 3.9	−	−	−	−	−	−	−
	SS4(10)		183.5 ± 10.5	15.8 ± 5	−	−	−	3.8 ± 0.9	−	−	−
	L4(1)	Rice leaf	102.7 ± 11.1	26.5 ± 1.3	−	+	+	1.7 ± 0.2	+++	+	−
	S4(2)	Rice stem	116.7 ± 14.2	3.7 ± 1.8	−	+	−	−	−	−	−
	R4(1)2	Rice root	739.9 ± 251.5	−	−	+	–	1.9 ± 0.3	+++	+	–
*Robinia pseudoacacia*	SS5(5)	Soil	73.8 ± 12.1	38.7 ± 10.1	−	−	−	−	−	−	−
	S5(1)	Rice stem	91 ± 64.7	37.4 ± 1	3.1 ± 0.5	+	−	−	−	−	−
	S5(2)1		28.9 ± 2	2.5 ± 0.7	3.8 ± 0.5	+	−	−	−	−	−
	R5(1)	Rice root	148.6 ± 12.2	91.1 ± 0.8	−	+	−	−	++	+	+
	R5(1)1		39.4 ± 0.9	−	2.8 ± 0.3	+	−	−	−	−	−
*Juglans regia*	SS6(5)	Soil	117.5 ± 2.8	−	−	−	−	5.5 ± 0.6	−	−	−
	R6(3)	Rice root	125.5 ± 4.2	−	−	+	−	−	−	++	−
*Malus domestica*	SS7(5)	Soil	85 ± 15	−	−	−	−	−	−	−	−
	R7(1)	Rice root	121.1 ± 3.1	9.4 ± 1.1	1.6 ± 0.3	+	−	−	−	−	−
	R7(3)		122.1 ± 35.2	8.9 ± 1.8	−	+	−	1.7 ± 0.3	−	−	−
*Ficus carica*	SS8(2)	Soil	32.4 ± 4.6	−	2.3 ± 0.1	−	−	2.9 ± 0.1	−	−	−
	SS8(6)		84.5 ± 12.1	−	−	−	−	2.7 ± 0.3	+	−	+
	S8	Rice stem	108.3 ± 8.6	50.1 ± 2.6	−	+	−	−	+	−	+

Values are mean ± standard deviation. +++ = high, ++ = moderate, and low = +.

IAA, indole acetic acid (µg/mL); SDPs, percent siderophores; PS, phosphate solubilization; NH_3_, ammonia production; HCN, hydrogen cyanide production.

Particularly, the rhizosphere of *P. elaeagnifolia* and *F. carica* showed higher frequencies of ammonia producers, suggesting their potential role in nitrogen cycling within their respective ecosystems.

### Salt tolerance of bacterial isolates at different NaCl concentrations

The study of bacterial isolates revealed a continuous decline in growth with increasing concentrations of NaCl. Approximately 84.2% of the isolates [including SS1(1), SS1(4), S1(B), S1 (G), R1(B)] from *A. nordmanniana* were able to survived at 3% NaCl concentration. Isolate S1(G) notably withstood NaCl up to 7% concentration. Similarly, 58.3% of the isolates (including SS2(4)2, L2(6)2, and R2(2)2) from *C. avellana* tolerated 3% NaCl. Particularly, isolate L2(6)2 grew up to 7% NaCl (detailed in **Table 6**).

All the isolates from *P. elaeagnifolia* survived 3% NaCl, 69% isolates (including SS3(2)1, SS3(2)2, SS3(4)1, SS3(5)1, and SS3(5)2) survived 5% NaCl, and 41.4% isolates [including SS3(4)1, SS3(5)2, S(1), and S3(3)] tolerated as high as 7% NaCl. For *A. pseudoplatanus*, all the isolates grew at 3% NaCl, with 70% (including SS4(7), SS4(10), L4(1), S4(2), and R4(1)2) surviving up to 7% NaCl (detailed in **Table 6**). From *R. pseudoacacia*, all the bacteria were viable at 3%, 5%, and 7% NaCl.

Approximately 50% of the isolates from *J. regia* tolerated NaCl up to 7%. All the isolates from *M. domestica* tolerated 3% NaCl, though 64.3% managed to survive 7% NaCl including R7(1) and R7(3). Every isolate from *F. carica* survived at 3% NaCl, but 71.4% of the isolates including SS8(2) and SS8 (6) tolerated up to 7% NaCl concentration (detailed in **Table 6**).

**Table 6:** Growth of selected bacterial isolates at different salt concentrations and different time intervals.

Rhizosphere soil	Isolate	Source of isolation	3% NaCl 24 h	3% NaCl 48 h	3% NaCl 72 h	5% NaCl 24 h	5% NaCl 48 h	5% NaCl 72 h	7% NaCl 24 h	7% NaCl 48 h	7% NaCl 72 h
*Abies nordmanniana*	SS1(1)	Soil	−	+	+	−	−	−	−	−	−
	SS1(4)		−	+	+	−	−	−	−	−	−
	S1(B)	Rice stem	−	+	+	−	−	−	−	−	−
	S1(E)		−	−	−	−	−	−	−	−	−
	S1(G)		+	+	+	+	+	+	−	+	+
	R1(B)	Rice root	−	−	+	−	−	−	−	−	−
*Corylus avellana*	SS2(1)2	Soil	−	−	−	−	−	−	−	−	−
	SS2(4)		−	−	−	−	−	−	−	−	−
	SS2(4)2		+	+	+	−	+	+	−	−	−
	L2(6)2	Rice leaf	+	+	+	+	+	+	+	+	+
	R2(2)2	Rice root	+	+	+	−	−	−	−	−	−
*Pyrus elaeagnifolia*	SS3(2)1	Soil	+	+	+	−	−	+	−	−	−
	SS3(2)2		+	+	+	−	+	+	−	−	−
	SS3(4)1		+	+	+	−	+	+	−	−	+
	SS3(5)1		+	+	+	−	+	+	−	−	−
	SS3(5)2		+	+	+	+	+	+	−	+	+
	SS3(6)1		+	+	+	−	−	−	−	−	−
	SS3(8)		+	+	+	−	−	−	−	−	−
	SS3(9)		+	+	+	−	−	−	−	−	−
	S3(1)	Rice stem	+	+	+	+	+	+	−	−	+
	S3(3)		+	+	+	+	+	+	+	+	+
*Acer pseudoplatanus*	SS4(5)	Soil	+	+	+	−	−	−	−	−	−
	SS4(7)		+	+	+	−	+	+	+	+	+
	SS4(8)		+	+	+	−	−	−	−	−	−
	SS4(10)		+	+	+	−	+	+	−	+	+
	L4(1)	Rice leaf	+	+	+	+	+	+	+	+	+
	S4(2)	Rice stem	+	+	+	+	+	+	+	+	+
	R4(1)2	Rice root	+	+	+	+	+	+	+	+	+
*Robinia pseudoacacia*	SS5(5)	Soil	+	+	+	+	+	+	+	+	+
	S5(1)	Rice stem	+	+	+	–	+	+	−	+	+
	S5(2)1		+	+	+	+	+	+	+	+	+
	R5(1)	Rice root	+	+	+	+	+	+	+	+	+
	R5(1)1		+	+	+	+	+	+	+	+	+
*Juglans regia*	SS6(5)	Soil	+	+	+	+	+	+	+	+	+
	R6(3)	Rice root	−	−	−	−	−	−	−	−	−
*Malus domestica*	SS7(5)	Soil	+	+	+	−	−	−	−	−	−
	R7(1)	Rice root	+	+	+	+	+	+	+	+	+
	R7(3)		+	+	+	−	−	+	−	−	+
*Ficus carica*	SS8(2)	Soil	+	+	+	+	+	+	+	+	+
	SS8(6)		+	+	+	−	+	+	−	+	+
	S8	Rice stem	+	+	+	−	−	−	−	−	−

+, positive growth; −, no growth; NaCl, sodium chloride; 24 h, 24-h interval; 48 h, 48-h interval; 72 h, 72-h interval.

The bacteria associated with the *R. pseudoacacia* rhizosphere exhibited the highest tolerance at 7% NaCl concentration as shown in **Table 6** with 100% survival rate. These results highlighted the potential adaptations and tolerance of the microbial community associated with the rhizosphere of *R. pseudoacacia* under high salt conditions.

Rhizospheric bacteria associated with *R. pseudoacacia*, *A. pseudoplatanus*, and *F. carica* were highly tolerant; however, *J. regia*- and *M. domestica-*associated bacteria exhibited moderate tolerance, while *P. elaeagnifolia-*, *C. avellane-*, and *A. nordmanniana*-associated bacteria displayed the least tolerance, respectively, when provided with high NaCl concentrations.

### Effect of seed biopriming with bacterial isolates on the germination of rice

Based on significant PGP traits, 41 bacterial isolates out of 129 were selected and tested in the germination trial in order to check their effectiveness as rice seed biopriming agents.

No significant change in final germination percentage was recorded with the application of bacterial isolates as compared to the control; however, germination traits such as emerging shoot height (ESH), ERL, ESM, ERM, SVI, SVI2, GE, emerging root shoot (ERS) ratio, and Avg. no. of roots exhibited significant difference from the control as detailed in **Table 7**.

**Figure 5:**
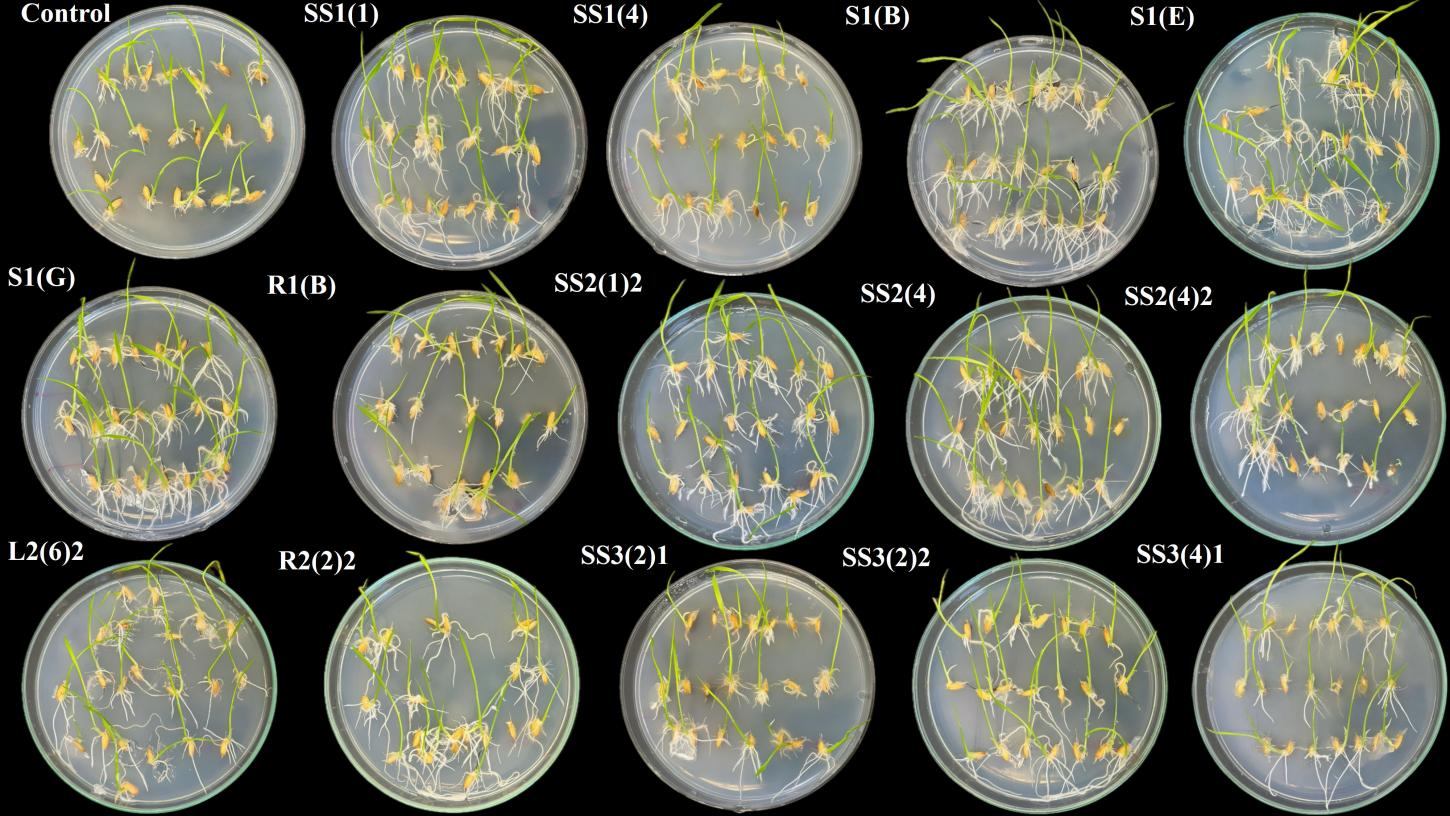
Impact of seed biopriming with selected plant growth-promoting bacteria isolated from rhizosphere soils of *Abies nordmanniana, Corylus avellane*, and *Pyrus elaeagnifolia* on the early growth of emerging rice seedlings in comparison to control.

Isolate S1(E) derived from *A. nordmanniana* exhibited superior performance as a biopriming agent (see **Figure 5**) with the highest score of 81.6 followed by SS1(4) with 48.3, SS1(1) with 46.6, S1(G) with 39, S1(B) with 38.3, and R1(B) with 26.5 as shown in **Table 7**. Application of bacterial isolates increased the germination energy (GE) (S1(E), 53.8%; SS1(4), 30.9%; SS1(1), 7.8%; S1(G), 30.9; S1(B), 30.9%; and R1(B), 30.9%), SVI1 (S1(E), 190.5%; SS1(4), 101.1%; SS1(1), 117%; S1(G), 56.1%; S1(B), 64.4%; and R1(B), 49%), SVI2 (S1(E), 114.2%; SS1(4), 66.3%; SS1(1), 80%; S1(G), 42.1%; S1(B), 45%; and R1(B), 22%), average number of roots (S1(E), 12.8%; SS1(4), 21.3%; SS1(1), 27.7%; S1(G), 27.7%; S1(B), 27.7%; and R1(B), 0%), ESH (S1(E), 43.9%; SS1(4), 29.3%; SS1(1), 36.6%; S1(G), 36.6%; S1(B), 22%; and R1(B), 9.8%), ERL (S1(E), 1214.3%; SS1(4), 585.7%; SS1(1), 642.9%; S1(G), 357.1%; S1(B), 485.7%; and R1(B), 300%), ESM (S1(E), 40.6%; SS1(4), 31.3%; SS1(1), 43.8%; S1(G), 34.4%; S1(B), 25%; and R1(B), 0%), ERM (S1(E), 193.5%; SS1(4), 103.2%; SS1(1), 119.4%; S1(G), 64.5%; S1(B), 66.7%; and R1(B), 61.3%), and ERS ratio (S1(E), 87.5%; SS1(4), 50%; SS1(1), 50%; S1(G), 12.5%; S1(B), 25%; and R1(B), 37.5%) with respect to control. All the selected isolates from *A. nordmanniana* showed overall positive impact on germination.

Among the bacterial isolates obtained from the rhizosphere of *C. avellane*, isolate R2(2)2 got the highest score of 62.1 followed by SS2(1)2 with 47.2, SS2(4) with 37.8, L2(6)2 with 36.4, and SS2(4)2 with 28.3 as mentioned in **Table 7**. Treatment with bacterial isolates increased the different germination parameters such as germination energy (R2(2)2, 46.2%; SS2(1)2, 15.3%; SS2(4), 23.1%; L2(6)2, 38.4%; and SS2(4)2, 15.3%), seedling vigor index 1 (R2(2)2, 101.1%; SS2(1)2, 115.8%; SS2(4), 55%; L2(6)2, 49%; and SS2(4)2, 40.1%), seedling vigor index 2 (R2(2)2, 74.2%; SS2(1)2, 69.7%; SS2(4), 31.7%; L2(6)2, 37.4%; and SS2(4)2, 23.7%), average number of roots (R2(2)2, 21.3%; SS2(1)2, 27.7%; SS2(4), 97.9%; L2(6)2, 6.4%; and SS2(4)2, 42.6%), emerging shoot height (R2(2)2, 58.5%; SS2(1)2, 43.9%; SS2(4), 22%; L2(6)2, 29.3%; and SS2(4)2, 29.3%), emerging root length (R2(2)2, 900%; SS2(1)2, 471.4%; SS2(4), 271.4%; L2(6)2, 442.9%; and SS2(4)2, 328.6%), emerging shoot mass (R2(2)2, 46.9%; SS2(1)2, 31.3%; SS2(4), 15.6%; L2(6)2, 25%; and SS2(4)2, 18.8%), emerging root mass (R2(2)2, 103.2%; SS2(1)2, 129%; SS2(4), 64.5%; L2(6)2, 51.6%; and SS2(4)2, 54.8%), and emerging root shoot ratio (R2(2)2, 25%; SS2(1)2, 50%; SS2(4), 25%; L2(6)2, 12.5%; and SS2(4)2, 12.5%) as compared to control. All the selected isolates from *C. avellana* presented overall positive affect as biopriming agents on the germination traits of rice (see **Figure 5**).

**Figure 6:**
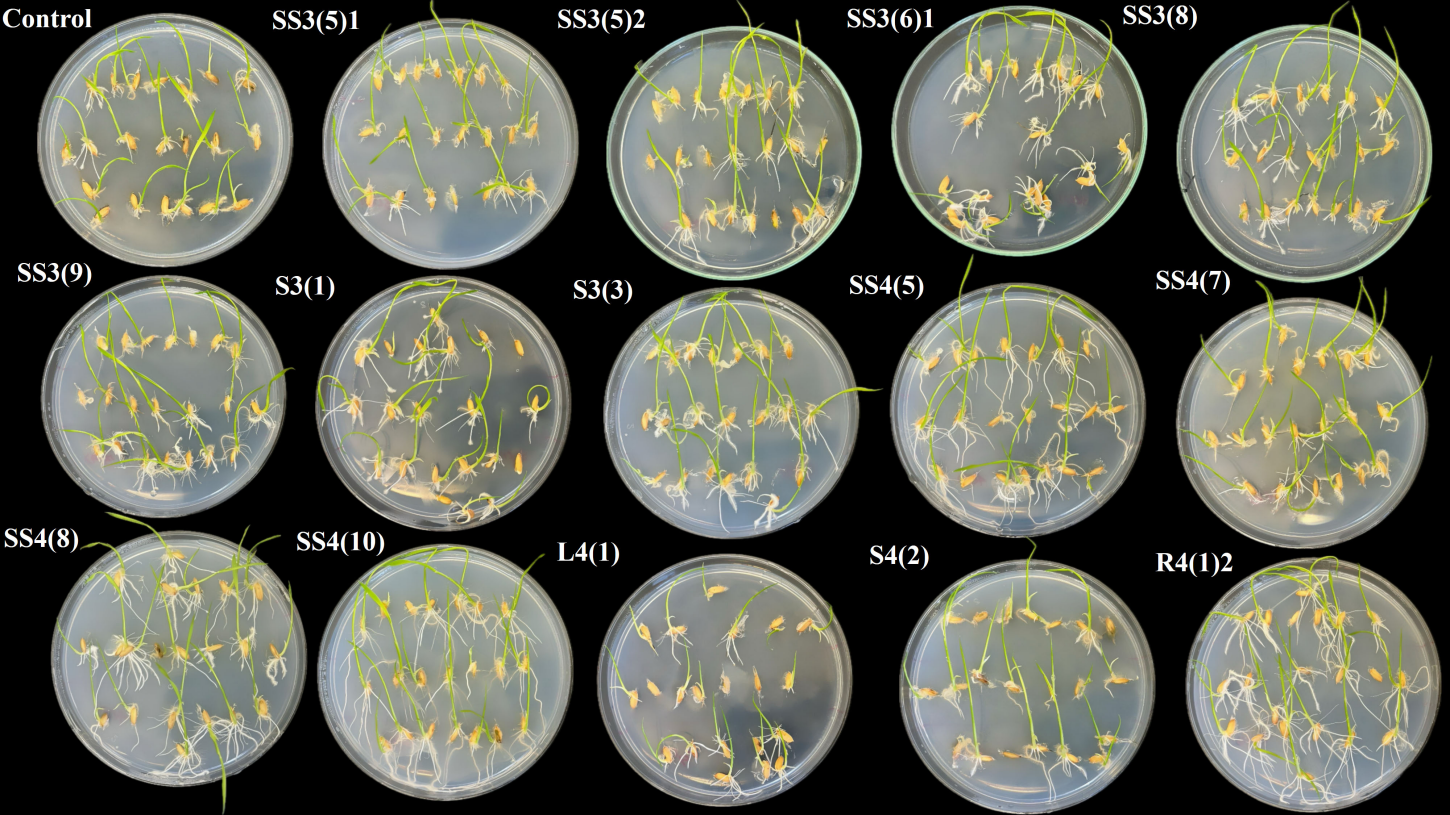
Impact of seed biopriming with selected plant growth-promoting bacteria isolated from rhizosphere soils of *Pyrus elaeagnifolia* and *Acer pseudoplatanus* on the early growth of emerging rice seedlings in comparison to control.

The application of selective bacterial isolates derived from the root zone of *P. elaeagnifolia* showed overall positive results on germination parameters (see **Figures 5**, **6**) except a few isolates like SS3(5)1, SS3(4)1, and S3(3) showing little, no, or negative impact on some of the germination parameters as mentioned in **Table 7**. The highest score of 54.8 was achieved by the isolate SS3(2)2 followed by SS3(6)1 with 35, SS3(8) with 26.1, S3(1) with 25.7, SS3(9) with 23.9, SS3(2)1 with 20, SS3(5)2 with 18.5, S3(3) with 14.4, SS3(4)1 with 12.7, and SS3(5)1 with 7.3. Application of the bacterial isolate improved the germination energy (SS3(2)2, 53.8%; SS3(6)1, 38.4%; SS3(8), 15.3%; S3(1), 23.1%; SS3(9), 30.9%; SS3(2)1, 23.1%; SS3(5)2, 15.3%), seedling vigor index 1 (SS3(2)2, 80.9%; SS3(6)1, 44.7%; SS3(8), 47.4%; S3(1), 23.6%; SS3(9), 27.7%; SS3(2)1, 13.2%; SS3(5)2, 21%; S3(3), 49%; SS3(4)1, 37.1%; and SS3(5)1, 10.7%), seedling vigor index 2 (SS3(2)2, 60.5%; SS3(6)1, 32.6%; SS3(8), 38.2%; S3(1), 19.2%; SS3(9), 15.3%; SS3(2)1, 11.5%; SS3(5)2, 2.2%; S3(3), 37.4%; SS3(4)1, 36.4%; and SS3(5)1, 5.8%), average number of roots (SS3(2)2, 12.8%; SS3(6)1, 27.7%; SS3(8), 21.3%; S3(1), 42.6%; SS3(9), 42.6%; SS3(2)1, 12.8%; SS3(5)2, 6.4%; S3(3), 34%; SS3(4)1, 34%; and SS3(5)1, 48.9%), emerging shoot height (SS3(2)2, 43.9%; SS3(6)1, 14.6%; SS3(8), 22%; S3(1), 12.2%; SS3(2)1, 24.4%; SS3(5)2, 24.4%; S3(3), 22%; and SS3(4)1, 9.8%), emerging root length (SS3(2)2, 571.4%; SS3(6)1, 342.9%; SS3(8), 171.4%; S3(1), 171.4%; SS3(9), 157.1%; SS3(2)1, 142.9%; SS3(5)2, 300%; S3(3), 228.6%; SS3(4)1, 342.9%; and SS3(5)1, 171.4%), emerging shoot mass (SS3(2)2, 40.6%; SS3(6)1, 21.9%; SS3(8), 28.1%; S3(1), 25%; SS3(9), 3.1%; SS3(2)1, 15.6%; SS3(5)2, 3.1%; S3(3), 25%; and SS3(4)1, 40.6%), emerging root mass (SS3(2)2, 83.9%; SS3(6)1, 45.2%; SS3(8), 48.4%; S3(1), 38.7%; SS3(9), 29%; SS3(2)1, 19.4%; SS3(5)2, 51.6%; S3(3), 51.6%; SS3(4)1, 45.2%; and SS3(5)1, 12.9%), and emerging root shoot ratio (SS3(2)2, 25%; SS3(6)1, 25%; SS3(8), 12.5%; S3(1), 12.5%; SS3(9), 37.5%; SS3(5)2, 12.5%; S3(3), 12.5%; SS3(4)1, 37.5%; and SS3(5)1, 37.5%) with respect to control.

Most of the selected isolates from *A. pseudoplatanus* showed overall positive effect ranging from low to moderate on the germination of rice (see **Figure 6**); however, few isolates—SS4(10), SS4(7), L4(1), and S4(2)—were found to influence some of the germination parameters negatively. Among the tested isolates, SS4(5) recorded with the highest score of 48.6 followed by SS4(8) with 43.3, SS4 (10) with 38, R4(1)2 with 35.6, S4(2) with 24.8, L4(1) with 11.6, and SS4(7) with 5.8 as mentioned in **Table 7**. Biopriming with bacterial isolates enhanced the seedling vigor index 1 (SS4(5), 133%; SS4(8), 75.6%; SS4(10), 129.8%; R4(1)2, 82.3%; S4(2), 8.7%; L4(1), 2.5%; and SS4(7), 9.6%), seedling vigor index 2 (SS4(5), 90%; SS4(8), 53.7%; SS4(10), 81.6%; R4(1)2, 51.1; S4(2), 22.1%; and SS4(7), 11.6%), average number of roots (SS4(5), 27.7%; SS4(8), 34%; SS4(10), 21.3%; R4(1)2, 12.8%; S4(2), 21.3%; L4(1), 27.7%; and SS4(7), 34%), emerging shoot height (SS4(5), 36.6%; SS4(8), 31.7%; SS4(10), 36.6%; R4(1)2, 39%; S4(2), 31.7%; L4(1), 19.5%; and SS4(7), 2.4%), emerging root length (SS4(5), 742.9%; SS4(8), 500%; SS4 (10), 914.3%; R4(1)2, 700%; S4(2), 300%; L4(1), 171.4%; and SS4(7), 142.9%), emerging shoot mass (SS4(5), 46.9%; SS4(8), 31.3%; SS4(10), 34.4%; R4(1)2, 25%; S4(2), 50%; and SS4(7), 12.5%), emerging root mass (SS4(5), 135.5%; SS4(8), 77.4%; SS4(10), 132.3%; R4(1)2, 93.5%; S4(2), 22.6%; L4(1), 12.9%; and SS4(7), 9.7%), and emerging root shoot ratio (SS4(5), 62.5%; SS4(8), 25%; SS4 (10), 62.5%; and R4(1)2, 25%) in comparison with control.

**Figure 7:**
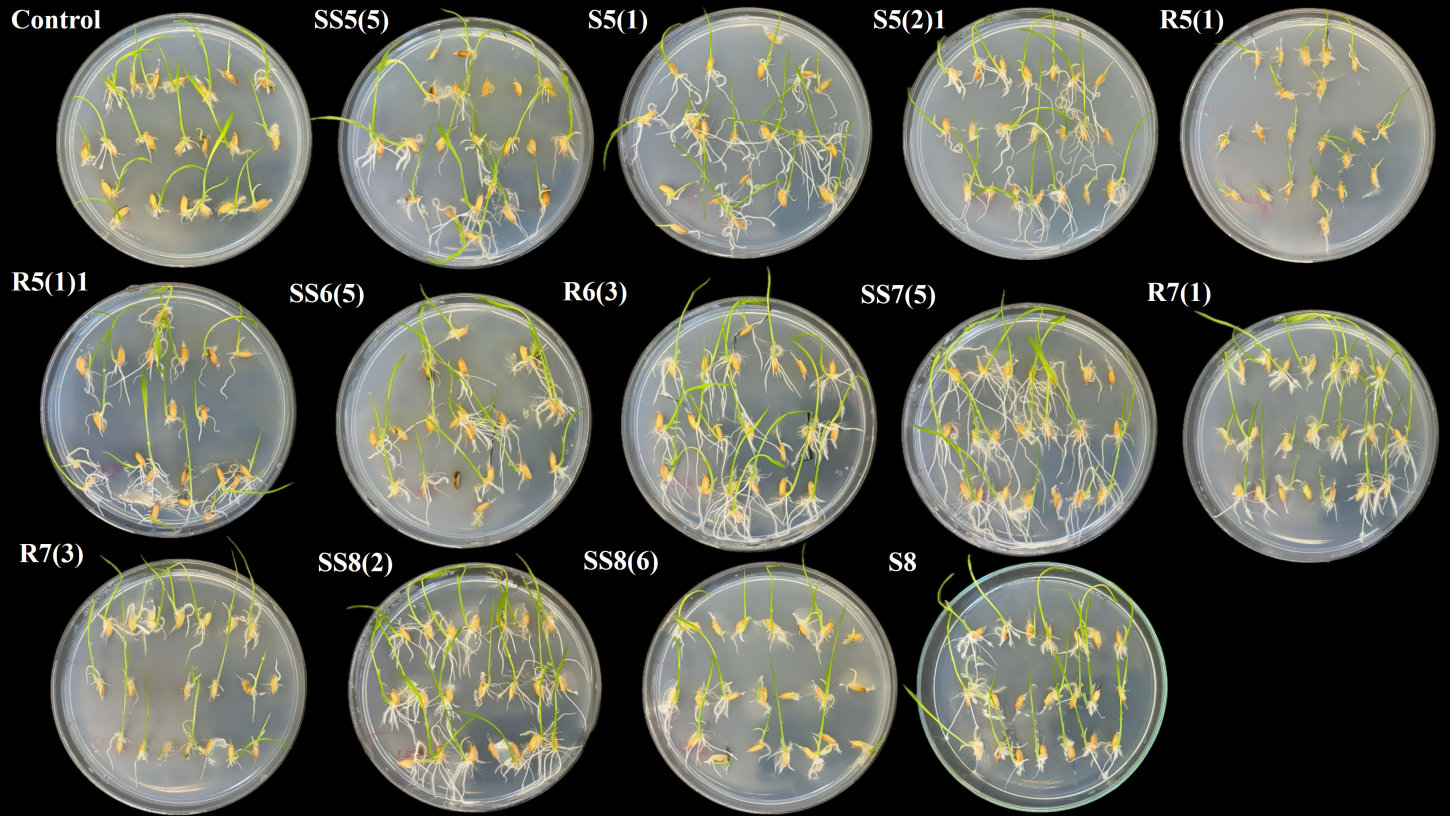
Impact of seed biopriming with selected plant growth-promoting bacteria isolated from rhizosphere soils of *Robinia pseudoacacia, Juglans regia, Malus domestica*, and *Ficus carica* on the early growth of emerging rice seedlings in comparison to control.

From the rhizosphere of *R. pseudoacacia*, isolate R5(1) (score −3.1) exhibited an overall negative impact on germination as shown in **Table 7** and **Figure 7**; opposite to this, isolate S5(2)1 showed the highest score of 50 followed by S5(1) with 16.4, R5(1)1 with 13.9, and SS5(5) with 11.1 as detailed in **Table 7**. Biopriming with bacterial isolates increased the germination energy (S5(2)1, 46.2%; and R5(1), 23.1%), seedling vigor index 1 (S5(2)1, 101.1%; S5(1), 66%; R5(1)1, 42.6%; and SS5(5), 35.4%), seedling vigor index 2 (S5(2)1, 56.3%; S5(1), 35.8%; R5(1)1, 30.5%; and SS5(5), 33.5%), average number of roots (S5(2)1, 34%; S5(1), 21.5%; R5(1)1, 21.3%; and SS5(5), 12.8%), emerging shoot height (S5(2)1, 7.3%; R5(1)1, 24.4%; and SS5(5), 17.1%), emerging root length (S5(2)1, 528.6%; S5(1), 414.3%; R5(1)1, 300%; and SS5(5), 285.7%), emerging shoot mass (S5(2)1, 12.5%; S5(1), 6.2%; R5(1)1, 18.8%; and SS5(5), 37.5%), emerging root mass (S5(2)1, 103.2%; S5(1), 67.7%; R5(1)1, 45.2%; and SS5(5), 45.2%), and emerging root shoot ratio (S5(2)1, 75%; S5(1), 87.5%; R5(1)1, 12.5%; and SS5(5), 12.5%) with respect to control.

Isolate R6(3) got the highest score of 56.2 followed by SS6(5) with 40.3 in the germination trial among the isolates obtained from the rhizosphere soil of *J. regia* as shown in **Table 7** and **Figure 7**, and their application improved the GE (R6(3), 30.9%; SS6(5), 23.1%), SVI1 (R6(3), 149.3%; SS6(5), 76.6%), SVI2 (R6(3), 79.9%; SS6(5), 66.3%), Avg. no. of roots ((R6(3), 34%; SS6(5), 34%), ESH ((R6(3), 39%; SS6(5), 26.8%), ERL (R6(3), 485.7%; SS6(5), 442.9%), ESM (R6(3), 56.3%; SS6(5), 18.8%), ERM (R6(3), 164.5%; SS6(5), 77.4%), and ERS ratio (R6(3), 75%; SS6(5), 37.5%) as compared to the control.

Isolate SS7(5) showed the best performance (score 59.7) followed by R7(1) with 31.4 and R7(3) with 21.2 among the selected isolates derived from the root zone of *M. domestica* as mentioned in **Table 7** and **Figure 7**. Treatment with bacterial isolates enhanced the GE (R7(1), 15%; R7(3), 38%), SVI1 (SS7(5), 171.6%; R7(1), 37.1%), SVI2 (SS7(5), 118.6%; R7(1), 37.1%; R7(3), 0.3%), Avg. no. of roots (SS7(5), 21.3%; R7(1), 42.6%), ESH (SS7(5), 29.3%; R7(1), 31.7%; R7(3), 22%), ERL (SS7(5), 814.3%; R7(1), 328.6%; R7(3), 200%), ESM (SS7(5), 75%; R7(1), 43.8%; R7(3), 18.8%), ERM (SS7(5), 187.1%; R7(1), 45.2%; R7(3), 6.5%), and ERS ratio (SS7(5), 112.5%) with respect to control.

Bacterial isolate SS8(2) showed the most positive outcome (score 54) followed by S8 with 25.5 and SS8(6) with 24.8, among the isolates derived from the rhizosphere soil of *F. carica* in the germination trial as shown in **Table 7** and **Figure 7**. Seed biopriming with these isolates enhanced the GE (SS8(2), 23.1%; S8, 23.1%; SS8(6), 15.3%), SVI1 (SS8(2), 137.6%; S8, 28.7%; SS8(6), 29.8.3%), SVI2 (SS8(2), 80.4%; S8, 16.2%; SS8(6), 33.2%), Avg. no. of roots (SS8(2), 27.7%; S8, 42.6%; SS8(6), 27.7%), ESH (SS8(2), 43.9%; S8, 24.4%; SS8(6), 26.8%), ERL (SS8(2), 428.6%; S8, 142.9%; SS8(6), 85.7%), ESM (SS8(2), 31.3%; S8, 9.4%; SS8(6), 37.5%), ERM (SS8(2), 151.6%; S8, 38.7%; SS8(6), 32.3%), and ERS ratio (SS8(2), 62.5%; S8, 12.5%) with respect to control.

**Table 7:** Effects of biopriming rice seeds with selected plant growth-promoting bacteria on germination parameters.

Rhizosphere soil	Isolate	Source of isolation	ERL (cm) 10^c^	ESH (cm) 10	ERM (mg) 10	ESM (mg) 10	Avg. no. of roots 10	GE 20	SVI1 10	SVI2 10	ERS ratio 10	Total score (100)
*Abies nordmanniana*	SS1(1)	Soil	5.2 ± 1.3*^a^ 5.3^b^	5.6 ± 0.4* 6.3	6.8 ± 0.3* 6.2	4.6 ± 0.5 5.8	6 ± 1 2.8	66.7 ± 16.5 2.9	680 ± 30* 6.1	1,140 ± 78.1* 6.7	1.2 ± 0 4.4	46.6
	SS1(4)		4.8 ± 0.5* 4.8	5.3 ± 0.4 5	6.3 ± 1.1* 5.3	4.2 ± 0.2 4.2	5.7 ± 0.6 2.2	81 ± 21.8 11.5	630 ± 110* 5.3	1,053.3 ± 95* 5.6	1.2 ± 0.3 4.4	48.3
	S1(B)	Rice stem	4.1 ± 1.7* 4	5 ± 0.6 3.8	5.2 ± 1.2* 3.5	4 ± 1.5 3.3	6 ± 0 2.8	81 ± 8.2* 11.5	515 ± 115 3.4	918.3 ± 262.8 3.8	1 ± 0.3 2.2	38.3
	S1(E)		9.2 ± 2.9* 10	5.9 ± 0.4* 7.5	9.1 ± 0.2* 10	4.5 ± 0.6 5.4	5.3 ± 0.6 1.3	95.2 ± 8.2 20	910 ± 20* 10	1,356.7 ± 73.7* 9.6	1.5 ± 0.1* 7.8	81.6
	S1(G)		3.2 ± 0.3* 2.9	5.6 ± 0.4* 6.3	5.1 ± 0.6* 3.3	4.3 ± 0.3 4.6	6 ± 0 2.8	81 ± 8.2 11.5	489 ± 97 2.9	900 ± 156.2 3.6	0.9 ± 0.2 1.1	39.0
	R1(B)	Rice roots	2.8 ± 0.8 2.5	4.5 ± 0.2 1.7	5 ± 2.1 3.2	3.2 ± 1 0	4.7 ± 0.6 0	81 ± 21.8* 11.5	466.7 ± 190.9 2.6	772.9 ± 282.7 1.9	1.1 ± 0.4 3.3	26.5
*Corylus avellana*	SS2(1)2	Soil	4 ± 0.5* 3.9	5.9 ± 1* 7.5	7.1 ± 1.2* 6.7	4.2 ± 0.3 4.2	6 ± 1 2.8	71.4 ± 14.3 5.7	676.2 ± 149* 6.1	1,074.8 ± 167.4* 5.9	1.2 ± 0.4* 4.4	47.2
	SS2(4)		2.6 ± 0.1 2.2	5 ± 0.4 3.8	5.1 ± 0.6* 3.3	3.7 ± 0.7 2.1	9.3 ± 1.2* 10	76.2 ± 8.2 8.6	485.7 ± 73.3 2.9	834.3 ± 62.7 2.7	1 ± 0.1 2.2	37.8
	SS2(4)2		3 ± 0.6 2.7	5.3 ± 0.5 5	4.8 ± 0.6 2.8	3.8 ± 0.3 2.5	6.7 ± 0.6* 4.3	71.4 ± 14.3 5.7	439 ± 122.8 2.1	783.3 ± 168 2	0.9 ± 0.1 1.1	28.3
	L2(6)2	Rice leaves	3.8 ± 0.3*3.6	5.3 ± 0.3 5	4.7 ± 0.3 2.7	4 ± 0.8 3.3	5 ± 1 0.7	85.7 ± 14.3* 14.3	466.7 ± 28.9 2.6	870 ± 75.5 3.2	0.9 ± 0.1 1.1	36.4
	R2(2)2	Rice roots	7 ± 1.1* 7.4	6.5 ± 0.3* 10	6.3 ± 0.5* 5.3	4.7 ± 0.3 6.3	5.7 ± 0.6 2.2	90.5 ± 8.2 17.2	630 ± 50* 5.3	1,103.3 ± 40.4* 6.3	1 ± 0.1 2.2	62.1
*Pyrus elaeagnifolia*	SS3(2)1	Soil	1.7 ± 1 1.2	5.1 ± 0.7 4.2	3.7 ± 1.3 1	3.7 ± 0.4 2.1	5.3 ± 1.2 1.3	76.2 ± 8.2 8.6	354.8 ± 143.1 0.7	706.2 ± 185.4 1	0.8 ± 0.4 0	20.0
	SS3(2)2		4.7 ± 0.2* 4.7	5.9 ± 0.4* 7.5	5.7 ± 0.7* 4.3	4.5 ± 0.8 5.4	5.3 ± 0.6 1.3	95.2 ± 8.2 20	566.7 ± 65.1* 4.2	1,016.7 ± 127* 5.1	1 ± 0.1 2.2	54.8
	SS3(4)1		3.1 ± 0.9* 2.8	4.5 ± 1.1 1.7	4.5 ± 0.8 2.3	4.5 ± 1.9 5.4	6.3 ± 0.6* 3.5	42.9 ± 14.3 −11.4	429.5 ± 79.6 1.9	863.8 ± 276.1 3.1	1.1 ± 0.4 3.3	12.7
	SS3(5)1		1.9 ± 0.6 1.4	3.1 ± 0.3 −4.2	3.5 ± 1 0.7	3.2 ± 0.8 0	7 ± 0* 5	61.9 ± 16.5 0	346.7 ± 98.7 0.6	670 ± 81.9 0.5	1.1 ± 0.4 3.3	7.3
	SS3(5)2		2.8 ± 1.4 2.5	5.1 ± 0.1 4.2	4.7 ± 0.3 2.7	3.3 ± 0.3 0.4	5 ± 1 0.7	71.4 ± 14.3 5.7	379 ± 46.7 1.1	647.1 ± 78.7 0.2	0.9 ± 0 1.1	18.5
	SS3(6)1		3.1 ± 0.9* 2.8	4.7 ± 0.8 2.5	4.5 ± 0.8 2.3	3.9 ± 0.4 2.9	6 ± 1 2.8	85.7 ± 14.3 14.3	453.3 ± 83.9 2.3	840 ± 115.3 2.8	1 ± 0 2.2	35.0
	SS3(8)		1.9 ± 0.9 1.4	5 ± 0.2 3.8	4.6 ± 0.5 2.5	4.1 ± 1.2 3.8	5.7 ± 0.6 2.2	71.4 ± 24.7 5.7	461.7 ± 53.5 2.5	875 ± 163.9 3.2	0.9 ± 0.1 1.1	26.1
	SS3(9)		1.8 ± 0.4 1.3	3.8 ± 0.8 −1.3	4 ± 0.4 1.5	3.3 ± 1.1 0.4	6.7 ± 1.2* 4.3	81 ± 21.8 11.5	400 ± 43.6 1.5	730 ± 122.9 1.3	1.1 ± 0.2 3.3	23.9
	S3(1)	Rice stem	1.9 ± 0.2 1.4	4.6 ± 0.4 2.1	4.3 ± 0.8 2	4 ± 0.6 3.3	6.7 ± 0.6* 4.3	76.2 ± 16.5 8.6	387.1 ± 84.2 1.2	755.2 ± 156.5 1.6	0.9 ± 0.2 1.1	25.7
	S3(3)		2.3 ± 0.4 1.9	5 ± 0.7 3.8	4.7 ± 0.2 2.7	4 ± 0.5 3.3	6.3 ± 0.6* 3.5	47.6 ± 8.2 −8.6	466.7 ± 15.3 2.6	870 ± 60 3.2	0.9 ± 0.1 1.1	13.4
*Acer pseudoplatanus*	SS4(5)	Soil	5.9 ± 0.3* 6.1	5.6 ± 0.5* 6.3	7.3 ± 1.1* 7	4.7 ± 0.4 6.3	6 ± 0 2.8	61.9 ± 8.2 0	730 ± 110* 7	1,203.3 ± 94.5* 7.6	1.3 ± 0.3* 5.6	48.6
	SS4(7)		1.7 ± 0.6 1.2	4.2 ± 0.2 0.4	3.4 ± 1.3 0.5	3.6 ± 0.9 1.7	6.3 ± 0.6* 3.5	57.1 ± 37.8 −2.9	343.3 ± 125 0.5	706.7 ± 170.4 1	0.8 ± 0.3 0	5.8
	SS4(8)		4.2 ± 0.6* 4.1	5.4 ± 0.2* 5.4	5.5 ± 0.3* 4	4.2 ± 1 4.2	6.3 ± 0.6* 3.5	81 ± 21.8 11.5	550 ± 26.5* 4	973.3 ± 126.6 4.5	1 ± 0.1 2.2	43.4
	SS4(10)		7.1 ± 0.7* 7.5	5.6 ± 0.5* 6.3	7.2 ± 0.4* 6.8	4.3 ± 0.2 4.6	5.7 ± 0.6 2.2	47.6 ± 8.2 −8.6	720 ± 40* 6.8	1,150 ± 34.6* 6.9	1.3 ± 0.1* 5.6	38.0
	L4(1)	Rice leaves	1.9 ± 0.4 1.4	4.9 ± 0.9 3.3	3.5 ± 0.2 0.7	3 ± 0.2 −0.8	6 ± 0 2.8	71.4 ± 14.3 5.7	321 ± 70.5 0.1	593.8 ± 111.6 −0.5	0.7 ± 0.1 −1.1	11.6
	S4(2)	Rice stem	2.8 ± 0.7 2.5	5.4 ± 0.2* 5.4	3.8 ± 0.3 1.2	4.8 ± 1 6.7	5.7 ± 0.6 2.2	71.4 ± 0 5.7	340.5 ± 36.3 0.5	773.3 ± 144.7 1.9	0.7 ± 0 −1.1	24.8
	R4(1)2	Rice roots	5.6 ± 1* 5.8	5.7 ± 0.3* 6.7	6 ± 0.6* 4.8	4 ± 0.5 3.3	5.3 ± 0.6 1.3	66.7 ± 21.8 2.9	571 ± 69* 4.3	957.1 ± 112.9 4.3	1 ± 0.1 2.2	35.6
*Robinia pseudoacacia*	SS5(5)	Soil	2.7 ± 0.5 2.4	4.8 ± 0.5 2.9	4.5 ± 0.2 2.3	4.4 ± 0.3 5	5.3 ± 0.6 1.3	47.6 ± 16.5 −8.6	424.3 ± 19.2 1.9	845.2 ± 86.3 2.8	0.9 ± 0.1 1.1	11.1
	S5(1)	Rice stem	3.6 ± 0.8* 3.4	3.6 ± 0.5 −2.1	5.2 ± 0.5* 3.5	3.4 ± 0.3 0.8	5.7 ± 0.6 2.2	52.4 ± 8.2 −5.7	520 ± 45.8 3.5	860 ± 70 3	1.5 ± 0.3* 7.8	16.4
	S5(2)1		4.4 ± 1.5* 4.4	4.4 ± 0.2 1.3	6.3 ± 0.2* 5.3	3.6 ± 0.9 1.7	6.3 ± 0.6* 3.5	90.5 ± 8.2 17.2	630 ± 20* 5.3	990 ± 87.2 4.7	1.4 ± 0* 6.7	50.0
	R5(1)	Rice roots	0.4 ± 0.1 −0.4	3.1 ± 0.4 −4.2	2.5 ± 0.2 −1	2 ± 0.2 −5	5.7 ± 0.6 2.2	76.2 ± 8.2 8.6	253.3 ± 23.1 −1	456.7 ± 37.9 −2.4	0.8 ± 0.1 0	−3.1
	R5(1)1		2.8 ± 0.3 2.5	5.1 ± 0.4 4.2	4.5 ± 0.4 2.3	3.8 ± 0.7 2.5	5.7 ± 0.6 2.2	52.4 ± 21.8 −5.7	446.7 ± 40.4 2.2	826.7 ± 87.4 2.6	0.9 ± 0.1 1.1	13.9
*Juglans regia*	SS6(5)	Soil	3.8 ± 1* 3.6	5.2 ± 0.3 4.6	5.5 ± 0.6* 4	5 ± 0.5 7.5	6.3 ± 0.6* 3.5	76.2 ± 8.2 8.6	553.3 ± 55.1* 4	1,053.3 ± 105* 5.6	1.1 ± 0.1 3.3	44.7
	R6(3)	Rice roots	4.1 ± 0.5* 4	5.7 ± 0.3* 6.7	8.2 ± 0.6* 8.5	3.8 ± 0.3* 2.5	6.3 ± 0.6* 3.5	81 ± 8.2 11.5	781 ± 120* 7.8	1,139.5 ± 148.8* 6.7	1.4 ± 0.1* 6.7	57.9
*Malus domestica*	SS7(5)	Soil	6.4 ± 1.9* 6.7	5.3 ± 0.3 5	8.9 ± 0.6* 9.7	5.6 ± 0.3* 10	5.7 ± 0.6 2.2	57.1 ± 14.3 −2.9	851 ± 94.1* 9	1,384.3 ± 125* 10	1.7 ± 0* 10	59.7
	R7(1)	Rice roots	3 ± 0.2 2.7	5.4 ± 0.3* 5.4	4.5 ± 0.2 2.3	4.6 ± 0.6 5.8	6.7 ± 0.6* 4.3	71.4 ± 14.3 5.7	429.5 ± 53.7 1.9	868.1 ± 118.1 3.1	0.8 ± 0.1 0	31.4
	R7(3)		2.1 ± 0.1 1.6	5 ± 0.5 3.8	3.3 ± 0.2 0.3	3.8 ± 0.4 2.5	4.7 ± 0.6 0	85.7 ± 0 14.3	296.7 ± 46.4 −0.3	635.2 ± 40.8 0	0.7 ± 0.1 −1.1	21.2
*Ficus carica*	SS8(2)	Soil	3.7 ± 0.8* 3.5	5.9 ± 0.4* 7.5	7.8 ± 0.3* 7.8	4.2 ± 0.7 4.2	6 ± 0 2.8	76.2 ± 16.5* 8.6	744.3 ± 89.1* 7.2	1,142.4 ± 166.4* 6.8	1.3 ± 0.1* 5.6	54.0
	SS8(6)		1.3 ± 0.3 0.7	5.2 ± 0.6 4.6	4.1 ± 0.4 1.7	4.4 ± 1.2 5	6 ± 0 2.8	71.4 ± 14.3 5.7	406.7 ± 41.6 1.6	843.3 ± 75.7 2.8	0.8 ± 0.1 0	24.8
	S8	Rice stem	1.7 ± 0.3 1.2	5.1 ± 0.7 4.2	4.3 ± 1.7 2	3.5 ± 1.3 1.3	6.7 ± 0.6* 4.3	76.2 ± 21.8 8.6	403.3 ± 165.6 1.5	735.7 ± 295 1.4	0.9 ± 0.4 1.1	25.5
	Control		0.7 ± 0.1 0	4.1 ± 0 z.5 0	3.1 ± 0.2 0	3.2 ± 0.3 0	4.7 ± 0.6 0	61.9 ± 8.20	313.3 ± 15.3 0	633.3 ± 47.3 0	0.8 ± 0.1 0	0

Avg. no. of roots, average number of roots; ESH, emerging shoot height in centimeters; ERL, emerging root length in centimeters; ESM, emerging shoot mass in milligrams; ERM, emerging root mass in milligrams; SVI1, seedling vigor index 1; SVI2, seedling vigor index 2; GE, germination energy on the second day; ERS, emerging root shoot ratio.

a Mean value and standard deviation (*n* = 3 replicates).

b Actual obtained scores.

c Maximum scores allocated.

*Values are significantly different from the control within each column (Dunnett’s test, *P*< 0.05).

The application of selective bacterial isolates derived from different tree rhizospheres significantly improved the germination traits. These results demonstrated the potential of bacterial biopriming as an effective strategy for enhancing crop emergence and early growth.

### SEM observations of rice roots after bacterial inoculation

**Figure 8:**
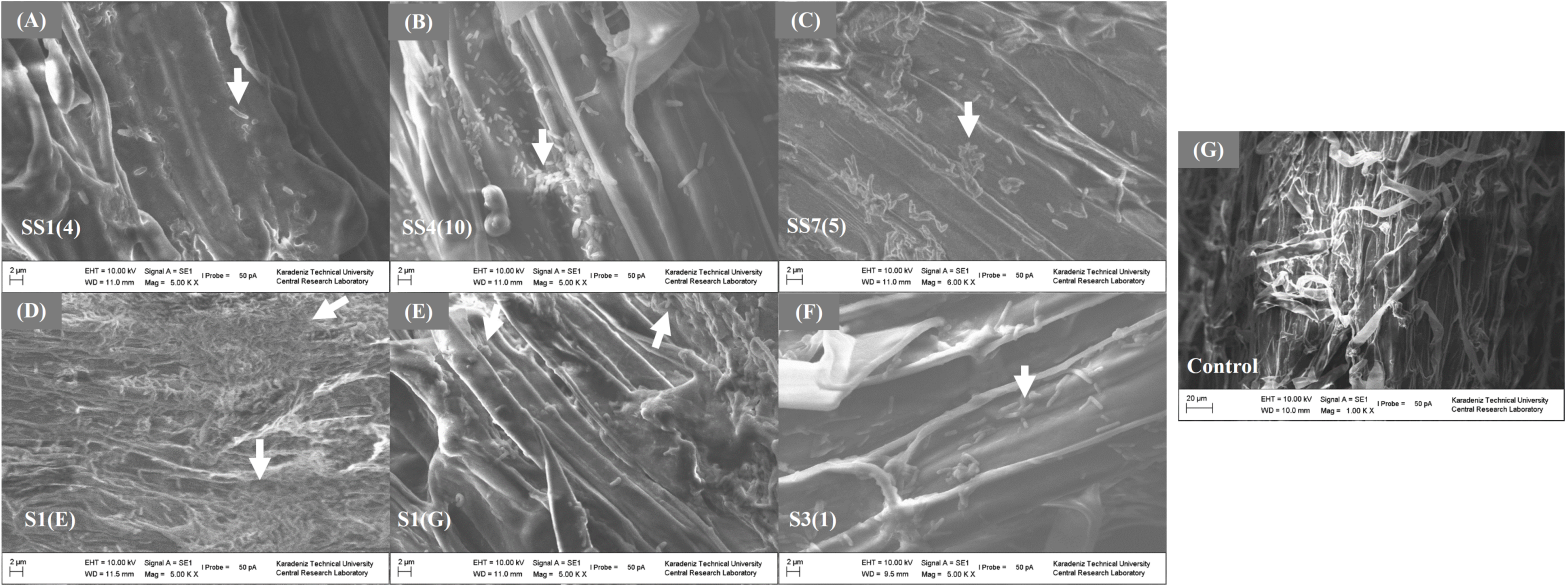
Scanning electron micrographs showing bacterial adherence on rice root surface following inoculation: **(A)** rice root inoculated with bacteria SS1(4); **(B)** distribution of SS4(10) bacteria on the root surface; **(C)** clusters of SS7(5) bacteria on the root surface; **(D)** dense coverage of the root by SS1**(E)** bacteria; **(E)** root inoculated with S1**(G)** bacteria; **(F)** clusters of SS3(1) bacteria attached to the root; **(G)** control (uninoculated root).

By analyzing the images taken by an SEM, one can gain insights into the interactions between rice roots and plant growth-promoting bacteria. The study on the efficiency of bacterial colonization and potential effects on root morphology is essential for understanding the beneficial relationships in plant–microbe interactions. SEM examination of rice root sections indicated a uniform distribution of isolate S1(E) in a large quantity across the root surface as shown in **Figure 8D**. In contrast, isolates SS1(4), SS4(10), S1(G), SS7(5), and S3(1) were mainly found segregated or clustered in the surface furrows at the junctions of epidermal cells (**Figures 8A-C, E, F**). Rice seedlings that were not inoculated with bacteria displayed a smooth and intact epidermal surface (**Figure 8G**). Observations revealed that roots inoculated with isolates S1(E) and SS7(5) exhibited high bacterial population colonized to the roots as compared to those inoculated with isolates SS1(4), SS4(10), S1(G), and S3(1), as depicted in **Figure 8**.

### Effect of bacterial inoculation on seedling growth in a hydroponic culture

**Figure 9:**
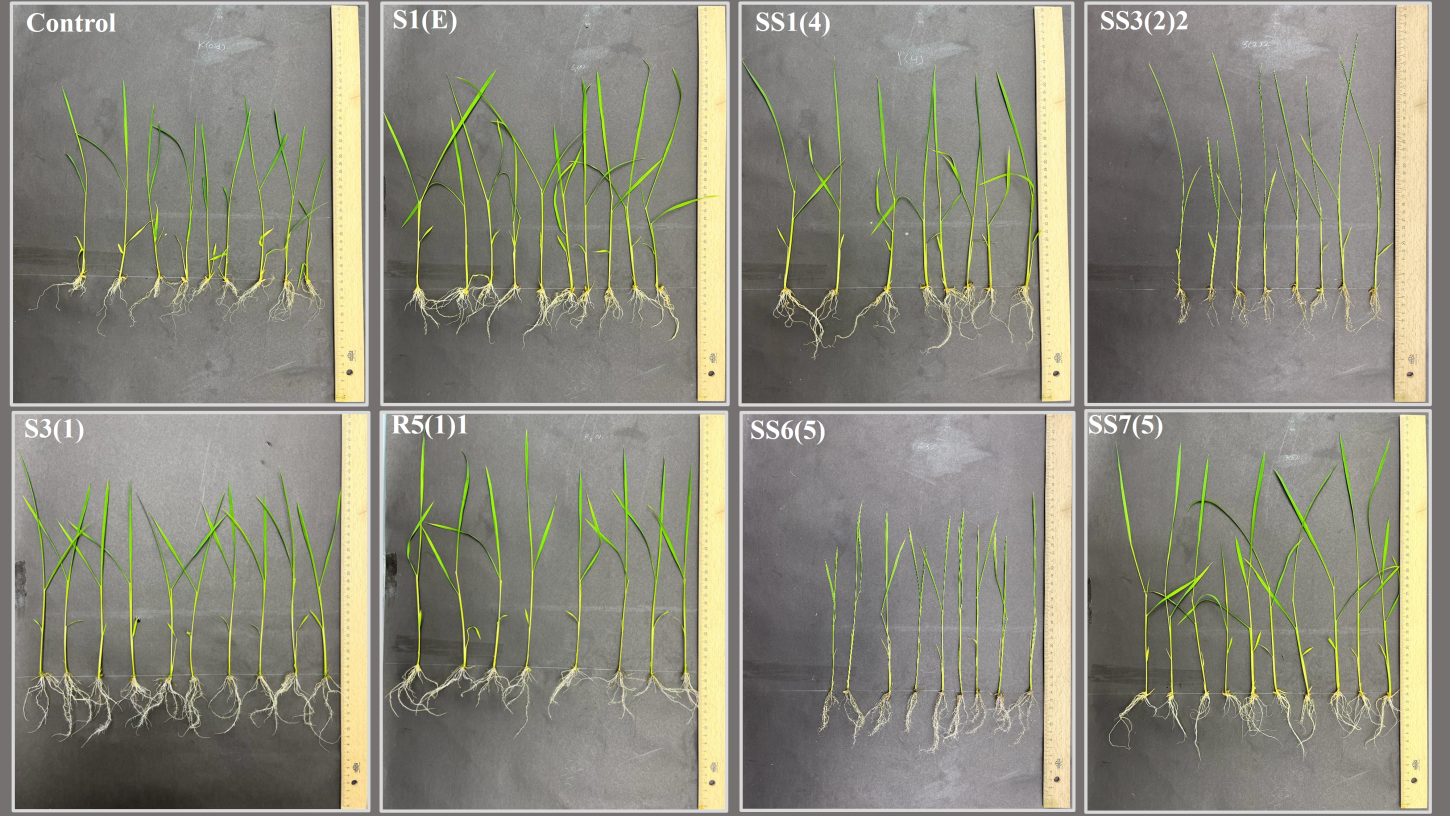
Effect of inoculating rice roots with different plant growth-promoting bacteria—S1(E), SS1(4), SS3(2)2, S3(1), R5(1)1, SS6(5), and SS4(5)—on the growth of 21-day-old seedlings in a hydroponic system.

The root inoculation with selected bacterial isolates exhibited remarkable influence on the growth of 21-day-old rice seedlings in a hydroponic culture, as detailed in **Table 8** and **Figure 9**.

Root inoculation with the selected bacteria isolated from the root zone of *A. nordmanniana* showed overall positive impact on rice growth; however, a few isolates like SS1(1) and R1(B) were found to influence some of the growth parameters negatively. Isolate S1(E) proved to be the most effective (see **Figure 9**) with the highest score of 56.2 followed by S1(B) with 54.7, S1(G) with 52.7, SS1(4) with 41.0, R1(B) with 35.7, and SS1(1) with 29.7 as detailed in **Table 8**. Inoculation of rice root with bacterial isolates boosted different growth parameters such as plant fresh weight (PFW) (S1(E), 147.6%; S1(B), 96.4%; S1(G), 77.1%; SS1(4), 54.5%; R1(B), 89.7%; and SS1(1), 47.2%), shoot height (SH) (S1(E), 25.3%; S1(B), 39.1%; S1(G), 40.4%; SS1(4), 14.7%; and R1(B), 32.9%), root length (RL) (S1(E), 5.1%; S1(B), 30.4%; S1(G), 2.5%; and SS1(4), 36.7%), dry shoot mass (DSM) (S1(E), 55.7%; S1(B), 73.8%; S1(G), 86.9%; SS1(4), 32.1%; R1(B), 57.4%; and SS1(1), 15.6%), dry root mass (DRM) (S1(E), 60%; S1(B), 48.6%; S1(G), 53.2%; SS1(4), 52.2%; R1(B), 25.4%; and SS1(1), 53.3%), and root shoot (RS) ratio (S1(E), 25%; SS1(4), 25%; and SS1(1), 50%) as compared to the control (see **Table 8**, **Figure 9**).

Generally, the inoculation with the selective isolates from *C. avellana* improved the overall growth scoring except isolate R2(2)2 (score −4.9). However, some of the growth parameters including root length were negatively influenced with the application. Isolate SS2(1)2 exhibited the highest score of 41.1 followed by SS2(4)2 with 30.5, SS2(4) with 26.1, and L2(6)2 with 22.8 as mentioned in **Table 8**. Treatment with bacterial isolates increased plant fresh weight (SS2(1)2, 99.7%; SS2(4)2, 47.6%; SS2(4), 49.6; L2(6)2, 15%; and R2(2)2, 5.3%), shoot height (SS2(1)2, 7.6%; SS2(4)2, 32.9%), dry shoot mass (SS2(1)2, 54.4%; SS2(4)2, 39.2%; and L2(6)2, 7.6%), dry root mass (SS2(1)2, 78.1; SS2(4)2, 31.4%; L2(6)2, 47.6%; and R2(2)2, 4.8%), and root shoot ratio (SS2(1)2, 25%; L2(6)2, 50%; and R2(2)2, 25%) as compared to the control.

Among the selected isolates from the rhizosphere of *P. elaeagnifolia*, the impact of inoculating rice roots was positive on overall growth scoring except the isolates SS3(2)1 (score −15.5) and S3(3) (score −11.1). Some bacterial applications negatively affected certain growth parameters. Isolate S3(1) exhibited the highest score of 66.9 (see **Table 8**, **Figure 9**) followed by SS3(5)1 with 60.3, SS3(4)1 with 39.6, SS3(2)2 with 35.5, SS3(9) with 39.1, SS3(6)1 with 22.2, SS3(5)2 with 19.8, and SS3(8) with 6. Treatment with bacterial isolates showed notable improvements in PFW (S3(1), 68.4%; SS3(5)1, 47.5%; SS3(4)1, 49.9%; SS3(2)2, 44.7%; SS3(9), 82.1%; SS3(6)1, 33.3%; SS3(5)2, 15.3%; S3(3), 28.3%; SS3(2)1, 9.3%; and SS3(8), 6.6%), shoot height (S3(1), 13.8%; SS3(4)1, 17.3%; SS3(2)2, 17.3%; SS3(9), 28.9%; SS3(6)1, 20.9%; and SS3(5)2, 1.8%), root length (S3(1), 40.5%; SS3(5)1, 94%; SS3(4)1, 19%), dry shoot mass (S3(1), 58.2%; SS3(5)1, 43.9%; SS3(4)1, 39.7%; SS3(2)2, 44.3%; SS3(9), 65.4%; SS3(6)1, 30.4%; SS3(5)2, 48.5%; and SS3(8), 5.9%), dry root mass (S3(1), 112.4%; SS3(5)1, 86.7%; SS3(4)1, 47.6%; SS3(2)2, 55.2%; SS3(9), 44.8%; SS3(6)1, 23.8%; SS3(5)2, 47.6%; and SS3(8), 21.9%), and root shoot ratio (S3(1), 50%; SS3(5)1, 50%; SS3(4)1, 25%; SS3(2)2, 25%; SS3(8), 25%; S3(3), 25%; and SS3(2)1, 25%) in comparison with the control as detailed in **Table 8**.

From the *A. pseudoplatanus* rhizosphere, isolate L4(1) was particularly effective with the highest score of 54 followed by SS4(8) with 45.3, SS4(5) with 32, SS4(7) with 22.4, R4(1)2 with 19.5, SS4(10) with 10.4, and S4(2) with 8.1 as mentioned in **Table 8**. Treatments with different bacterial inoculations resulted in overall better growth; however, few bacterial isolates showed negative impacts on some of the growth parameters. The application of bacterial isolates improved plant fresh weight (L4(1), 55.5%; SS4(8), 66.7%; SS4 (5), 25.4%; SS4(7), 62.9%; R4(1)2, 31.1%; SS4(10), 6.3%; and S4(2), 16.1%), shoot height (L4(1), 11.1%; SS4(8), 11.6%; SS4(5), 12.4%; R4(1)2, 10.2; and SS4(7), 12.4), root length (L4(1), 19%; SS4(5), 5.1%; and SS4(10), 6.3%), dry shoot mass (L4(1), 71.3%; SS4(8), 31.6; SS4(5), 35.4%; R4(1)2, 13.1%; and SS4(7), 40.9%), dry root mass (L4(1), 97.1%; SS4(8), 69.5%; SS4(5), 46.7%; R4(1)2, 22.9%; SS4(7), 37.1; S4(2), 18.1%; and SS4(10), 19%), and root shoot ratio (L4(1), 25%; SS4(8), 50%; SS4(5), 25%; R4(1)2, 25%; S4(2), 50%; and SS4(10), 50%) as compared to the control as detailed in **Table 8**.

Among the selected isolates from the rhizosphere of *R. pseudoacacia*, root inoculation with isolate R5(1)1 exhibited better growth (see **Figure 9**) with the highest score of 57.7 followed by S5(1) with 51.1, SS5(5) with 26.4, S5(2)1 with 4.2, and R5(1) with −1.5. Treatment with bacterial isolates improved the plant fresh weight (R5(1)1, 108.8%; S5(1), 82.8%; SS5(5), 60.3%; and R5(1), 24.6%), shoot height (R5(1)1, 22.2%; S5(1), 25.8%; SS5(5), 29.8%; and R5(1), 8.9%), root length (R5(1)1, 17.7%; S5(1), 10.1%), dry shoot mass (R5(1)1, 58.6%; S5(1), 48.5%; SS5(5), 43.5%; S5(2)1, 0.4%; and R5(1), 10.1%), dry root mass (R5(1)1, 81.9%; S5(1), 72.4%; SS5(5), 24.8%; and S5(2)1, 12.4%), and root shoot ratio (25% increase for R5(1)1, S5(1), and S5(2)1) in comparison with the control. Isolates like S5(2)1 and R5(1) exhibited negative impacts on some of the growth parameters as mentioned in **Table 8**.

Isolate SS6(5) from the root zone of *J. regia* scored 44.6 (see **Table 8**, **Figure 9**). Its application enhanced PFW (44.6%), RL (6.3%), DSM (44.7%), DRM (88.6%), and RS ratio (50%) as compared to the control. In contrast, root inoculation with isolate R6(3) resulted in growth reduction (score −6.9).

Isolate SS7(5) from the rhizosphere of *M. domestica* exhibited the highest score of 49.4 (see **Table 8**, **Figure 9**) followed by R7(1) with 44.4, while isolate R7(3) exhibited negative effects (score −11.6) on rice growth. Treatment with isolates SS7(5) and R7(1) enhanced the plant fresh weight (118.2%, 80.4%), shoot height (37.8%, 28.9%), dry shoot mass (52.7%, 71.7%), and dry root mass (41.9%, 69.5%), respectively, as compared to the control. Isolate SS7(5) also exhibited an increase of 24.1% in root length than control.

Among the selected isolate from the root zone of *F. carica*, root inoculation with isolate SS8(2) showed the highest performance as a growth-improving agent with a score of 27.7 followed by SS8(6) with 11.6 and S8 with 3.9 as mentioned in **Table 8**. Treatment with SS8(2) increased PFW (41.8%), DSM (14.3%), DRM (44.8%), and RS ratio (50%).

**Table 8:** Impact of root inoculation with plant growth-promoting bacteria on the growth metrics of 3-week-old rice seedlings in a hydroponic culture.

Rhizosphere soil	Isolate	Source of isolation	PFW (mg) 15^c^	SH (cm) 15	RL (cm) 15	DSM (mg) 20	DRM (mg) 20	RS ratio 15	Total score (100)
*Abies nordmanniana*	SS1(1)	Soil	111.9 ± 4.8*^a^ 4.8^b^	21.8 ± 1.3 −1.2	6.9 ± 0.2 −2	27.4 ± 4.1 3.6	16.1 ± 2.4* 9.5	0.6 ± 0* 15	29.7
	SS1(4)		117.4 ± 23.8* 5.5	25.8 ± 2.6 5.4	10.8 ± 0.3 5.8	31.3 ± 6.2 7.4	16 ± 3.1* 9.3	0.5 ± 0 7.5	41
	S1(B)	Rice stem	149.3 ± 25* 9.8	31.3 ± 2.6* 14.5	10.3 ± 0.3 4.8	41.2 ± 6.6* 17	15.6 ± 3.2 8.6	0.4 ± 0 0	54.7
	S1(E)		188.2 ± 15.1* 15	28.2 ± 0.7 9.4	8.3 ± 0.3 0.8	36.9 ± 0.9* 12.8	16.8 ± 0.8* 10.7	0.5 ± 0 7.5	56.2
	S1(G)		134.6 ± 24.6* 7.8	31.6 ± 2.9* 15	8.1 ± 0.2 0.4	44.3 ± 3.8* 20	16.1 ± 1* 9.5	0.4 ± 0 0	52.7
	R1(B)	Rice root	144.2 ± 10* 9.1	29.9 ± 1.8* 12.2	6.2 ± 0.6 −3.4	37.3 ± 3.6* 13.2	13.2 ± 1.1 4.6	0.4 ± 0 0	35.7
*Corylus avellana*	SS2(1)2	Soil	151.8 ± 2* 10.1	24.2 ± 1.4 2.8	5 ± 0.5 −5.8	36.6 ± 4* 12.5	18.7 ± 2.6* 13.9	0.5 ± 0 7.5	41.1
	SS2(4)		113.7 ± 14.5* 5	27.3 ± 0.5 7.9	7.4 ± 0.5 −1	32.7 ± 2.8 8.7	13.7 ± 0.5 5.4	0.4 ± 0 0	26.1
	SS2(4)2		112.2 ± 11.9* 4.8	29.9 ± 1* 12.2	7.3 ± 0.2 −1.2	33 ± 1.7 9	13.8 ± 0.9 5.6	0.4 ± 0 0	30.5
	L2(6)2	Rice leaf	87.4 ± 7.4 1.5	20.1 ± 1.3 −4	7.9 ± 0.8 0	25.5 ± 3.1 1.7	15.5 ± 1.5 8.5	0.6 ± 0* 15	22.8
	R2(2)2	Rice root	80 ± 17.7 0.5	18.7 ± 1.9 −6.3	5.2 ± 0.6* −5.4	21.5 ± 3.5 −2.1	11 ± 1.7 0.8	0.5 ± 0 7.5	−4.9
*Pyrus elaeagnifolia*	SS3(2)1	Soil	83.1 ± 8.2 0.9	18.1 ± 1.7 −7.3	3.3 ± 0.2 −9.2	18.1 ± 3.6 −5.4	9.3 ± 1.6 −2	0.5 ± 0 7.5	−15.5
	SS3(2)2		110 ± 1.5* 4.5	26.4 ± 3.3 6.4	6.4 ± 0.7 −3	34.2 ± 4.1* 10.2	16.3 ± 1.9* 9.8	0.5 ± 0 7.5	35.5
	SS3(4)1		113.9 ± 11.8* 5.1	26.4 ± 2.6 6.4	9.4 ± 1 3	33.1 ± 3.9 9.1	15.5 ± 1.7 8.5	0.5 ± 0 7.5	39.6
	SS3(5)1		112.1 ± 13.3* 4.8	22.5 ± 0.9 0	15.4 ± 1.3* 15	34.1 ± 3.7* 10.1	19.6 ± 4.8* 15.4	0.6 ± 0.1* 15	60.3
	SS3(5)2		87.6 ± 9.8 1.6	22.9 ± 1.7 0.7	6.9 ± 0.4 −2	35.2 ± 4.1* 11.2	15.5 ± 1.4 8.5	0.4 ± 0 0	19.8
	SS3(6)1		101.3 ± 4.4 3.4	27.2 ± 1.4 7.7	7.8 ± 0.8 −0.2	30.9 ± 3.1 7	13 ± 1.4 4.2	0.4 ± 0.1 0	22.2
	SS3(8)		81 ± 5.1 0.7	20.2 ± 0.5 −3.8	6.1 ± 0.7 −3.6	25.1 ± 0.5 1.4	12.8 ± 0.4 3.9	0.5 ± 0 7.5	6
	SS3(9)		138.4 ± 2.7* 8.3	29 ± 0.9 10.7	6.4 ± 0.4 −3	39.2 ± 5* 15	15.2 ± 1.7 8	0.4 ± 0 0	39.1
	S3(1)	Rice stem	128 ± 5.7* 7	25.6 ± 0.5 5.1	11.1 ± 0.4* 6.4	37.5 ± 21* 13.4	22.3 ± 0.6* 20	0.6 ± 0* 15	66.9
	S3(3)		97.5 ± 32.1 2.9	18.5 ± 1.4 −6.6	3.5 ± 0.4* −8.8	19.7 ± 4.4 −3.9	9.2 ± 1.1 −2.2	0.5 ± 0.1 7.5	−11.1
*Acer pseudoplatanus*	SS4(5)	Soil	95.3 ± 5.7 2.6	25.3 ± 4.8 4.6	8.3 ± 0.1 0.8	32.1 ± 2 8.2	15.4 ± 1.3 8.3	0.5 ± 0.1 7.5	32
	SS4(7)		123.8 ± 7.8* 6.4	25.3 ± 1.5 4.6	5.6 ± 0.4 −4.6	33.4 ± 3.2 9.4	14.4 ± 1.4 6.6	0.4 ± 0 0	22.4
	SS4(8)		126.7 ± 4.2* 6.8	25.1 ± 2.4 4.3	7.7 ± 0.6 −0.4	31.2 ± 1.8 7.3	17.8 ± 0.5* 12.4	0.6 ± 0* 15	45.3
	SS4(10)		80.8 ± 5.5 0.6	19 ± 2.9 −5.8	8.4 ± 1.6 1	19.7 ± 3.6 −3.9	12.5 ± 3.9 3.4	0.6 ± 0.1* 15	10.4
	L4(1)	Rice leaf	118.2 ± 12.2* 5.6	25 ± 0.7 4.1	9.4 ± 0.8 3	40.6 ± 4.9* 16.4	20.7 ± 1.1* 17.3	0.5 ± 0 7.5	54
	S4(2)	Rice stem	88.2 ± 13.7 1.6	20 ± 3 −4.1	6.1 ± 2.1 −3.6	19.5 ± 3.6 −4.1	12.4 ± 1.4 3.2	0.6 ± 0.1* 15	8.1
	R4(1)2	Rice root	99.6 ± 1.1 3.2	24.8 ± 4.3 3.8	6.9 ± 0.8 −2	26.8 ± 5 3	12.9 ± 2 4.1	0.5 ± 0 7.5	19.5
*Robinia pseudoacacia*	SS5(5)	Soil	121.8 ± 10.9* 6.1	29.2 ± 1.9* 11	5.3 ± 0.3 −5.2	34 ± 4.7 10	13.1 ± 1.6 4.4	0.4 ± 0 0	26.4
	S5(1)	Rice stem	138.9 ± 6.9* 8.4	28.3 ± 0.8 9.6	8.7 ± 0.4 1.6	35.2 ± 0.3* 11.2	18.1 ± 0.3* 12.9	0.5 ± 0 7.5	51.1
	S5(2)1		73.1 ± 7.7 −0.4	20.8 ± 2.3 −2.8	6.7 ± 0.6 −2.4	23.8 ± 3.8 0.1	11.8 ± 2 2.2	0.5 ± 0 7.5	4.2
	R5(1)	Rice root	94.7 ± 8.2 2.5	24.5 ± 1.1 3.3	3.7 ± 0.2* −8.4	26.1 ± 1.4 2.3	9.8 ± 0.7 −1.2	0.4 ± 0 0	−1.5
	R5(1)1		158.7 ± 14.9* 11.1	27.5 ± 2.4 8.2	9.3 ± 0.6 2.8	37.6 ± 4.4* 13.5	19.1 ± 1.8* 14.6	0.5 ± 0 7.5	57.7
*Juglans regia*	SS6(5)	Soil	98.8 ± 14.5 3	22.2 ± 1.9 −0.5	8.4 ± 0.6 1	34.3 ± 3.4* 10.3	19.8 ± 0.1* 15.8	0.6 ± 0.1* 15	44.6
	R6(3)	Rice root	51 ± 5.8 −3.3	19 ± 0.6 −5.8	6.2 ± 0.3 −3.4	21.9 ± 2.3 −1.7	10.4 ± 0.8 −0.2	0.5 ± 0 7.5	−6.9
*Malus domestica*	SS7(5)	Soil	165.8 ± 17.2* 12	31 ± 2.3* 14	9.8 ± 0.1 3.8	36.2 ± 2.8* 12.1	14.9 ± 0.4 7.5	0.4 ± 0 0	49.4
	R7(1)	Rice root	137.1 ± 12.7* 8.2	29 ± 2.8 10.7	6.2 ± 0 −3.4	40.7 ± 5.4* 16.5	17.8 ± 2.9* 12.4	0.4 ± 0 0	44.4
	R7(3)		91.7 ± 17.2 2.1	16.7 ± 4.5 −9.6	4.5 ± 0.6* −6.8	19.4 ± 3.4 −4.2	10.1 ± 1.3 −0.7	0.5 ± 0.1 7.5	−11.6
*Ficus carica*	SS8(2)	Soil	107.8 ± 13.2* 4.3	23.4 ± 3.2 1.5	5.6 ± 0.8 −4.6	27.1 ± 2.2 3.3	15.2 ± 1.4 8	0.6 ± 0.1* 15	27.4
	SS8(6)		109.3 ± 15.2* 4.5	21.3 ± 3.8 −2	7 ± 0.4 −1.8	24.4 ± 3 0.7	12.1 ± 1.8 2.7	0.5 ± 0.1 7.5	11.6
	S8	Stem	65.9 ± 6.6 −1.4	18.6 ± 2.4 −6.4	5.4 ± 0.3 −5	20.7 ± 0.3 −2.9	13.2 ± 0.7 4.6	0.6 ± 0* 15	3.9
	Control		76 ± 11.8 0	22.5 ± 1.5 0	7.9 ± 0.6 0	23.7 ± 0 0	10.5 ± 1.2 0	0.4 ± 0.1 0	0.0

PFW, plant fresh weight in milligrams; SH, shoot height in centimeters; RL, root length in centimeters; DSM, dry shoot mass in milligrams; DRM, dry root mass in milligrams; RS, root shoot ratio.

a Maximum scores allocated.

b Mean value ± standard deviation (*n* = 3 replicates).

c Actual obtained scores.

*Values are significantly different from the control within each column (Dunnett’s test, *P*< 0.05).

These results underscore the potential effects of these bacterial isolates on enhancing various growth parameters of rice seedlings in hydroponic culture, demonstrating their effectiveness as growth-stimulating agents.

### Molecular identification of bacterial isolates

Of the 41 selected isolates, 14 bacterial isolates of different rhizospheric origins exhibited a significant impact on germination and growth traits of rice along with their diverse PGP traits. These isolates were selected for their molecular identification through 16S rRNA gene sequencing. The 16S rDNA sequences of the respective isolates were compared in the EzBioCloud database to find out similar sequences (**Table 9**). The homologous sequences were aligned to construct their phylogenetic trees for the examination of their relatedness with other close members (**Supplementary Figures 1S–4S**). The 16S rDNA gene sequences of the respective isolates were submitted to NCBI GenBank. The following accession numbers were obtained: PQ605795, S1(E); PQ605785, SS1(4); PQ605786, SS2(1)2; PQ605787, SS3(2)2; PQ605796, S3(1); PQ605788, SS4(5); PQ605789, SS4(8); PQ605794, R5(1)1; PQ605790, SS6(5); PQ605791, SS7(5); PQ605793, R(7)1; and PQ605792, SS8(2) (see **Table 9**). The sequence of the S1(E) isolate (source: *A. nordmanniana* rhizosphere) was found to be 99.85% similar to *Herbaspirillum huttiense* subsp. *huttiense*, isolate SS1(4) (source: *A. nordmanniana* rhizosphere) was 99.06% similar with *Viridibacillus arenosi*, SS2(1)2 isolate (source: *C. avellana* rhizosphere) showed 99.01% similarity with *Psychrobacillus faecigallinarum*, isolate SS3(2)2 (source: *P. elaeagnifolia* rhizosphere) exhibited 99.71% similarity with *Lysinibacillus fusiformis*, isolate S3(1) (source: *P. elaeagnifolia* rhizosphere) was 99.05% similar to *Lelliottia amnigena*, SS4(5) isolate (source: *A. pseudoplatanus* rhizosphere) showed 99.64% similarity with *Bacillus siamensis*, isolate SS4(8) (source: *A. pseudoplatanus* rhizosphere) was 99.93% similar with *Microbacterium phyllosphaerae*, isolate R4(1)2 (source: *A. pseudoplatanus* rhizosphere) was 100% similar with *Bacillus altitudinis*, isolates S5(2)1 and R5(1)1 (source: *R. pseudoacacia* rhizosphere) exhibited 100% and 99.85% similarity with *Acinetobacter calcoaceticus*, isolate SS6(5) (source: *J. regia* rhizosphere) displayed 99.42% similarity with *Micrococcus luteus*, SS7(5) and R7(1) isolates (source: *M. domestica* rhizosphere) were found to be 99.26% and 99.28% similar to *Pseudomonas mohnii* and *Lelliottia* sp. respectively, while isolate SS8(2) (source: *F. carica* rhizosphere) exhibited 99.43% similarity with *Staphylococcus succinus* (**Table 9**). It was found that selective isolates in the study belong to diverse genera, and the genus *Bacillus* was the single most dominant genus among the isolates (**Supplementary Figures 1S–4S**). Genetic diversity among isolates sourced from rhizosphere soils of different trees can be associated with their broad range in PGP traits and resulting effects on rice growth.

**Table 9:** Molecular identification of the most effective bacterial isolates based on their percent similarity in 16S rDNA sequence with the closest relatives in phylogeny.

Isolate label	Source of origin	GenBank accession no.	Homologous microorganism	% similarity
S1 (E)	*Abies nordmanniana* rhizosphere	PQ605795	*Herbaspirillum huttiense* subsp. *Huttiense*	99.85
SS1 (4)	*Abies nordmanniana* rhizosphere	PQ605785	*Viridibacillus arenosi*	99.06
SS2 (1)2	*Corylus avellana* rhizosphere	PQ605786	*Psychrobacillus faecigallinarum*	99.01
SS3 (2)2	*Pyrus elaeagnifolia* rhizosphere	PQ605787	*Lysinibacillus fusiformis*	99.71
S3 (1)	*Pyrus elaeagnifolia* rhizosphere	PQ605796	*Lelliottia amnigena*	99.05
SS4 (5)	*Acer pseudoplatanus* rhizosphere	PQ605788	*Bacillus siamensis*	99.64
SS4 (8)	*Acer pseudoplatanus* rhizosphere	PQ605789	*Microbacterium phyllosphaerae*	99.93
R4 (1)2	*Acer pseudoplatanus* rhizosphere	PV124212	*Bacillus altitudinis*	100
R5 (1)1	*Robinia pseudoacacia* rhizosphere	PQ605794	*Acinetobacter calcoaceticus*	99.85
S5 (2)1	*Robinia pseudoacacia* rhizosphere	PV439848	*Acinetobacter calcoaceticus*	100
SS6 (5)	*Juglans regia* rhizosphere	PQ605790	*Micrococcus luteus*	99.42
SS7 (5)	*Malus domestica* rhizosphere	PQ605791	*Pseudomonas mohnii*	99.26
R (7)1	*Malus domestica* rhizosphere	PQ605793	*Lelliottia* sp.	99.28
SS8 (2)	*Ficus carica* rhizosphere	PQ605792	*Staphylococcus succinus*	99.43

### Principal component analysis of PGP traits of isolates

**Figure 10:**
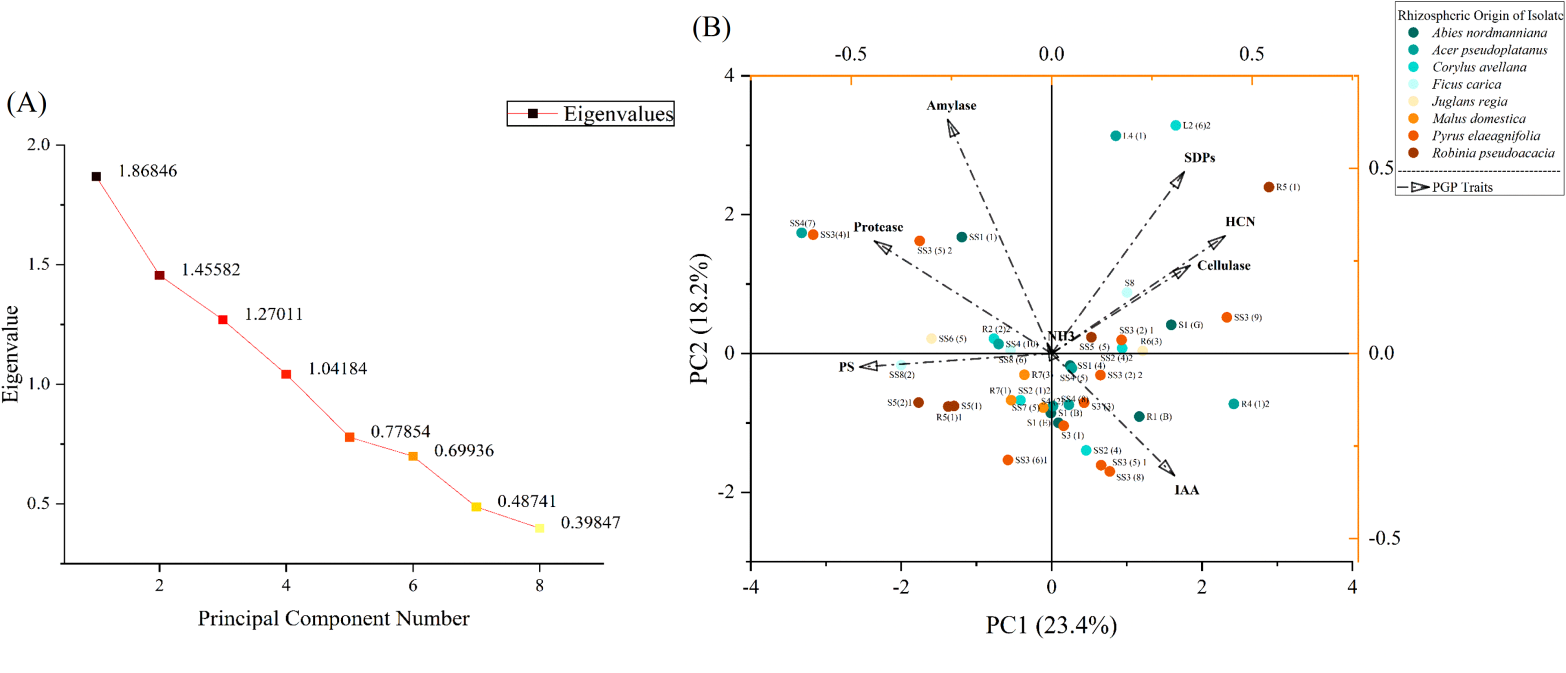
Principal component analysis (PCA) of PGP traits of bacterial isolates derived from different rhizospheres: **(A)** scree plot between eigenvalues and principal components (PCs); **(B)** biplot between PC1 and PC2.

Principal component (PC) 1 highly contributed (23.4%) to illustrate the variability in PGP traits of bacterial isolates followed by PC2 (18.2%), PC3 (15.9%), PC4 (13.1%), and PC5 (9.7%). The cumulative percentage of variance for the first five PCs was 80.2% along with the first four PCs exhibiting eigenvalues >1 (**Figure 10A**). PC1 was positively affected by SDP formation, HCN production, cellulase activity, and IAA, while it was negatively affected by amylase activity, protease activity, and PS. PC2 was positively influenced by almost all PGP traits except IAA and PS (**Figure 10B**).

Biplot represented each PGP trait with a unique loading vector starting from the origin and its relationship with different bacterial isolates derived from the rhizosphere of various trees. Furthermore, the biplot explained almost 41.6% of the variations in data based on PC1 and PC2 as shown in **Figure 10B**. All the vectors except NH_3_ dispersed away from the origin representing the high variability of PGP traits among bacterial isolates. Isolates L2(6)2, L4(1), and R5(1) showed high, S8 moderate, while SS5(5) and SS3(2)1 exhibited low association with SDPs. Isolate S3(9) demonstrated high, S1(G) moderate, and SS3(2)1, SS3(2)2, and SS1(4) displayed low association with HCN. S1(G) was highly linked, while R5(1) was moderately linked to cellulase activity. Also, three of the vectors (SDPS, HCN, cellulase) were highly interrelated; particularly, HCN and cellulase activity were more closely associated (**Figure 10B**). Majority of the isolates derived from the rhizosphere of eight different trees somehow exhibited high [i.e., SS3(8), SS3(5)1, SS2(4), and R1(B)], moderate, or low association with IAA. Production of IAA was slightly linked with cellulase activity and HCN production. Isolates S5(2)1, SS8(2), S5(1), and R5(1)1 displayed high and R7(1) presented moderate association with the PS vector. Protease activity was highly linked to SS3(5)2, SS3(4)1, SS4(7), SS6(5), and SS1(1); moderately linked to R2(2)2 and SS4(10); and poorly associated with SS8(6) and R7(3). Isolates SS1(1) and SS3(5)2 were closely associated with amylase activity. Three of the vectors (amylase, protease, PS) exhibited interrelationship with each other, where amylase activity was closely linked to protease activity which was closer to PS (**Figure 10B**).

Isolates derived from the rhizosphere of *R. pseudoacacia* were closely associated with PS activity. Most of the isolates derived from *A. nordmanniana* and *P. elaeagnifolia* were linked to IAA (**Figure 10B**). Similarly, isolates derived *A. nordmanniana*, *P. elaeagnifolia*, *R. pseudoacacia*, and *J. regia* were associated with HCN and cellulase activity. Meanwhile, some isolates from the root zone of *C. avellana*, *A. pseudoplatanus*, and *R. pseudoacacia* were linked to SDPs. Some isolates from these rhizospheres possessed the ability to localize within the root, stem, or leaf of rice plants. A few of the isolates derived from the root zone of *A. nordmanniana*, *P. elaeagnifolia*, and *A. pseudoplatanus* were also linked with protease and amylase activity (**Figure 10B**).

### Principal component analysis based on germination traits of rice upon biopriming with bacterial isolates

**Figure 11:**
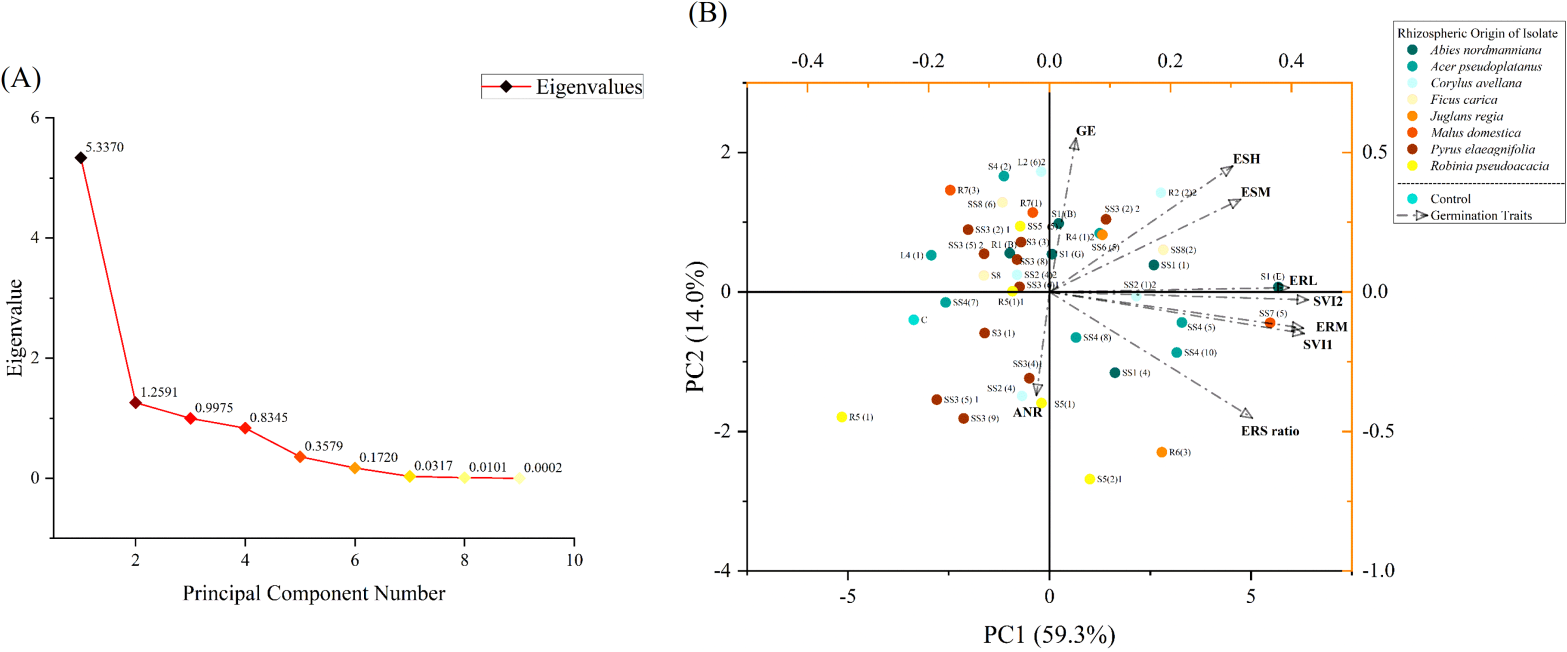
Principal component analysis (PCA) of bacterial isolates based on their impact on germination traits of rice: **(A)** scree plot between eigenvalues and principal components (PCs); **(B)** biplot between PC1 and PC2.

PCA revealed that PC1 highly contributed (59.3%) to explain the variability in germination traits of rice with the application of bacterial isolates followed by PC2 (14%), PC3 (11.1%), PC4 (9.3%), and PC5 (4%). Together, the first five principal components explained 97.6% of the cumulative variance, with the first two PCs having eigenvalues greater than 1 (**Figure 11A**). PC1 was positively influenced by all of the germination traits (ERS ratio, ERM, SVI2, ESM, ESH, SVI1, ERL, GE) except the average numbers of roots. PC2 was positively affected by GE, ESH, ESM, and ERL and negatively influenced by ANR, ERS ratio, ERM, SVI2, and SVI1 (**Figure 11B**).

The biplot displays each germination trait with specific loading vectors starting from the origin in correspondence with the influence of different bacterial isolates on it. Furthermore, the biplot elucidated almost 73.3% of the variability in data based on PC1 and PC2 as revealed in **Figure 11B**. All the vectors dispersed away from the origin with varying magnitudes displaying variability in germination traits upon biopriming the rice seeds with different bacterial isolates. Based on PC1, all the bacterial isolates except R5(1) showed better association with different germination traits as compared to the control (**Figure 11B**). The biplot illustrated that a large number of isolates were clustered around GE. Isolate R2(2)2 was highly associated, SS3(2)2, SS8(2), R4(1)2, SS6(5), and SS1(1) were moderately associated, and S1(G) and SS3(6)1 were slightly associated with ESH and ESM. Germination traits such as GE, ESH, ESM, and ERL exhibited interrelationship with each other. Isolates S1(E) and SS7(5) were highly associated, whereas SS4(5), SS2(1)2, SS4(10), SS1(4), SS4(8), and SS1(1) were moderately associated with ERL, SVI2, ERM, SVI1, and ERS ratio. Also, parameters such as ERL, SVI2, ERM, SVI1, and ERS ratio were interlinked with each other. Isolates S5(1), SS2(4), SS3(4), SS3(5)1, and SS3(9) were highly associated, S3(1) and SS4(8) were moderately associated, and R5(1)1 and SS3(6)1 were slightly associated with ANR. GE and ANR exhibited a negative relationship with each other (**Figure 11B**).

Isolates almost from every rhizosphere exhibited an association with GE. Some of the isolates derived from the rhizosphere of *A. nordmanniana*, *C. avellana*, *M. domestica*, and *A. pseudoplatanus* were linked to ERL, SVI2, ERM, SVI1, ESM, ESH, and ERS ratio (**Figure 11B**), and only a few of them possessed the ability to localize within the stem and root of rice plants. A few of the isolates from *F. carica* and *P. elaeagnifolia* were also associated with ESM and ESH. Similarly, some isolates derived from *C. avellana*, *P. elaeagnifolia*, and *R. pseudoacacia* were linked with ANR with a few possessing the ability to colonize rice stem (**Figure 11B**).

### Principal component analysis based on growth traits of rice upon root inoculation

**Figure 12:**
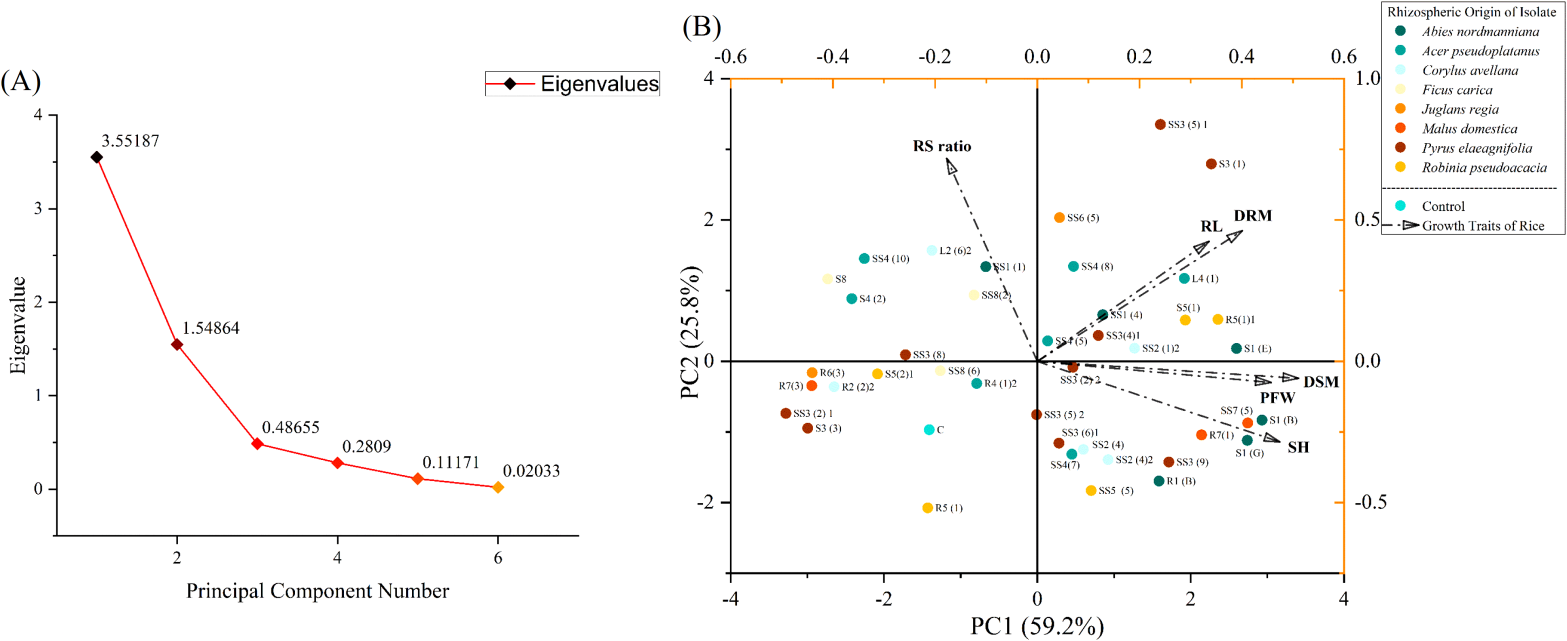
Principal component analysis (PCA) of bacterial isolates based on their impact on the growth of 21-day-old rice seedlings in a hydroponic culture: **(A)** scree plot between eigenvalues and principal components (PCs); **(B)** biplot between PC1 and PC2.

PCA revealed that PC1 greatly contributed (59.2%) to elucidate the variability in growth parameters of rice upon bacterial inoculation followed by PC2 (25.8%), PC3 (8.1%), PC4 (4.7%), and PC5 (1.9%). The cumulative percentage of variance together for first five PCs was 99.7% along with the first two PCs presenting eigenvalues >1 (**Figure 12A**). PC1 was positively influenced by all growth traits (RL, DRM, DSM, PFW, SH) except the RS ratio. PC2 was positively influenced by RL, DRM, and RS ratio, while it was negatively affected by DSM, PFW, and SH (**Figure 12B**).

The biplot represents each growth trait with a unique vector starting from the origin in relation with bacterial isolates of different rhizospheres. Furthermore, the biplot described almost 85% of the variations in data based on PC1 and PC2 as shown in **Figure 12B**. All the vectors spread away from the origin with varying magnitudes representing a high degree of variability in growth indices upon the application of different bacterial isolates. Isolates S3(1), L4(1), and R5(1)1 were highly associated, SS4(8), S5(1), SS1(4), and SS3(4)1 were moderately associated, while SS2(1)2 and SS4(5) were slightly associated with RL and DRM. Both RL and DRM vectors were almost aligned in the same direction and showed high interrelationship with each other (**Figure 12B**). Isolates S1(E), S1(B), S1(G), SS7(5), and R7(1) demonstrated high association with DSM, PFW, and SH. Moreover, SS3(9), SS2(4)2, R1(B), and SS2(4) were found to be moderately associated with DSM, PFW, and SH, while SS3(5)2, SS3(6)1, SS4(7), and SS3(2)2 were slightly linked to DSM, PFW, and SH. Three of the vectors (DSM, PFW, and SH) showed a very close interrelationship with each other. On the other side of the biplot, the root shoot ratio of seedlings was found to be moderately associated with isolates L2(6)2, SS1(1), and SS8(2), while it was slightly linked to SS4(5). Moreover, the RS ratio vector was more or less at the 90° angle associated with RL and DRM (**Figure 12B**).

Some of the isolates derived from the rhizosphere of *A. pseudoplatanus*, *P. elaeagnifolia*, and *R. pseudoacacia* were linked to RL and DRM (**Figure 12B**). Most of the isolates derived from *A. nordmanniana*, *M. domestica*, and *P. elaeagnifolia* were associated with DSM, PFW, and SH (**Figure 12B**). Similarly, a few isolates derived from *C. avellana*, *A. nordmanniana*, and *F. carica* were linked to the seedling RS ratio and average numbers of roots (**Figure 12B**). Some of the isolates from these rhizospheres were confirmed for their endophytic nitrogen fixation within the leaf, stem, and root of rice plants.

### Cluster hierarchical analysis

**Figure 13:**
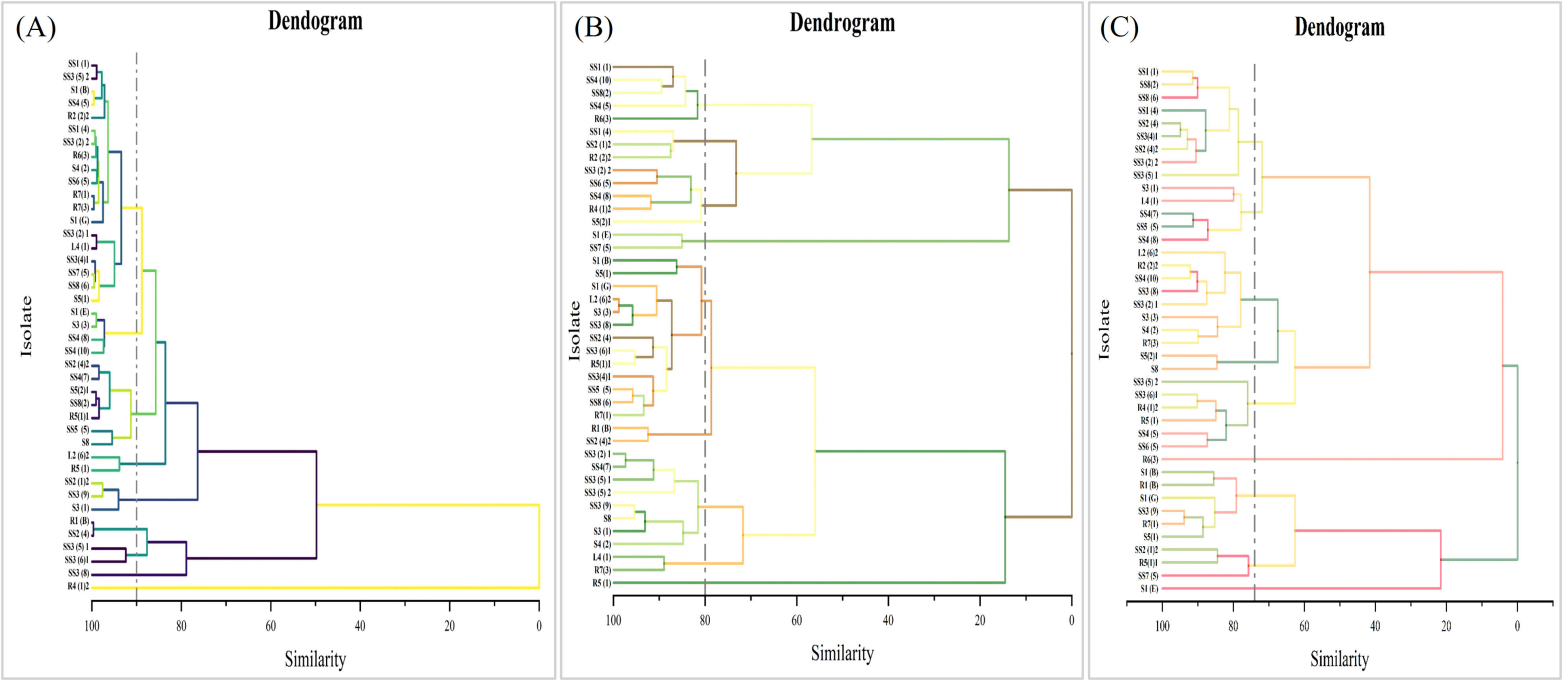
Dendograms of cluster hierarchical analysis showing the similarities in clusters of bacterial isolates based on **(A)** PGP traits, **(B)** germination response of rice to seed biopriming, and **(C)** growth response of seedlings in a hydroponic system following root inoculation. The colored lines connect the different clusters with each other.

Cluster hierarchical analysis of isolates derived from the rhizosphere of different trees was performed on the basis of different PGP traits (i.e., IAA, SDPs, PS, amylase, protease, cellulase, NH_3_, HCN), germination traits (i.e., GE, ESH, ERL, ESM, ERM, SVI1, SVI2, Avg. no. of roots), and growth traits (i.e., RL, DRM, DSM, PFW, SH, seedlings RS ratio). Cluster analysis of PGP traits grouped the 41 isolates into nine clusters at 90% similarity (**Table 10**). Cluster I contained 19 isolates followed by 4, 7, 2, 3, 2, 2, 1, and 1, respectively, in clusters II–IX (**Figure 13A**). Cluster analysis of germination traits gathered the 41 isolates into nine clusters at 80% similarity (**Table 11**). Cluster I enclosed 5 isolates followed by 3, 5, 2, 13, 2, 8, 2, and 1, respectively, in clusters II–IX (**Figure 13B**). Cluster analysis of growth traits grouped the 41 isolates into nine clusters at 74% similarity (**Table 12**). Cluster I contained 9 isolates followed by 5, 8, 2, 6, 1, 3, and 1, respectively, in clusters II–IX (**Figure 13C**).

**Table 10:** Hierarchical clustering of bacterial isolates based on plant growth-promoting traits.

Cluster	Isolate
I	SS(1), SS3(5)2, S1(B), SS4(5), R2(2)2, SS1(4), SS3(2)2, R6(3), S4(2), SS6(5), R7(1), R7(3), S1(G), SS3(2)1, L4(1), SS3(4)1, SS7(5), SS8(6), S5(1)
II	S1(E), S3(3), SS4(8), SS4(10)
III	SS2(4)2, SS4(7), S5(2)1, SS8(2), R5(1)1, SS5(5), S8
IV	L2(6)2, R5(1)
V	SS2(1)2, SS3(9), S3(1)
VI	R1(B), SS2(4)
VII	SS3(5)1, SS3(6)1
VIII	SS3(8)
IX	R4(1)2

**Table 11:** Hierarchical clustering of bacterial isolates based on their effects on germination traits of rice following seed biopriming.

Cluster	Isolate
I	SS1(1), SS4(10),SS8(2), SS4(5), R6(3)
II	SS1(4), SS2(1)2, R2(2)2
III	SS3(2)2, SS6(5), SS4(8), R4(1)2
IV	S1(E), SS7(5)
V	S1(B), S5(1), S1(G), L2(6)2, S3(3), SS3(8), SS2(4), SS3(6)1, R5(1)1, SS3(4)1, SS5(5), SS8(6), R7(1)
VI	R1(B), SS2(4)2
VII	SS3(2)1, SS4(7), SS3(5)1, SS3(5)2, SS3(9), S8, S3(1), S4(2)
VIII	L4(1), R7(3)
IX	R5(1)

**Table 12:** Hierarchical clustering of bacterial isolates based on their impact on growth traits of rice seedlings following root inoculation.

Cluster	Isolate
I	SS1(1), SS8(2), SS8(6), SS1(4), SS2(4), SS3(4)1, SS2(4)2, SS3(2)2, SS3(5)1
II	S3(1), L4(1), SS4(7), SS5(5), SS4(8)
III	L2(6)2, R2(2)2, SS4(10), SS3(8), SS3(2)1, S3(3), S4(2), R7(3)
IV	SS5(2)1, S8
V	SS3(5)2, SS3(6)1, R4(1)2, R5(1), SS4(5), SS6(5).
VI	R6(3).
VII	S1(B), R1(B), S1(G), SS3(9), R7(1), S5(1).
VIII	SS2(1)2, R5(1)1, SS7(5).
IX	SS1(E).

## Discussion

In the present study, we isolated different bacterial strains from the rhizospheres of eight different trees from Uzungöl forest located near the coast of Black Sea in Trabzon, Turkey. We screened out these isolates for PGP traits and evaluated their impacts on germination and growth of rice. We expected that bacterial isolates derived from the rhizosphere soils of forest trees would present better PGP traits. As the forest ecosystem holds more diverse microbial communities adapted to natural conditions, therefore, this microbial-rich ecosystem would provide us plenty of microorganisms having unique features to be used as plant growth stimulators, biofertilizers, and biocontrol agents. We found a number of isolates with multiple PGP traits (IAA, SDPs, PS, N-fixation, etc.) that can be used as bioinoculants to promote germination and growth of rice (**Tables 4**, **5**).

We examined the morphological features of bacterial colonies, and the trend in percentage frequency of different colony forms varied across the isolates obtained from *A. nordmanniana*, *A. pseudoplatanus*, *J. regia*, and *M. domestica*; however, they were mostly dominated by circular form (**Table 3**). Isolates from *C. avellana* and *F. carica* exhibited a similar trend in colony form. Parallel to this, isolates from *P. elaeagnifolia* and *R. pseudoacacia* also showed the same trend in colony form. White color was the most dominant among the isolates, except those from *A. nordmanniana* and *J. regia* (yellow dominant). Creamy color was the second most abundant after yellow and transparent was the rarest. Raised elevations were the most abundant (following umbonate and flat) in isolates except those from *A. nordmanniana* (convex dominated) and *R. pseudoacacia* (umbonate dominated). Entire margins were the most frequent followed by curled, undulate, and filiform. These findings are similar to a study conducted on the isolation of PGPR from agriculture soils found to be dominant in terms of circular form, white color, raised elevations, and entire margins in morphology of microbes derived from the rhizospheres of different crops. Moderate to high variations in morphological features of microbes across rhizospheres of different trees can be traced back to several factors, i.e., pH of the rhizosphere, organic matter contents, nutrient variability, tree-specific root exudates, and overall soil health. These factors together with other environmental factors shaped a specific combination of microbes possessing diverse features in a particular rhizosphere (Seitz et al., 2022; Fierer and Jackson, 2006). Similarities in root exudate profiles and soil chemistry across different rhizospheric environments may favor certain microbial communities of alike morphological traits. Thus, integrating morphological features with PGP traits, genomic profiling, and host interaction studies can serve as a valuable approach for screening effective PGPR candidates in future biofertilizer development programs. However, it is important to recognize that morphological traits do not always correlate directly with plant growth-promoting activity or ecological function, necessitating the need for complementary physiological, molecular, and genomic characterization.

We estimated the abundance of IAA-producing bacteria in different rhizospheres along with their IAA-producing capacities and found that the abundance of IAA producers varied from 57.1% to 100% across different rhizospheres (**Tables 4**, **5**). The highest abundance (100%) was observed from *A. nordmanniana* and *C. avellana*, while the least abundance of IAA producers (57.1%) was observed from *J. regia* and *M. domestica*. Generally, the production of IAA by PGPR stimulates cell division, root elongation, and differentiation. It leads to better growth of root hairs, high nutrient uptake, and more yield (Pappalettere et al., 2024; Lata et al., 2024; Abd El-Mageed et al., 2022; Myo et al., 2019). However, very high IAA-producing bacteria may reduce root elongation and enhance shoot:root ratio (Myo et al., 2019). Of the 129 isolates, 31 exhibited IAA production above 100 µg/mL, 9 isolates above 200 µg/mL, and 6 isolates (R1(B), SS3(5), SS3(5)1, SS3(6)1, SS3(8), and R4(1)2) produced above 300 µg/mL (**Table 5**, **Figure 3A**). Most of the isolated and identified bacterial strains of this work are previously known to produce IAA but in a lower quantity as compared to our strains (Andreozzi et al., 2019; Thakur et al., 2017; Mona et al., 2016; Xu et al., 2018, 2020; Vitorino et al., 2024; Parashar et al., 2023; Abd El-Mageed et al., 2022; Kumar and Singh, 2018; Kang et al., 2009; Kabiraj et al., 2024; Tian et al., 2017; Shirmohammadi et al., 2020). *Bacillus altitudinis* strain R4(1)2 stands out as a potent IAA-producing endophyte, demonstrating one of the highest levels (739.9 ± 251.5 µg/mL) reported in the literature, particularly when compared to previously reported highest level of 221.7 µg/mL, achieved with the addition of selenium nanoparticles (Li et al., 2024). Meanwhile, other strains of *B. altitudinis* isolated from different agroecosystems have shown little to no IAA production (Kumar et al., 2017; Habibi et al., 2014). *Bacillus altitudinis* strain R4(1)2 may have a highly efficient tryptophan-dependent IAA biosynthesis pathways or potentially alternative regulatory mechanisms that enhance auxin production due to its ecological relevance. Further investigations are needed to assess its performance in soil–plant systems and to understand the regulatory pathways underlying this high IAA yield along with integration of genomic and metabolomic profiling. Besides their role as growth stimulators, microbes with high IAA production can be optimized and engineered for large-scale industrial applications. These microorganisms could be employed to develop IAA-based herbicides that overstimulate plant growth, offering a biological substitute to chemical herbicides (Bunsangiam et al., 2021). Some previous studies have already demonstrated effective weed control with the application of bacteria having high IAA production (Sarwar and Kremer, 1995).

We also investigated the presence of endophytic bacteria that can colonize inside rice plant tissues. Of the 129 bacterial isolates from eight different rhizospheres, 50.4% were endophytes of rice having the potential to fix N in plants (**Tables 4**, **5**). The peak abundance of endophyte bacteria (73.7%) was observed from the *A. nordmanniana* rhizosphere, while the least amount was recorded from *F. carica* (14.3%). These findings demonstrated the distribution and occurrence of endophytic N-fixing bacteria of rice across different forest rhizospheres outside of the agriculture ecosystem. These bacteria can be used as a biofertilizer (Rana et al., 2023), enhancing nitrogen use efficiency in rice by supplementing nitrogen (Harahap et al., 2023). It will limit the excessive use of inorganic fertilizers, which often results in nitrogen losses through NH_3_ volatilization, nitrous oxide emissions, and nitrate leaching (Jote, 2023). However, it is important to quantify the N-fixing capacity of a particular strain before recommending its use as a nitrogen biofertilizer in crop production. A large number of endophytic N-fixing bacteria have already been isolated in previous studies from agriculture soils and showed positive impacts on growth and yield of cereal crops especially rice (Quadriya et al., 2024; Çağlar et al., 2024; Tran et al., 2024; Habibi et al., 2014). Future studies should focus on quantifying the nitrogen-fixing capacity of these bacteria to identify the most effective strains for biofertilizer development. Our *H. huttiense* strain S1(E) exhibited potential for endogenous nitrogen fixation, consistent with a previous study that reported high nitrogen fixation and *nifH* gene expression of *H. huttiense* in *O. sativa*. The genus *Herbaspirillum* is well known for its endogenous *L. amnigena* nitrogen-fixing ability (Matteoli et al., 2020; Zakria et al., 2007). Other isolates, i.e., strain S3(1), *B. altitudinis* strain R4(1)2, *A. calcoaceticus* strain R5(1)1, and *Lelliottia* sp. strain R7(1) also exhibited potential for endogenous nitrogen fixation in rice, similar to earlier reports confirming the N-fixation by *L. amnigena*, *B. altitudinis*, and *A. calcoaceticus* (Parashar et al., 2023; Habibi et al., 2014; Liba et al., 2006; Rasul et al., 2019). In a previous study, *B. altitudinis* strain JR4 has demonstrated nitrogen-fixation activity in both *in-vitro* and *in-vivo* studies following inoculation of rice plants. In contrast, the same study reported JR198, another strain of *B. altitudinis*, with no such activity (Habibi et al., 2014). Although *H. huttiense* strain S1(E), *L. amnigena* strain S3(1), *B. altitudinis* strain R4(1)2, *A. calcoaceticus* strain R5(1)1, and *Lelliottia* sp. strain R7(1) can grow on nitrogen-free media, their nitrogen-fixation capacities remain unknown and warrant further investigation through controlled *in-vitro* and *in-vivo* assays. The variability in nitrogen-fixation capacity among different endophytic PGPR strains is well documented and highlights the need to understand the genetic and regulatory factors controlling this functional diversity. Parallel to this, it is important to further investigate the impacts of these microorganisms on soil and crop nitrogen dynamics through soil incubation studies and field experiments. Also, genetic manipulation of these microorganisms can help to boost their N-fixing potential.

Ammonia production is another beneficial trait of PGPR bacteria. Ammonia-producing bacteria contribute to the nitrogen cycle by converting organic nitrogen or amino compounds into ammonia, a process that may also stimulate the mineralization of organic matter in the rhizosphere (Jiang et al., 2013). This function is particularly beneficial in high organic matter soils, where nitrogen mineralization is critical for nutrient availability. In such environments, microbial-driven ammonification can serve as a key mechanism to supply plant-available nitrogen, especially when synthetic nitrogen inputs are limited or substituted with organic sources. We discovered that isolates from *F. carica* (71.4%) and *P. elaeagnifolia* (58.5%) showed higher frequencies of ammonia producers (**Tables 4**, **5**). Similar to our study, many PGPR strains have previously been identified as ammonia producers (Alotaibi et al., 2022). Ammonia-producing PGPR bacteria have a significant impact on soil nitrogen dynamics especially in rice where the preferable form for nitrogen uptake is ammonium NH_4_^+^ instead of nitrate NO_3_^−^ (Quadriya et al., 2024). Among the identified bacterial strains, ammonia production was detected in *P. faecigallinarum* strain SS2(1)2. To our knowledge, this is the first report of ammonia production by *P. faecigallinarum*. While this bacterium has not previously been studied as a PGPR, members of the same genus *Psychrobacillus* have shown plant growth-promoting capabilities in past studies (Mona et al., 2016; Xu et al., 2018). This highlights its potential involvement in ecological nitrogen turnover and organic matter decomposition.

Phosphorous is the second most important plant nutrient after nitrogen. Bacteria capable of solubilizing inorganic forms of phosphorous may improve plant growth and nutrient uptake. These bacteria increase crop phosphorous use efficiency by converting soil-bound phosphorous into a form readily available to plants, while also preventing the buildup of phosphorous toxicity in soil. However, in our study, these microbes were relatively less abundant than IAA and siderophore producers. Our findings align with previous studies showing calcium phosphate solubilization as a relatively rare trait among PGPR (Habibi et al., 2014). The highest abundance (21.4%) of phosphate-solubilizing bacteria was recorded from the rhizosphere of *R. pseudoacacia* (**Tables 4**, **5**, **Figure 3C**). These findings demonstrated how the characteristics of rhizospheric bacteria influence the phosphorous dynamics across different rhizospheres. Our isolated bacterial strains SS2(4)2, SS3(4)1, SS3(6)1, SS4(7), S5(2)1, and S5(1); *A. calcoaceticus* R5(1)1; *Lelliottia* sp. R7(1); and *S. succinus* SS8(2) showed P solubilization. Among these bacteria, *A. calcoaceticus* and *Lelliottia* sp. are already known to solubilize calcium phosphate (Kang et al., 2009; Jeon et al., 2024). *Acinetobacter calcoaceticus* YC-5a isolated from fruit tree rhizosphere soil of a karst rocky desertification region has demonstrated a strong ability for solubilizing insoluble phosphate by producing organic acid (Xie et al., 2021). Similarly, another *A. calcoaceticus* strain ICA04Ma isolated from Mexicali Valley, a semi-arid agricultural region in the northwest of Mexico, also demonstrated P solubilization with a pH drop to 4.3 (Moreno-Ramirez et al., 2014). Our study reported for the first time the isolation of an *A. calcoaceticus* strain from the Uzungöl forest, a region that receives high annual rainfall and represents a humid temperate ecosystem. This finding adds valuable insight into the understanding of ecological distribution and wide adaptability of this bacteria in different climatic zones ranging from arid to humid forest. Our isolated strains warrant further investigations under field conditions, as many bacteria in the past have demonstrated positive results, when applied as phosphatic biofertilizers (Kang et al., 2009).

The production of iron-chelating siderophores is another important feature of PGPR. Siderophores play a vital role in plant defense, primarily through their involvement in iron acquisition. Siderophores sequester iron in the soil due to their high affinity for Fe^3+^ and limit pathogen growth by creating iron-deprived conditions. In the present investigation, siderophore production was also studied in bacterial isolates. Only a small fraction of isolates were found positive for this trait. The highest 28% abundance of siderophore-producing PGPR was recorded from *R. pseudoacacia* with the R5(1) strain producing maximum (91.1% ± 0.8%), while none of the isolates from *A. nordmanniana* and *J. regia* exhibited siderophore production (**Tables 4**, **5**, **Figure 3B**). These findings highlight the role of plant–microbe interactions in strengthening the plant defense capabilities across different rhizospheres. Among the identified bacteria, *L. fusiformis* strain SS3(2)2, *M. phyllosphaerae* strain SS4(8), and *Lelliottia* sp. strain R7(1) exhibited siderophore production, consistent with previous studies (Abd El-Mageed et al., 2022; Jha and Mohamed, 2023; Jeon et al., 2024). From an applied perspective, these strains are promising candidates for the biocontrol of plant diseases, aimed at reducing chemical pesticide use. However, it is also noteworthy that siderophore production alone does not guarantee biocontrol efficacy, and it represents a key mechanism within a broader set of plant-beneficial traits. Therefore, future investigations should include *in-vivo* trials to assess disease suppression capabilities of most promising strains. Bacteria similar to our strains have previously been reported to effectively control various plant diseases (Liang et al., 2024; Chen et al., 2023). *In-vitro* and *in-vivo* studies showed that *L. fusiformis* strain S4C11, isolated from apple tree roots in northern Italy, exhibited antifungal activity (Passera et al., 2021). *Microbacterium phyllosphaerae* has been reported to exhibit high biocontrol efficacy against Chinese plum anthracnose (Corretto et al., 2020; Chen et al., 2023). *Lelliottia* sp. JS-SCA-14 has shown dual traits of P solubilization and siderophore production (Jeon et al., 2024). *In-vitro* trials with endophytic bacteria from ginger demonstrated that *L. amnigena* J29 exhibited strong antifungal activity, inhibiting *Botrytis cinerea* growth by over 94% and *Colletotrichum acutatum* by over 74% (Bódalo et al., 2023).

HCN production by PGPR helps to suppress pathogens by disrupting electron transport chain in cellular respiration leading to energy depletion and eventual death of pathogens. HCN also increases the availability of nutrients like phosphorous and iron to plants, which improve nutrient uptake and growth. In the current investigation, the abundance of HCN-producing PGPR varied from 16.7% to 50% depending on the rhizosphere (except *M. domestica* or *F. carica*; no HCN producer was found). In parallel with other PGP traits, we identified isolates producing HCN ranging from low (+), moderate (++) to high (+++) in concentration (**Tables 4**, **5**). These results are similar to studies that demonstrated HCN production by PGPR in addition to IAA, siderophore, and phosphate solubilization (Mir et al., 2022). Our study helped to understand how HCN production by PGPR could possibly shape plant defense and nutrient profiles across different rhizospheres. HCN-producing bacterial isolates need further investigations to check their effectiveness in sustainable biological control of soil-borne pathogens of crops.

Similar to siderophore and HCN production, PGPR play a vital role in plant disease management by releasing extracellular hydrolytic enzymes. These enzymes disrupt the cell wall of pathogenic bacteria and fungi by targeting the glycolytic linkages of the cell wall. Additionally, extracellular enzymes of PGPR have a profound impact on the fertility and decomposition of organic matter in the soil; proteolytic bacteria break down proteins into small fragments, which is crucial for the release of nitrogen; cellulolytic bacteria decompose plant debris by stimulating cellulose degradation in the carbon cycle, while amylolytic bacteria contribute to nutrient cycling by hydrolyzing polysaccharides into simple sugars (Reddy et al., 2022). We found the high abundance of proteolytic bacteria from the rhizospheres of *F. carica* (71.4%) and *A. pseudoplatanus* (70%). Proteolytic bacteria were present in all rhizospheres; however, their abundance fluctuates from 20% to 71% depending on the root zone (**Tables 4**, **5**, **Figure 4A**). Proteolytic activity varied among the isolates from 1.2 ± 0 in strain SS1(4) to 5.5 ± 0.6 in strain SS6(5). Parallel to this, cellulolytic bacteria were the most abundant (71.4%) among the isolates from *F. carica*. Their abundance varied from 10.3% to 71.4% in a rhizosphere-dependent manner, except *J. regia* and *M. domestica* where no cellulolytic activity was reported (**Tables 4**, **5**, **Figure 4C**). Among the extracellular hydrolytic enzymatic activities, amylolytic activity was the least abundant among the isolates. The highest abundance (30%) was from *A. pseudoplatanus* (**Tables 4**, **5**, **Figure 4B**). These findings illustrated the possible role of PGPR in the breakdown of macromolecules, i.e., protein, cellulose, and starch across different rhizospheres along with their protective role against controlling pathogenic microbes. These extracellular enzyme-producing bacteria could provide several potential benefits to plants such as soil fertility improvement by stimulating soil organic matter decomposition, control of pathogens, and bioremediation of soil pollutants (Saeed et al., 2021).

In the present study, we tested 41 bacterial isolates possessing significant PGP traits to see their effects on rice germination and seedling growth. We also demonstrated their efficacy in hydroponic rice cultivation. Majority of the isolates showed positive results in improving germination and growth of rice. However, we performed 16S rRNA identification for the most promising strains which promoted both germination and seedling growth.

We found that seed biopriming and root inoculation of rice with the *H. huttiense* subsp. *huttiense* strain S1(E) exhibited outstanding performance during germination and growth test due to its effective colonization, IAA production, and potential N-fixation (**Figures 5**, **8D**, **9**; **Tables 5**, **7**, **8**). This finding is similar to a study that showed *H. huttiense* increased the dry weight, chlorophyll, and nitrogen contents of *O. sativa* through IAA production and N-fixation (Andreozzi et al., 2019). *Viridibacillus arenosi* strain SS1(4) with high IAA production from the *A. nordmanniana* rhizosphere was also found to be an effective PGPR for promoting germination and growth of rice (**Figures 5**, **9**; **Tables 5**, **7**, **8**) as demonstrated by earlier studies that *V. arenosi* from the tea rhizosphere exhibited potential broad spectrum plant growth-promoting activities as its application incremented plant height, leaf number, and leaf weight in tea plants along with yield improvement in pea and wheat (Thakur et al., 2017). *Psychrobacillus faecigallinarum* strain SS2(1)2 isolated from the *C. avellana* rhizosphere was effective in promoting the germination of rice (**Figures 5**, **9**; **Tables 5**, **7**, **8**). This is the first report showing that inoculation of rice with the *P. faecigallinarum* strain SS2(1)2 resulted in better germination and seedling growth. Parallel to this, *Psychrobacillus psychrodurans*, the closest member of *P*. *faecigallinarum*, has previously been known to improve growth in barley and wheat (Mona et al., 2016; Xu et al., 2018). *Lysinibacillus fusiformis* strain SS3(2)2, isolated from the *P. elaeagnifolia* rhizosphere, incremented germination and growth parameters of rice due to its IAA production and siderophore formation (**Figures 5**, **9**; **Tables 5**, **7**, **8**). Previous studies reported that *L. fusiformis* biofertilizer not only stimulate growth of axillary buds in rice but also increment root growth and yield of soybean (Xu et al., 2020; Vitorino et al., 2024). Another isolate *L. amnigena* strain S3(1) from the *P. elaeagnifolia* rhizosphere promoted seedling growth particularly via root inoculation (**Figures 5**, **9**; **Tables 5**, **8**). This finding is supported by evidence that seed priming with *L. amnigena* improved germination and growth profile in wheat and tomato along with remarkable yield increment in field trials (Parashar et al., 2023). Seed biopriming and root inoculation with *Bacillus siamensis* strain SS4(5), isolated from the *A. pseudoplatanus* rhizosphere, enhanced germination traits and seedling growth of rice (**Figures 6**, **9**; **Tables 5**, **7**, **8**), consistent with the studies showing *B. siamensis* increased rice growth in both a nitrogen-deprived environment and salt stress (Oubaha et al., 2024; Rios-Ruiz et al., 2023). *Microbacterium phyllosphaerae* strain SS4(8) from *A. pseudoplatanus* exhibited a significant increase in rice seedling vigor index, germination energy, dry shoot, and root masses along with high IAA production and siderophore formation (**Figure 6**; **Tables 5**, **7**), similar to prior experiments demonstrating that biopriming with *M. phyllosphaerae* enhanced dry mass, grain weight, and root length in wheat through multiple PGP traits (Kumar and Singh, 2018). *Bacillus altitudinis* strain R4(1)2, isolated from *A. pseudoplatanus*, significantly improved the rice germination traits when used as a biopriming agent attributed to its high IAA production, N-fixation, protease, and cellulase activity (**Figure 6**; **Tables 5**, **7**). This finding is consistent with a study demonstrating increased root and shoot length in germinating rice seedlings following seed treatment with *B. altitudinis* (Rangjaroen et al., 2019). Phosphate-solubilizing bacteria *A. calcoaceticus* strain R5(1)1 from the *R. pseudoacacia* rhizosphere significantly contributed to emerging root mass, plant fresh weight, shoot height, dry shoot, and root masses of rice (**Figures 5**, **9**; **Tables 5**, **8**), similar to a study showing that gibberellins (GA) producing *A. calcoaceticus* promoted the growth of cucumber, Chinese cabbage, and crown daisy (Kang et al., 2009). Interestingly, although strain R5(1) exhibited a broad spectrum of plant growth-promoting (PGP) traits including high siderophore production, IAA synthesis, nitrogen fixation, ammonia and HCN production, and cellulase activity, it demonstrated a negative impact on rice germination following seed biopriming (**Figure 7**, **Tables 5**, **7**). This unexpected result highlights the complexity of plant–microbe interactions, particularly at early developmental stages. One possible explanation is that very high siderophore activity may have potent cellular effects which disrupt the iron homeostasis of organelles, activating transcription pathways in a way that collaterally stresses the rice seed microenvironment. Despite its negative impact on rice germination, strain R5(1) may hold promise as a biocontrol agent in other crops. Moreover, its potential N-fixation ability and ammonia production indicate its role in nitrogen cycling and soil fertility dynamics. *Micrococcus luteus* strain SS6(5) with IAA and high protease activity from the *J. regia* rhizosphere significantly incremented emerging root mass, seedling vigor index, and dry mass of rice via seed biopriming (**Figure 7**; **Tables 5**, **7**). This aligns with a study demonstrating that proline-, IAA-, and GA-producing *M. luteus* isolated from arsenic-contaminated soils effectively promoted rice growth (Kabiraj et al., 2024). *Pseudomonas mohnii* strain SS7(5) from the *M. domestica* rhizosphere contributed to increased root, shoot masses, and vigor of emerging rice seedlings along with significant increment in different growth traits (see **Figures 7**, **9**; **Tables 5**, **7**, **8**). This is parallel to an experiment showing 1.5 times increment in plant growth with the application of *P. mohnii* (Tian et al., 2017). Another bacteria *Lelliottia* sp. strain R(7)1 from the *M. domestica* rhizosphere was an effective inoculum to promote germination and growth in rice (see **Figures 7**, **9**; **Tables 5**, **8**). This aligns with another study where *Lelliottia* sp. alleviated phosphorous deficiency symptoms and promoted germination and growth of tomato seedlings with notable features of phosphate solubilization and siderophore production (Jeon et al., 2024). Rice seed biopriming with *S. succinus* strain SS8(2) from the *F. carica* rhizosphere significantly improved germination traits (see **Figure 7**; **Tables 5**, **7**), consistent with a study showing that seed inoculation with *S. succinus* increased qualitative and quantitative indices of wheat (Shirmohammadi et al., 2020). Several bacterial strains identified in this study have not been previously reported as PGPR for rice. Their ability to enhance germination and early seedling development underscores their potential as novel growth stimulators or biofertilizer candidates, particularly in hydroponic systems where early root–shoot vigor is essential for plant establishment. These strains demonstrated successful colonization and growth promotion under hydroponic conditions with robust adaptability. Multiple PGPR traits exhibited by these isolates, such as IAA production, nitrogen fixation, phosphate solubilization, and siderophore production, are well-established mechanisms that support plant development and health. Although early-stage improvements in rice growth are significant indicators of PGPR effectiveness, they do not necessarily ensure enhanced productivity or stress resilience under open-field conditions. Environmental variability, complex soil microbiome dynamics, plant genotype interactions, and competition with native microbial communities may interfere with the efficacy of these strains beyond a controlled environment. Moreover, there remains a gap on how these strains affect reproductive stages, grain yield, or quality of rice, which are critical for their agronomic success and farmer adoption. Therefore, it is essential to conduct comprehensive field trials across multiple agro-ecological zones. Only through such validation can these promising PGPR candidates be confidently recommended for broader use in sustainable crop production.

## Conclusion

This study explores the novel insights into the beneficial traits of microbial communities of different rhizospheres from Uzungöl forest and reveals several bacterial strains with strong PGP capabilities. The practical applicability of these strains in rice cultivation was demonstrated through seed biopriming and root inoculation. The study also validates the efficacy of these strains in a hydroponic system to improve the early seedling vigor. Several bacterial strains, including the *H. huttiense* S1(E) and *P. mohnii* SS7(5), emerged as effective bioinoculants for rice. From a sustainable agriculture perspective, this work contributes to reduce the dependence on synthetic agrochemicals to promote crop establishment and early growth. The success of these forest-derived PGPR strains under controlled, soilless conditions suggests their potential applicability in modern agriculture systems such as vertical farming. For future research, we recommend genomic characterization of the most promising isolates to unravel the molecular basis of their PGP traits, host interaction, and potential stress resilience. Additionally, large-scale field experiments across multiple agroecological zones, coupled with comprehensive soil incubation studies on nutrient (N, P, Fe) mineralization and biocontrol efficacy trials, are suggested to validate their agronomic use. These investigations will help to assess their impacts on disease management, crop yield, and soil fertility. This study lays the foundation for developing innovative microbial-based strategies to enhance rice productivity, aligning with the sustainable agriculture goals.

## Data Availability

The datasets presented in this study can be found in online repositories. The names of the repository/repositories and accession number(s) can be found in the article/**Supplementary Material**.
