# Supplementary material for: Plant growth-promoting bacteria from Uzungöl forest stimulate rice growth via seed biopriming and root inoculation: isolation and functional characterization of potent PGPR strains from rhizosphere soils of different trees
Source: Front Plant Sci. 2025 Jul 24;16:1622951. doi: 10.3389/fpls.2025.1622951 (PMC12328432; doi:10.3389/fpls.2025.1622951)
Supplement: Supplementary file 1 [file DataSheet1.docx]

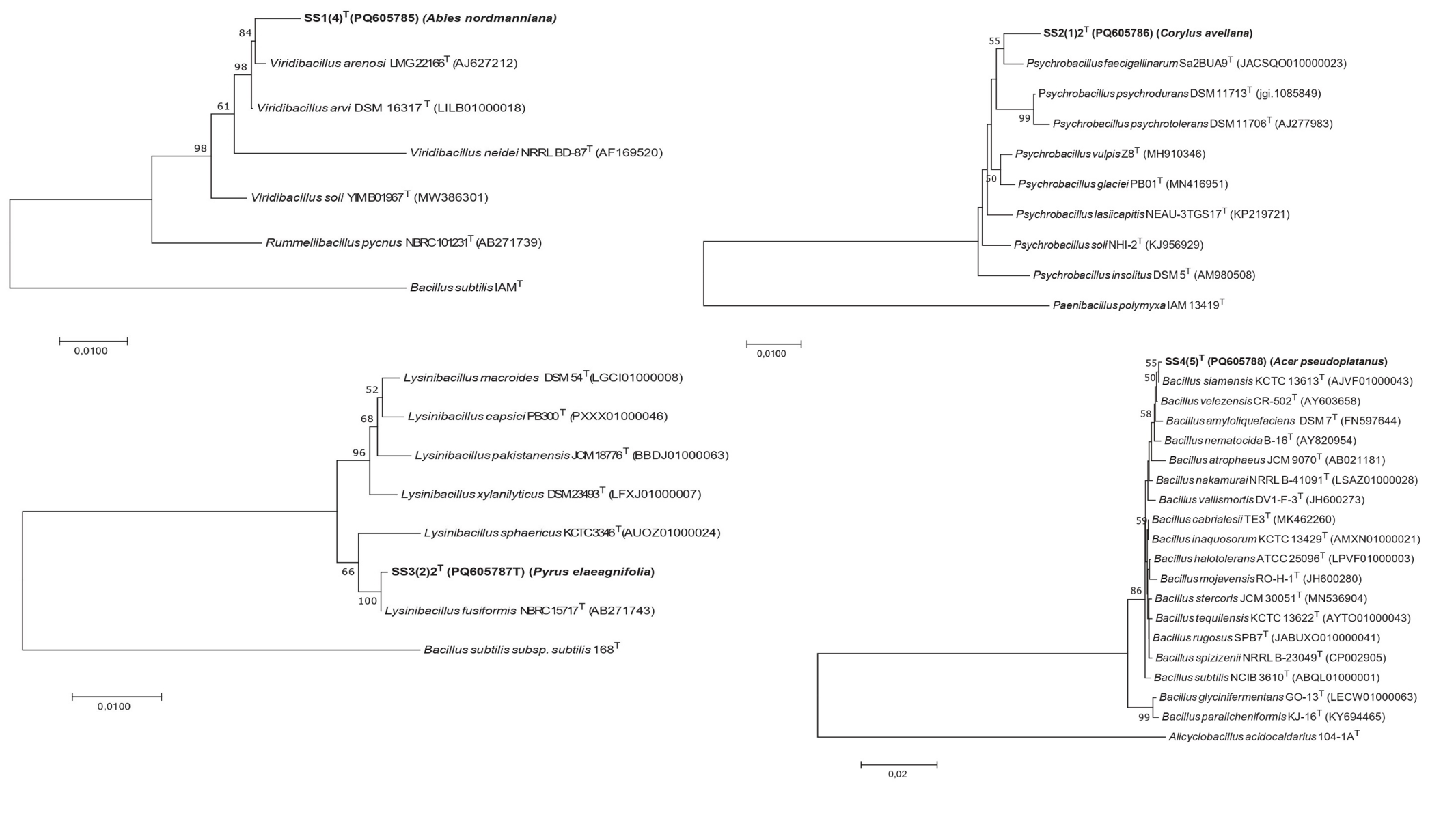


Figure 1S. Phylogenetic tree based on16S rRNA sequences showing positions of isolates (SS1(4), SS3(2)2, SS2(1)2, SS4(5)), and type strains of species in different genera along with bootstrap values, accession numbers of the 16S rRNA genes in NCBI, reference sequences in EzBioCloud and rhizospheric origins. Bootstrap values less than 50 are not included in tree.


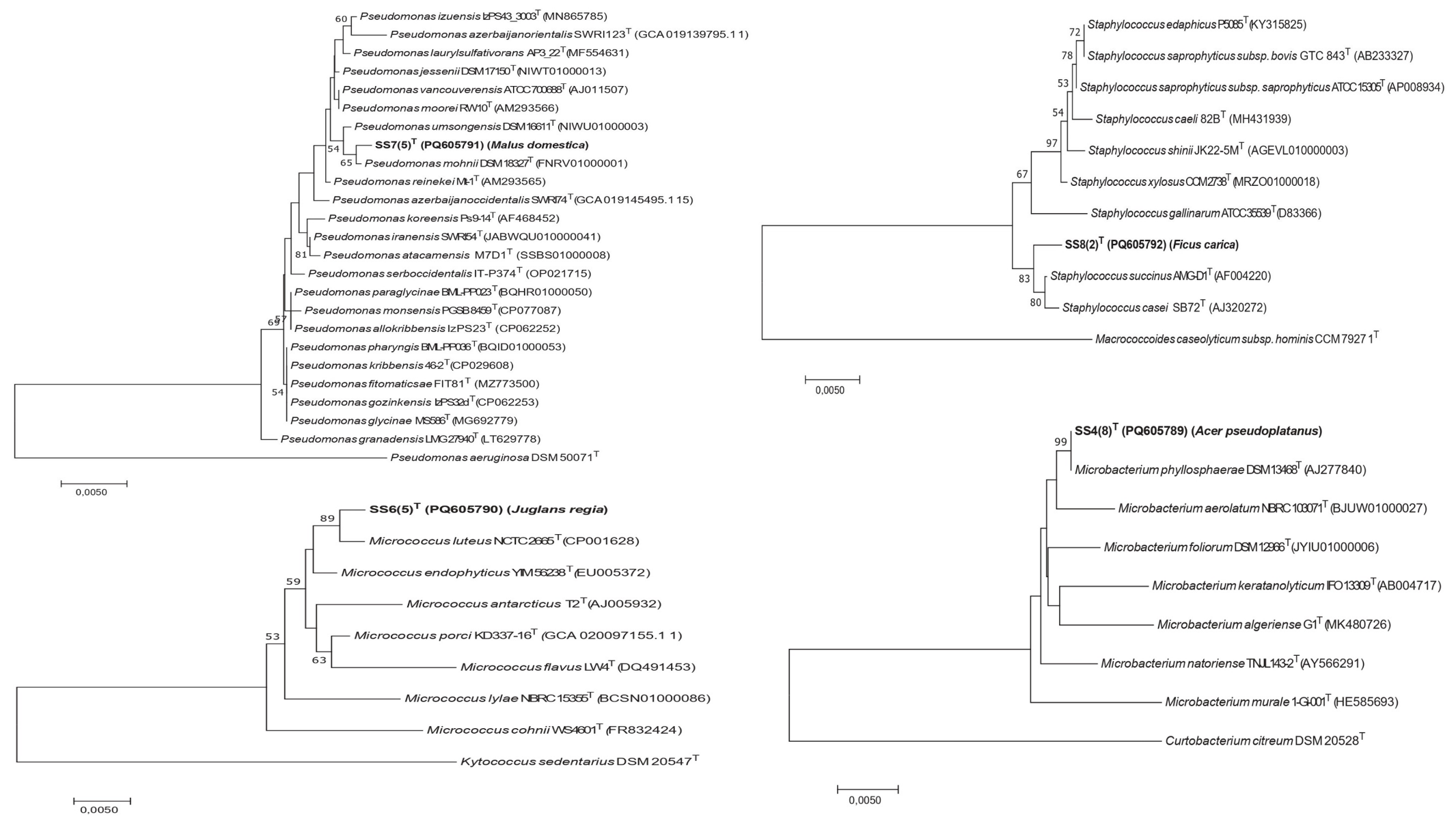


Figure 2S. Phylogenetic tree based on16S rRNA sequences showing positions of isolates (SS7(5), SS6(5), SS8(2), SS4(8)) and type strains of species in different genera along with bootstrap values, accession numbers of the 16S rRNA genes in NCBI, reference sequences in EzBioCloud and rhizospheric origins. Bootstrap values less than 50 are not included in tree.
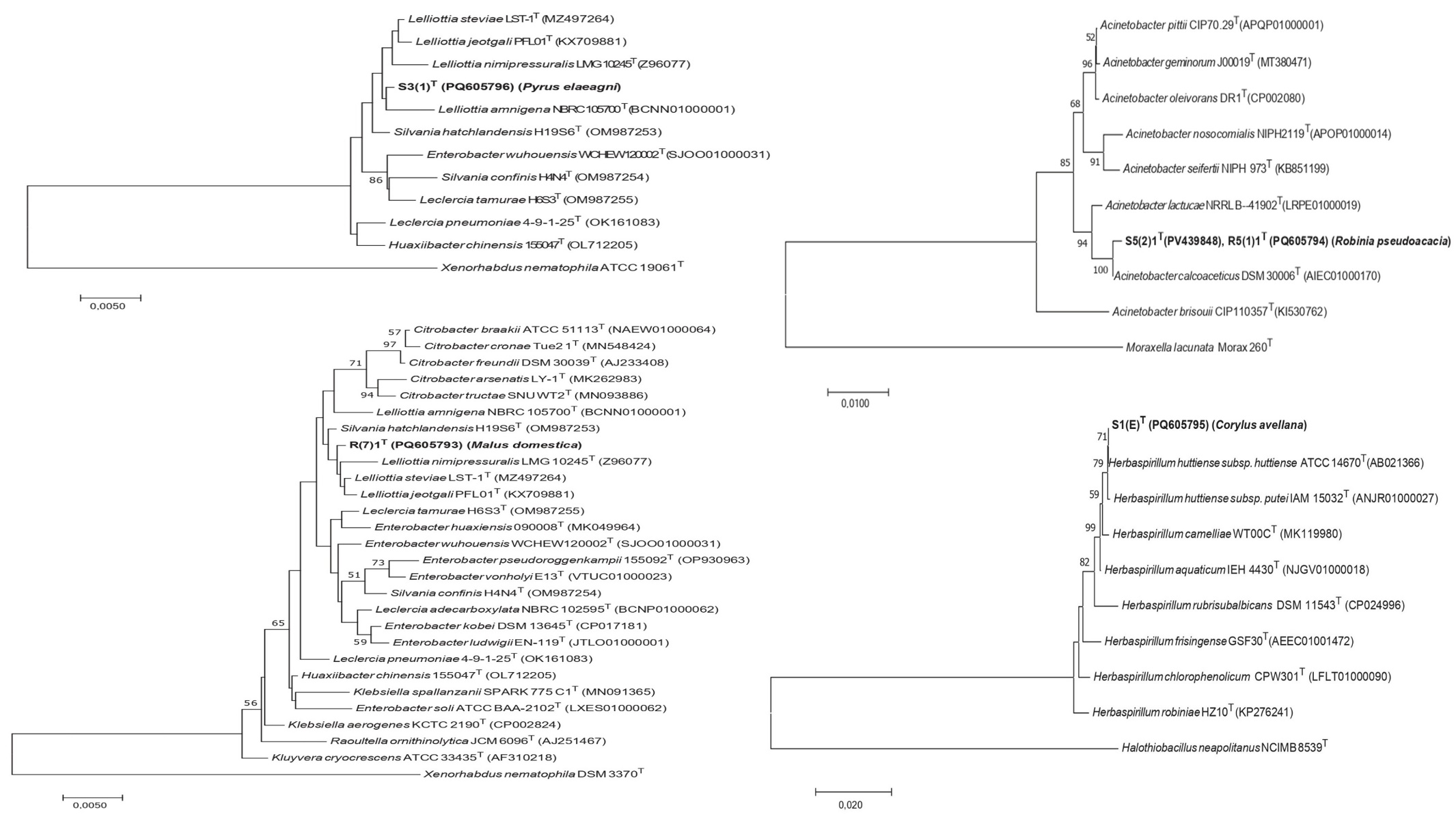


Figure 3S. Phylogenetic tree based on16S rRNA sequences showing positions of isolates (S3(1), R(7)1, S5(2)1, R5(1)1, S1(E)) and type strains of species in different genera along with bootstrap values, accession numbers of the 16S rRNA genes in NCBI, reference sequences in EzBioCloud and rhizospheric origins. Bootstrap values less than 50 are not included in tree.


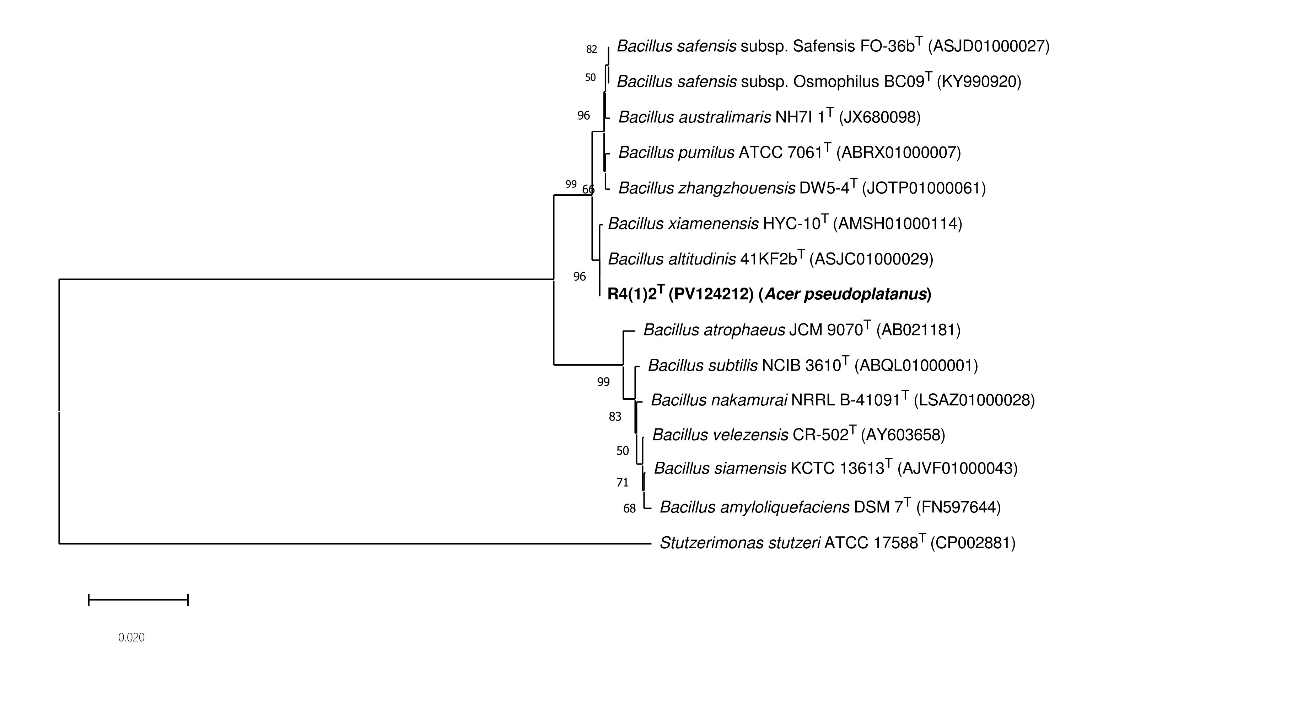


Figure 4S. Phylogenetic tree based on16S rRNA sequences showing positions of isolate R4(1)2 and type strains of species in different genera along with bootstrap values, accession numbers of the 16S rRNA genes in NCBI, reference sequences in EzBioCloud and rhizospheric origins. Bootstrap values less than 50 are not included in tree.
